# Canonical TGFβ signaling induces collective invasion in colorectal carcinogenesis through a Snail1- and Zeb1-independent partial EMT

**DOI:** 10.1038/s41388-022-02190-4

**Published:** 2022-01-24

**Authors:** Marion Flum, Severin Dicks, Yu-Hsiang Teng, Monika Schrempp, Alexander Nyström, Melanie Boerries, Andreas Hecht

**Affiliations:** 1 Institute of Molecular Medicine and Cell Research, Faculty of Medicine, University of Freiburg, 79104 Freiburg, Germany; 2 Spemann Graduate School of Biology and Medicine (SGBM), University of Freiburg, 79104 Freiburg, Germany; 3 Faculty of Biology, University of Freiburg, 79104 Freiburg, Germany; 4 Institute of Medical Bioinformatics and Systems Medicine, Medical Center - University of Freiburg, Faculty of Medicine, University of Freiburg, 79110 Freiburg, Germany; 5 Department of Dermatology, Medical Center - University of Freiburg, Faculty of Medicine, University of Freiburg, 79104 Freiburg, Germany; 6 Freiburg Institute for Advanced Studies (FRIAS), University of Freiburg, 79104 Freiburg, Germany; 7 German Cancer Consortium (DKTK), Freiburg, University of Freiburg, 79104 Freiburg, Germany; 8 German Cancer Research Center (DKFZ), 69120 Heidelberg, Germany; 9 BIOSS Centre for Biological Signalling Studies, University of Freiburg, 79104 Freiburg, Germany

**Keywords:** Cancer models, Colorectal cancer, Cadherins, Extracellular matrix, Growth factor signalling

## Abstract

Local invasion is the initial step towards metastasis, the main cause of cancer mortality. In human colorectal cancer (CRC), malignant cells predominantly invade as cohesive collectives and may undergo partial epithelial-mesenchymal transition (pEMT) at the invasive front. How this particular mode of stromal infiltration is generated is unknown. Here we investigated the impact of oncogenic transformation and the microenvironment on tumor cell invasion using genetically engineered organoids as CRC models. We found that inactivation of the *Apc* tumor suppressor combined with expression of oncogenic *Kras*^*G12D*^ and dominant-negative *Trp53*^*R172H*^ did not cell-autonomously induce invasion in vitro. However, oncogenic transformation primed organoids for activation of a collective invasion program upon exposure to the prototypical microenvironmental factor TGFβ1. Execution of this program co-depended on a permissive extracellular matrix which was further actively remodeled by invading organoids. Although organoids shed some epithelial properties particularly at the invasive edge, TGFβ1-stimulated organoids largely maintained epithelial gene expression while additionally implementing a mesenchymal transcription pattern, resulting in a pEMT phenotype that did not progress to a fully mesenchymal state. Notably, while TGFβ1 induced pEMT and promoted collective invasion, it abrogated self-renewal capacity of TKA organoids which correlated with the downregulation of intestinal stem cell (ISC) marker genes. Mechanistically, induction of the non-progressive pEMT required canonical TGFβ signaling mediated by Smad transcription factors (TFs), whereas the EMT master regulators Snail1 and Zeb1 were dispensable. Gene expression profiling provided further evidence for pEMT of TGFβ1-treated organoids and showed that their transcriptomes resemble those of human poor prognosis CMS4 cancers which likewise exhibit pEMT features. We propose that collective invasion in colorectal carcinogenesis is triggered by microenvironmental stimuli through activation of a novel, transcription-mediated form of non-progressive pEMT independently of classical EMT regulators.

## Introduction

Metastasis - accountable for the overwhelming majority of cancer-related deaths in solid cancers-requires that tumor cells successfully pass through a series of events summarily termed invasion-metastasis cascade [1]. Tumor cells can accomplish the initial step of this cascade by shedding cell-cell contacts and infiltrate adjacent stroma as individual cells, employing ameboid or mesenchymal modes of migration [2]. However, most human cancers display a collective mode of invasion where tumor cells maintain intercellular interactions and migrate in groups [2]. Nonetheless, cells at the invasive front of such a collective may also display mesenchymal features [2, 3]. Unfortunately, knowledge about the prerequisites and molecular determinants which promote a specific mode of invasion is limited.

Epithelial-mesenchymal transitions (EMT) are complex cellular programs that were repeatedly implicated in cancer cell invasion and metastasis [4]. In the course of EMT, cells gradually trade key epithelial characteristics such as apical-basal polarity and tight cell-cell and cell-matrix contacts for mesenchymal features, including fibroblast-like morphology, front-rear polarity, increased motility, and enhanced invasiveness. EMT was also reported to confer increased drug resistance and superior tumor-initiation capacity or stemness features to cancer cells [4–6], although this may not apply to all tumor entities [7–9]. Cancer cells can be induced to undergo EMT in response to extrinsic and intrinsic stimuli, for example from the Wnt, Notch, mitogen-activated protein kinase (MAPK), and TGFβ signaling pathways [4]. Irrespective of the upstream trigger, a central role in EMT processes is typically attributed to a small group of TFs from the Snail, Zeb, and Twist families [10]. These EMT-TFs are thought to orchestrate the transition from epithelial to mesenchymal states by extensive transcriptional reprogramming. Yet, recent studies suggest that cancer cells may not need to acquire a fully mesenchymal phenotype in order to attain maximum invasive and metastatic capacity. Rather, the ability of cancer cells to traverse only partway through EMT and to present with variable combinations of epithelial and mesenchymal properties appears to be most advantageous for metastasis [5, 11]. Although instances of partial EMT (pEMT) were captured in vitro and in vivo [6, 11–15], it is not clear to which extent these only represent snapshots along a continuum of intermediate states towards complete EMT (cEMT) [12, 15] or reflect endpoints of a diversity of tissue-specific and mechanistically distinct pEMT programs [13, 14].

Colorectal cancer (CRC) is one of the most frequent forms of cancer worldwide. Histological examination indicated that the predominant form of stromal infiltration in CRC is collective invasion with evidence for pEMT at the invasive front [3]. How this particular pattern of invasiveness arises is unknown. Work with genetically engineered mouse models and organoids suggested that the three most common tumor-promoting events in CRC, disruption of the *Apc* and *Trp53* tumor suppressor genes in conjunction with oncogenic mutations in *Kras*, suffice to induce invasion and metastatic disease [16–19], but it was not determined whether the experimental models recapitulated the particular type of invasion observed in CRC tissue specimens. Furthermore, there are contradictory results concerning the number and type of genetic changes needed to elicit invasion and metastasis [18, 20–23], and an invasion-stimulating effect of the tumor microenvironment (TME) cannot be excluded.

TGFβ ligands are prototypic examples for microenvironmental factors with the capacity to induce cancer cell invasion. However, the role of TGFβ signaling in CRC is controversial. TGFβ receptors and Smad4, a key TF in canonical TGFβ signaling, are frequently inactivated to boost experimental metastasis in animal models [18, 20–24], and it was proposed that TGFβ signaling acts on nonneoplastic stromal rather than cancer cells to promote metastases formation [25, 26]. Yet, the TGFβ pathway appears intact and functional in the majority of CRC specimens [27], and comprehensive transcriptome analyses provided evidence for active TGFβ signaling also in cancer cells [28, 29], including the consensus molecular subtype 4 (CMS4) of human CRC, which is the CMS with the poorest prognosis [29, 30]. Likewise, tumor cell-specific TGFβ pathway activity was documented in a metastatic mouse tumor model [16], and TGFβ signaling induced invasion and installed CMS4-like transcription in tumor cells from a model of the sessile serrated adenoma CRC subtype [31]. Even though TGFβ signaling typically induces cEMT and single-cell invasion [12, 14, 15, 32], it therefore appears nonetheless worthwhile to closely examine its potential impact on CRC cell collective invasion.

Here, we aimed to dissect the impact of tumor cell genetics and extrinsic factors on invasive behavior in intestinal tumorigenesis by employing genetically modified murine organoids. We report that disruption of *Apc* and *Smad4* together with expression of Kras^G12D^ and dominant-negative p53^R172H^ do not suffice to elicit cell-intrinsic invasiveness. However, concomitant oncogenic lesions in the Wnt, MAPK, and p53 signaling pathways primed organoids for TGFβ1-inducible collective invasion and a stable, transcription-dependent pEMT which was executed independently of Snail1 and Zeb1. Interestingly, TGFβ1-treated organoids acquired a gene expression pattern representative of CMS4 suggesting that an atypical TGFβ1 response and a novel pEMT variant may underlie human CRC collective invasion.

## Results

### Intestinal organoids do not gain cell-intrinsic invasiveness by oncogenic transformation

**Fig. 1:**
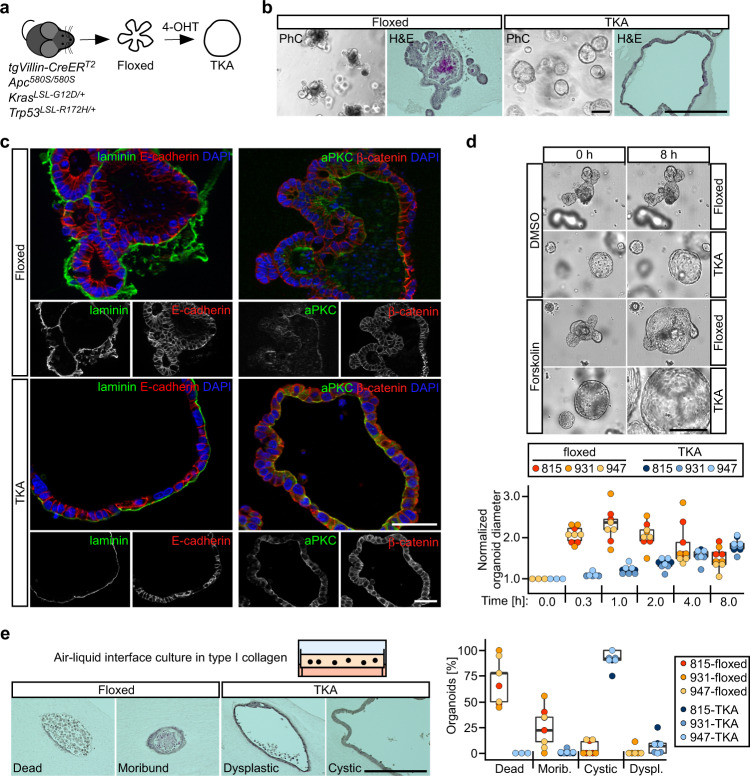
Oncogenically transformed intestinal organoids display no cell-intrinsic invasiveness in vitro. **a** Strategy for generating oncogenically transformed small intestinal and colonic organoids from genetically engineered mice carrying a *Villin-CreER*^*T2*^ transgene, two floxed *Apc* alleles, as well as heterozygous *Kras*^*LSL-G12D/+*^ and *Trp53*^*LSL-R172H/+*^ loci. Floxed stop cassettes (LSL) prevent expression of the mutant *Kras* and *Trp53* alleles. For recombination, organoids were treated with 4-hydroxy-tamoxifen (4-OHT), yielding TKA organoids. **b** Whole mounts and hematoxylin/eosin (H&E) stained sections of floxed and TKA organoids (line 815) cultured in 7 mg/ml Matrigel and visualized by phase contrast (PhC) or bright field microscopy. Scale bars: 200 µm. **c** Representative images of immunofluorescence staining of pan-laminin, E-cadherin, atypical protein kinase C (aPKC), and β-catenin in sections of floxed and TKA organoids (line 815) cultured in 7 mg/ml Matrigel. Nuclei were stained by DAPI; n > 3. Scale bars: 50 µm. **d** Top: Microscopy of floxed and TKA organoids (line 815) in 7 mg/ml Matrigel at the beginning (0 h) and the end (8 h) of forskolin and DMSO treatment. Scale bar: 200 µm. Bottom: Quantification of forskolin-induced organoid swelling. TKA organoids were exposed to DMSO or forskolin for the indicated time periods. Normalized changes in organoid diameter were calculated by first computing at each time point and for each organoid under consideration the increase in diameter relative to the corresponding value at *t* = 0 h, followed by normalization of forskolin-induced relative changes in diameter to those of DMSO-treated control samples. At least five organoids treated with DMSO or forskolin were analyzed per biological replicate and organoid line. **e** Top: setup for cultivating organoids in type I collagen at an air-liquid-interface and representative H&E stainings of organoid displaying different histological features (line 815). Scale bar: 200 µm. Bottom: Quantification of organoids following histological classification (morib.: moribund; dyspl.: dysplastic). Quantitative experiments in (**d**, **e**) were performed with three floxed/TKA organoid lines (815: *n* = 3; 931: *n* = 3; 947: *n* = 3). Dots represent results of independent biological replicates and dot color identifies the organoid lines.

To test whether hyperactive Wnt and MAPK signaling and impaired p53 activity induce invasiveness in a cell-autonomous fashion, we generated multiple small intestinal organoid lines from *Apc*^*580S/580S*^, *Kras*^*LSL-G12D/+*^, *Trp53*^*LSL-R172H/+*^, *tgVillinCreER*^*T2*^ mice (Fig. 1a, Supplementary Fig. 1). Organoids (hereafter termed floxed organoids) were treated with 4-hydroxy-tamoxifen (4-OHT) to obtain *Apc*-deficient organoids expressing oncogenic Kras^G12D^ and dominant-negative p53^R172H^ (hereafter called TKA organoids; Supplementary Fig. 1a–d). When compared to bud-forming floxed organoids, TKA organoids exhibited cystic growth [20, 21], remained viable in media without R-spondin-1, became EGF-independent and tolerated EGFR inhibition (Fig. 1b, Supplementary Fig. 1e, f). Furthermore, expression of Wnt target genes and intestinal stem cell markers increased in TKA organoids while that of differentiation markers decreased (Supplementary Fig. 1g). Notably, acquired growth factor independence and disturbed differentiation represent hallmarks of cancer cells. Yet, TKA organoids remained non-invasive. Like floxed organoids, they were surrounded by a continuous layer of laminin and showed apical localization of atypical protein kinase C (aPKC) (Fig. 1c). Forskolin-inducible swelling of organoids confirmed epithelial integrity [33], albeit floxed and TKA organoids displayed different swelling dynamics which might be caused by the differences in shape and elasticity of organoids (Fig. 1d). Thus, TKA organoids maintained apico-basolateral polarity, basement membrane integrity, and functional cell-cell junctions which are key characteristics of epithelial cell layers. To expose TKA organoids to an extracellular matrix (ECM) more representative of a desmoplastic TME [34], they were cultivated in an air-liquid interface setup with type I collagen. Under these conditions the majority of floxed organoids perished, whereas TKA organoids remained viable and occasionally showed evidence of dysplasia (Fig. 1e). However, TKA organoids did not infiltrate the type I collagen matrix. Thus, despite several lines of evidence for oncogenic transformation, TKA organoids did not display cell-intrinsic invasiveness arguing that invasive behavior observed in vivo might be triggered by cell non-autonomous mechanisms.

### TGFβ1 triggers collective invasion of TKA organoids and ECM remodeling

**Fig. 2:**
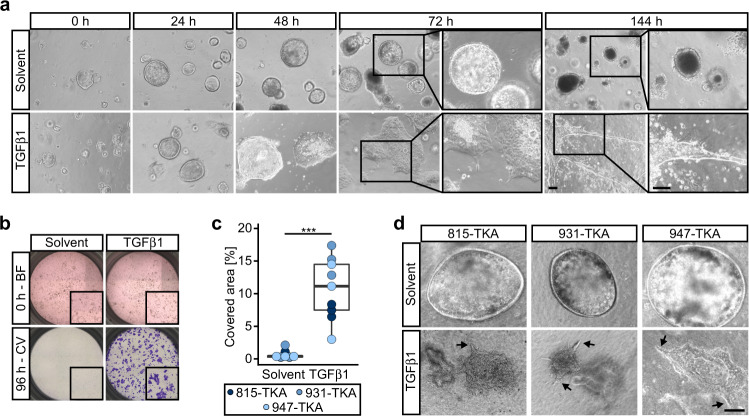
TGFβ1 triggers collective invasion of TKA organoids. **a** Morphological appearance of TKA organoids (line 815) cultured in 3 mg/ml Matrigel and treated with solvent or TGFβ1 for the indicated time periods. Boxed areas are shown at higher magnification on the right. Similar TGFβ1-induced morphological changes were observed with TKA organoid lines from five different founder mice. Scale bars: 100 µm. **b** Boyden chamber invasion assays with TKA organoids (line 931) seeded in 3 mg/ml Matrigel. Top: bright field (BF) images taken at 0 h of TGFβ1 and solvent treatment. Inserts show magnified views of the upper chambers. Bottom: crystal violet (CV) staining of invaded cells after 96 h of treatment. Inserts show magnified views of the bottom faces of the Boyden chambers. **c** Quantification of invasion experiments as shown in (**b**) performed with TKA organoid lines 815 (*n* = 3), 931 (*n* = 3), and 947 (n = 3). Each dot represents the result of a single invasion assay while dot color identifies the organoid lines. ***p = 0.0004; Mann–Whitney *U* test. **d** Phase contrast microscopy of TKA organoids cultured in type I collagen in presence of solvent or TGFβ1. Arrows point at sites of collective invasion. Independent experiments were performed in three different organoid lines (815: *n* = 3; 931: *n* = 3; 947: *n* = 3). Scale bar: 100 µm.

TGFβ1 is a prototypical TME signal and prime inducer of EMT. Because the vast majority of human metastatic CRCs possess an intact TGFβ signaling machinery (Supplementary Fig. 2a), we investigated the consequences of treating organoids with TGFβ1. Whereas floxed organoids died when exposed to TGFβ1, TKA organoids were resistant to TGFβ1-induced cell death (Supplementary Fig. 2b–d), probably due to expression of oncogenic Kras^G12D^ [35]. Cleaved caspase-3, indicative of ongoing apoptosis, was detectable only in cells that had been shed into the lumen of TKA organoids (Supplementary Fig. 2c, d). Instead of inducing apoptosis, TGFβ1 triggered massive morphological changes of TKA organoids which progressively lost their cystic shape, flattened, and extended multicellular protrusions, culminating in the formation of large, cohesive cell sheets and extended strands of cells infiltrating the surrounding Matrigel (Fig. 2a; Supplementary movie 1). Boyden chamber assays confirmed acquired invasiveness of TGFβ1-stimulated TKA organoids (Fig. 2b, c). TKA organoids embedded in a type I collagen matrix and exposed to TGFβ1 also remained viable, changed their shape, and became invasive (Fig. 2d). Moreover, colon-derived TKA organoids responded to TGFβ1 treatment with highly similar morphological changes and also displayed a collective mode of invasion (Supplementary Fig. 2e–g).

**Fig. 3:**
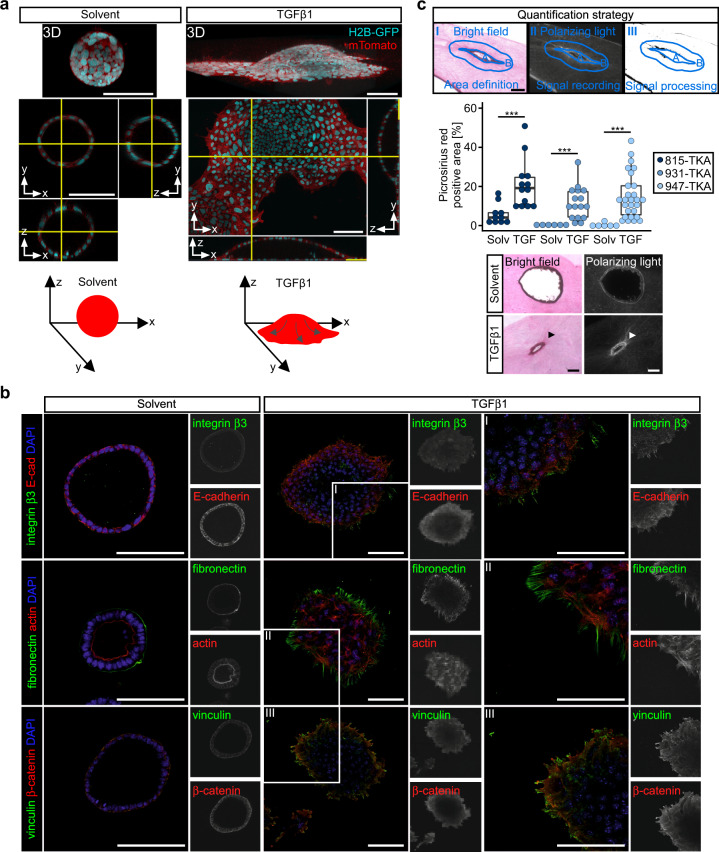
TGFβ1-stimulated TKA organoids interact with and remodel the ECM. **a** TKA organoids (line 815) expressing nuclear H2B-GFP and membrane-bound mTomato were seeded in 3 mg/ml Matrigel, treated with solvent or TGFβ1 for 48 h, and subjected to live imaging using confocal microscopy. Displayed are 3D reconstructions and orthogonal views of organoid cross-sections (yellow lines: positions of the cross-sections along the *x*-, *y*- and *z*-axes). Scale bars: 100 µm (*n* = 3). Bottom: schematic representations of TKA organoid morphologies. Arrows: proposed streaming of cells. **b** Whole-mount immunofluorescence staining and confocal microscopy of TKA organoids (line 815) seeded in 3 mg/ml Matrigel and treated with solvent or TGFβ1 for 72 h. Organoids were stained with antibodies against the indicated antigens. Actin was visualized by phalloidin staining. Nuclei were labeled using DAPI. Boxed areas are shown at higher magnification on the right. Pictures are representative for results obtained with three TKA organoid lines (815: *n* = 1; 931: *n* = 1; 947: *n* = 1). Scale bars: 100 µm. **c** Top: strategy for quantification of picrosirius red staining: (I) organoids were visualized by bright field illumination and the outline of the organoids (A) and a surrounding area (B) with an approximate width of 65 µm were marked. (II) Collagen was stained by picrosirius red and larger or parallel collagen bundles were visualized by polarizing light and signal intensities across the entire image were recorded. (III) For the final quantifications, only signals within area B and exceeding a defined threshold were considered. To allow comparison of organoids with different sizes, signal intensities were normalized to the size of area B. Middle: quantifications of picrosirius red-stained TKA organoids cultured in type I collagen and treated with solvent (solv) or TGFβ1 (TGF) for 96 h. Colored dots represent individual measurements from at least two independent experiments. Mann–Whitney *U* test. TKA organoid line 815: ***p = 0.0007 (solvent: *n* = 10, TGFβ1: *n* = 13); TKA organoid line 931: ***p = 0.0005 (solvent: *n* = 6, TGFβ1: n = 16); TKA organoid line 947: ***p = 0.0002 (solvent: *n* = 6, TGFβ1: *n* = 27). Scale bar: 100 µm. Bottom: exemplary pictures of TKA organoids (line 815) treated with solvent or TGFβ1.

Fluorescent labeling and confocal microscopy revealed that TGFβ1-treated TKA organoids adopted a dome-like structure with a central lumen and a broad circumferential rim of cells, which coherently expanded outwards (Fig. 3a). Invading cell sheets formed only at the organoid base close to the surface of the cell culture plates. Strikingly, single-cell delamination and invasion were not observed. Cells at the invasive organoid perimeter produced spike-like fibers of fibronectin (involved in ECM assembly [36]), showed punctiform staining of the integrin β3 subunit (involved in adhesion to fibronectin [37]), and of vinculin (marking focal adhesions [38]) (Fig. 3b; Supplementary Fig. 3). The observed topological restriction of invasion and fibronectin spike formation contrasts with the nuclear localization of Smad2/3 and, hence, active TGFβ signaling, and expression of fibronectin also in organoid cells constituting the dome structure (Supplementary Fig. 4). This suggests that invasive behavior aside from TGFβ pathway activation additionally depends on a permissive microenvironment. Indeed, to allow uniform TGFβ1-induced phenotypic switching in organoid cultures we had to lower the Matrigel concentration. Conversely, organoid cells contracted the surrounding ECM while infiltrating: TGFβ1-treated TKA organoids grown in a type I collagen matrix promoted formation of larger and more parallel aligned collagen bundles which could be visualized by picrosirius red staining (Fig. 3c). Altogether, TGFβ1 turned out to promote collective invasion of TKA organoids which appears to involve reciprocal organoid/ECM interactions and ECM remodeling.

### Canonical TGFβ signaling induces pEMT in TKA organoids

**Fig. 4:**
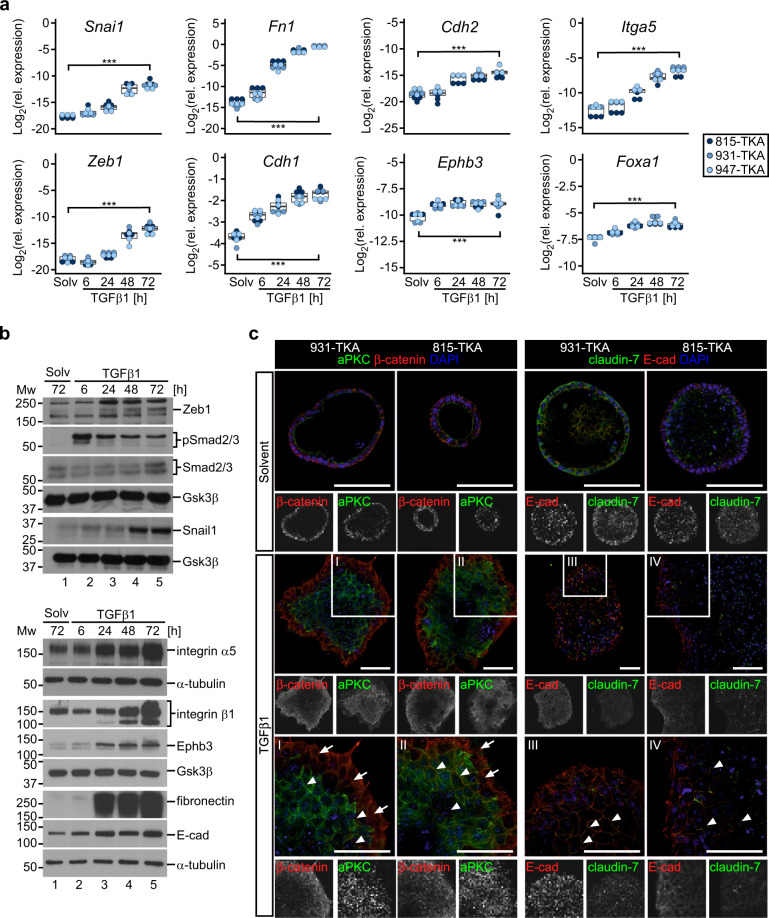
TGFβ1 induces a pEMT in TKA organoids. **a** Time-resolved gene expression analyses of TKA organoids seeded in 3 mg/ml Matrigel and treated with solvent (solv) or TGFβ1 for the indicated time periods. RNA levels of EMT-TFs and EMT-associated genes were quantified by qRT-PCR and normalized to transcript levels of *Eef1a1*. Each dot represents the result of a single measurement while dot color identifies the organoid lines. Three independent biological replicates were performed for each organoid line (815: *n* = 3; 931: *n* = 3; 947: *n* = 3). ***p < 0.001; statistical significance was analyzed using a linear regression model combined with Bonferroni correction for multiple comparisons. Exact *p*-values are provided in Supplementary Table 7. **b** Western blot analyses of phospho-Smad2/3 (pSmad2/3), total Smad2/3, EMT-TFs, and EMT-associated genes in TKA organoids (line 815) treated as in (**a**). Gsk3β and α-tubulin were included as loading control. Molecular weights of size standards are given in kDa. Results are representative for experiments performed with three TKA organoid lines (815: *n* = 2; 931: *n* = 2; 947: *n* = 2); E-cad: E-cadherin. **c** Whole-mount immunofluorescence staining and confocal microscopy of TKA organoid lines 815 and 931 seeded in 3 mg/ml Matrigel and treated with solvent or TGFβ1 for 72 h. Organoids were stained for E-cadherin, claudin-7, β-catenin, and atypical protein kinase C (aPKC). Arrowheads indicate membranous E-cadherin, claudin-7, and β-catenin in the central part of the TGFβ1-treated organoid. Arrows highlight reduced staining of aPKC in the peripheral region of TGFβ1-stimulated organoids. Boxed areas are shown at higher magnification below. Reduced staining intensities in some central regions of organoids might be caused by technical issues related to whole-mount staining and confocal microscopy of organoids. Similar results were obtained with three different organoid lines (815: *n* = 1, 931: *n* = 1, 947: *n* = 1). Scale bars: 100 µm.

Classically, TGFβ signaling induces cEMT and single-cell invasion [12, 14, 15, 32]. To better understand TGFβ1-driven collective invasion of TKA organoids, we performed time-resolved gene expression analyses. TGFβ1 markedly induced expression of EMT-TFs (Snail1, Zeb1), mesenchymal markers (fibronectin/*Fn1*, N-cadherin/*Cdh2*) (Fig. 4a, b), and *Itga5*, which, like *Itgb1*, is known to be upregulated during EMT [39]. Significantly, integrin α5β1 is a fibronectin receptor [37] and promotes cancer cell migration and invasion [39–41]. Western blot detection of phosphorylated Smad2/3 additionally confirmed TGFβ pathway activation. Surprisingly, TGFβ1 treatment did not diminish but rather increased expression of epithelial markers (E-cadherin/*Cdh1*, Ephb3, Foxa1), (Fig. 4a, b). Colonic TKA organoids responded to TGFβ1 treatment in a virtually identical fashion, showing concomitant upregulation of mesenchymal and epithelial gene expression and elevated levels of EMT-related integrins (Supplementary Fig. 5a, b).

Aside from transcriptional repression, a change in intracellular localization of cell-cell adhesion molecules may also contribute to EMT [14]. However, the adherence junction proteins E-cadherin and β-catenin were retained at cell-cell borders in all parts of TGFβ1-treated TKA organoids (Fig. 4c, Supplementary Fig. 5c). Only cells at the invasive front showed some increase in cytoplasmic E-cadherin and β-catenin staining, delocalization of the tight junction protein claudin-7, and reduction of aPKC (Fig. 4c) arguing for somewhat graded phenotypic changes. Nonetheless, given that the mesenchymal marker fibronectin was expressed not only at the invasive organoid base but also in cells constituting the dome structure (see above, Supplementary Fig. 4c), we conclude that cells throughout TGFβ1-stimulated TKA organoids adopt a pEMT state distinguished by concurrent exhibition of epithelial and mesenchymal characteristics and largely maintained membranous E-cadherin and β-catenin.

**Fig. 5:**
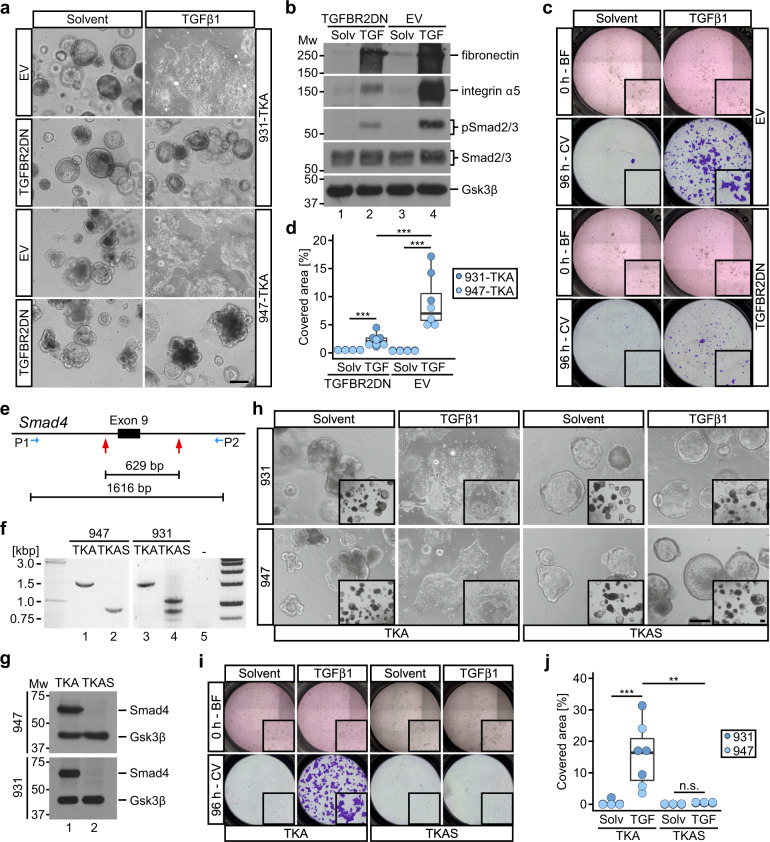
The TGFβ1 response in TKA organoids is mediated by canonical TGFβ-receptor/Smad signaling. **a** Whole-mount phase contrast microscopy of TKA organoids transduced with an empty vector (EV) or an expression vector for dominant negative TGFBR2 (TGFBR2DN), seeded in 3 mg/ml Matrigel, and treated with solvent or TGFβ1 for 72 h. Scale bar: 200 µm. **b** Western blot expression analysis of the indicated proteins in control (EV) and TGFBR2DN-expressing TKA organoids (line 947) treated as in (**a**). Gsk3β detection served as loading control. Molecular weights of size standards are given in kDa. Panels (**a**, **b**) show representative results of two independent biological replicates for TKA organoid lines 931 (*n* = 2) and 947 (*n* = 2). **c** Exemplary Boyden chamber invasion assays with control (EV) and TGFBR2DN-expressing TKA organoids (line 931) seeded in 3 mg/ml Matrigel. Bright-field (BF) images were taken at 0 h of solvent and TGFβ1 treatment. Inserts: magnified views of the upper chambers. Invaded cells were visualized by crystal violet (CV) staining after 96 h of treatment. Inserts: magnified views of the bottom face of the Boyden chambers. **d** Quantification of invasion experiments as shown in (**c**). Dots represent results of independent biological replicates (line 931: *n* = 3; line 947: *n* = 4). Dot color identifies the organoid lines. ***p < 0.001; Mann–Whitney *U* test. **e** Scheme of the *Smad4* locus showing sgRNA target positions (red arrows) flanking exon 9 (black box) and the location of PCR primers used for genotyping. The distance between the sgRNA targets and the length of the PCR amplicon in *Smad4* wt organoids are given in base pairs (bp). **f** Results of genotyping PCRs with genomic DNA from *Smad4* wt TKA and *Smad4* mutant TKAS organoid lines as indicated. Sizes of DNA standards are given in kilobase pairs (kbp). **g** Western blot expression analyses for Smad4 in TKA and TKAS organoids. Gsk3β detection served as loading control (*n* = 3). Molecular weights of size standards are given in kDa. **h** Whole-mount phase contrast microscopy of TKA and TKAS organoid lines seeded in 3 mg/ml Matrigel and treated with solvent or TGFβ1 for 72 h. Inserts display a larger field of view at lower magnification. Scale bars: 200 µm. **i** Boyden chamber invasion assays performed with TKA and TKAS organoids (line 931) as in (**c**). **j** Quantification of invasion experiments as shown in (**i**) performed with TKA and TKAS organoid lines 931 (*n* = 4) and 947 (*n* = 3). Dots represent results of individual experiments. Dot color identifies the organoid lines. ***p = 0.0006, **p = 0.0012, n.s. not significant (*p* = 0.39); Mann–Whitney *U* test. For (**d** and **j**), exact *p*-values are provided in Supplementary Table 7.

The unexpected induction of pEMT that did not progress to cEMT, prompted us to investigate the underlying signal transduction mechanisms. Overexpression of constitutively active TGF-β receptor type 1 (TGFBR1CA) [42] fully recapitulated TGFβ1-induced morphological changes and invasive behavior of TKA organoids (Supplementary Fig. 6a–c). Conversely, expressing dominant-negative TGF-β receptor type 2 (TGFBR2DN) [43] and pharmacologically inhibiting TGFBR1 blocked TGFβ1-induced phenotypic alterations (Fig. 5a–d, Supplementary Fig. 6d). Altogether, this argues that the TGFβ1 response of TKA organoids is mediated by genuine TGFβ receptor activity.

TGFβ receptors can signal through canonical, Smad-dependent mechanisms but also engage non-canonical signaling pathways, which in the context of EMT may include activation of MAPK cascades and PI3 kinase (PI3K)/AKT signaling [44]. To examine whether MAPK and PI3K activity contributed to the TGFβ1-induced pEMT of TKA organoids, we performed combinatorial treatments with TGFβ1, the MEK1/2 inhibitor trametinib, and the PI3K inhibitor buparlisib. However, combined TGFβ1/trametinib treatment led to the demise of the TKA organoids (Supplementary Fig. 7), presumably because TKA organoids lost Kras^G12D^-mediated protection against TGFβ1-induced cell death upon downstream inhibition of MAPK signaling. Further, PI3K inhibition impaired viability of TKA organoids regardless of TGFβ1 treatment. Thus, it was not possible to establish whether MAPK and PI3K activity played an additional role in the pEMT of TKA organoids as components of non-canonical TGFβ1 signaling pathways. A likely explanation for this could be that MAPK and PI3K activities are not exclusively involved in the TGFβ1 pathway but are embedded in multiple different signal transduction cascades and cellular processes.

While attempts to assess the role of MAPK and PI3K activity in TGFβ1-mediated pEMT and collective invasion of TKA organoids produced inconclusive results, targeting canonical, Smad-dependent TGFβ signaling proved informative. Thus, knockout of *Smad4*, yielding quadruple mutant TKAS organoids (Fig. 5e–g), completely abrogated the TGFβ1 response (Fig. 5h–j, Supplementary Fig. 8). However, unlike one might have expected based on in vivo studies [18, 20, 21, 24], knockout of *Smad4* did not promote invasiveness of TKAS organoids per se. In contrast to the deleterious consequences following inactivation of *Smad4*, *Smad2*-deficiency had no discernable effects on TGFβ1-induced morphological changes, collective invasion, and transcriptional responses of TKA organoids (Supplementary Figs. 9 and 10). On the other hand, *Smad3*-deficiency dampened the TGFβ1 response of TKA organoids, albeit it did not entirely abolish it (Supplementary Figs. 9 and 10). The differential impact on the TGFβ1 response of TKA organoids resulting from inactivation of *Smad2*, *Smad3*, and *Smad4* is consistent with the role of Smad4 as common binding partner of Smad2 and Smad3 [44], and both redundant and non-redundant functions of Smad2 and Smad3 in TGFβ signaling [44, 45]. Precisely which aspects of TGFβ1-induced pEMT and collective invasion of TKA organoids are controlled by the different Smad proteins and other interacting TFs needs to be determined in future experiments. Irrespective of this, we conclude that the observed pEMT of TKA organoids is conferred by receptor-mediated, canonical TGFβ signaling.

### Transcriptomes of TGFβ1-treated TKA organoids resemble CMS4 of human CRC

**Fig. 6:**
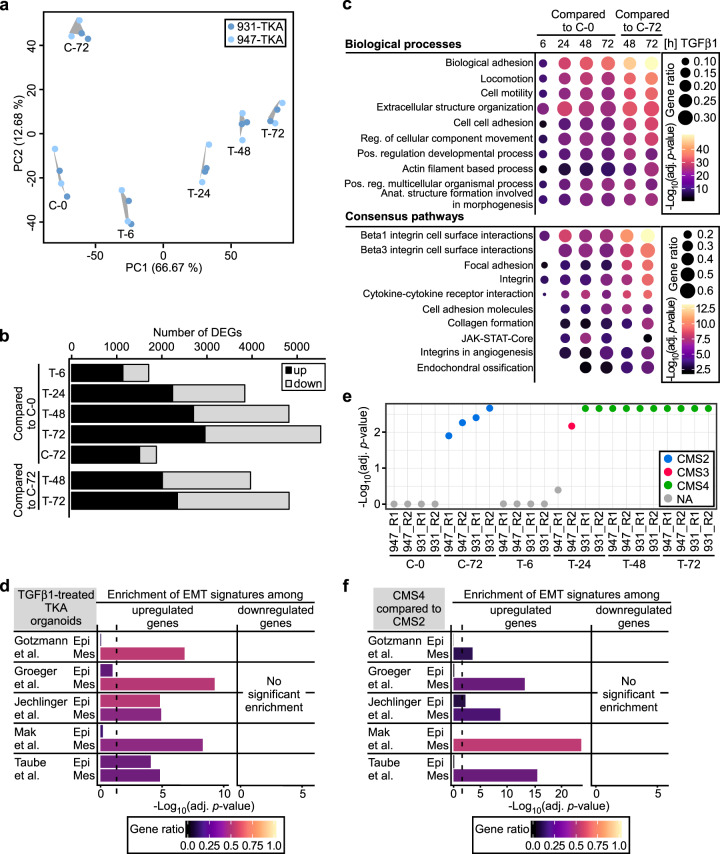
TGFβ1-induced global transcriptional deregulation features a pEMT in TKA organoids. **a** Principal component analysis (PCA) of RNA-seq data from TKA organoids seeded in 3 mg/ml Matrigel and stimulated with TGFβ1 for up to 72 h as indicated, or harvested at the onset of the experiment (C-0) and after 72 h of cultivation with solvent (C-72) to account for culture-dependent effects. Results are based on two independent biological replicates for organoid lines 931 (*n* = 2) and 947 (*n* = 2). **b** Numbers of differentially expressed genes (DEGs) were determined by performing pairwise comparisons of transcriptomes from TKA organoids treated as in (**a**). Black and gray segments of the bars: up- and downregulated genes, respectively. **c** Functional enrichment analysis of genes upregulated upon TGFβ1 treatment (adjusted *p* value <0.01, log_2_(FC) > 1). The top ten GO terms from the indicated categories are listed. Dot size: ratio of upregulated genes compared to all genes within a set. Dot color: -log_10_(adjusted [adj.] *p* value) of the enrichments. **d** Exclusive enrichment of mesenchymal components of published EMT signatures among genes significantly upregulated in TGFβ1-treated TKA organoids. The analyses were conducted for DEGs from TKA organoids treated with TGFβ1 for 72 h compared to cultivation for 72 h in solvent. Published EMT signatures were split into subsets comprising epithelial (Epi) and mesenchymal genes (Mes) and processed separately. The color encodes the gene ratio. The length of the bars depicts the -log_10_ of the adj. *p*-values. Dotted line: adj. *p* value = 0.05. **e** The independent biological replicates (R1, R2) of the transcriptomes as described in (**a**, **b**) were assessed for resemblance to the four consensus molecular subtypes (CMS) of CRC. Non-classification of C-0 and T-6 samples may result from mechanical disruption and reseeding of organoids which likely erased any typifying gene expression. Colored dots and their positions depict the CMS type and the adj. *p*-value, respectively. NA: no significant classification possible. **f** Functional enrichment analyses of EMT sub-signatures as described in (**d**) among genes differentially expressed in colon cancers classified as CMS4 and CMS2. The color encodes the gene ratio. The length of the bars depicts the -log_10_ of the adj. *p*-values. Dotted line: adj. *p* value = 0.05.

To comprehensively characterize TGFβ1-induced pEMT, we performed time-resolved transcriptome analysis by RNA sequencing (RNA-seq). Principal component (PC) analysis revealed high concordance between independent biological replicates from two organoid lines (Fig. 6a). A clear separation of control and TGFβ1-stimulated samples occurred along PC1, which accounts for most of the variances and likely reflects TGFβ1-induced gene expression changes over time. An additional segregation along PC2 might be attributable to organoid maturation (Fig. 6a). TGFβ1 caused extensive transcriptome changes, eventually comprising 2349 upregulated genes and 2471 downregulated genes after 72 h of stimulation (Fig. 6b, Supplementary Table 1; adjusted *p* value <0.01, │log_2_(fold change [FC])│ > 1). Functional enrichment analyses of gene sets revealed gene ontology (GO) terms related to cell-cell adhesion, locomotion, extracellular structure organization, integrins, focal adhesion, and collagen formation as significantly enriched among genes upregulated by TGFβ1 (Fig. 6c). In agreement with this and with the observed changes in invasiveness, ECM deposition, and reorganization, the RNA-seq data showed TGFβ1-mediated elevated expression of ECM components, receptors, and remodeling enzymes (Supplementary Table 2). Genes downregulated by TGFβ1 were enriched for GO terms connected to RNA and protein metabolic processes, DNA replication, and cell cycle (Supplementary Fig. 11a). Indeed, compared to controls, TGFβ1-treated TKA organoids contained only few Ki67-positive cells which were concentrated in the organoid center (Supplementary Fig. 11b). Accordingly, reduced proliferation may represent a common feature of pEMT and cEMT [46].

Additionally, we related our transcriptome data to previously defined EMT gene expression signatures [47–51]. Published data sets were split into epithelial and mesenchymal components, and enrichment of sub-signatures among the TGFβ1 up- and downregulated genes in TKA organoids was examined. Notably, TGFβ1-upregulated genes were significantly enriched for all mesenchymal and even two epithelial sub-signatures (Fig. 6d), whereas TGFβ1-downregulated genes showed no enrichment at all, confirming on a larger scale that TGFβ1-stimulated TKA organoids chiefly retain epithelial gene expression while concomitantly acquiring a mesenchymal transcriptional profile.

Human colorectal tumors can be classified into four subgroups CMS1-4 with distinctive transcriptomic features [29, 30]. To assess whether TKA organoids could be allocated to any of these, all RNA-seq data sets from control and TGFβ1-treated TKA organoids were compared to the CMS transcriptional profiles. Although control organoids (C-0) and organoids treated with TGFβ1 for 6 h could not be classified (Fig. 6e), the C-72 control group possessed gene expression properties of CMS2 which agrees well with hyperactive Wnt signaling in *Apc*-deficient TKA organoids [30]. In contrast, past 24 h of TGFβ1 treatment, TKA organoids showed a uniform association with CMS4 which is defined as mesenchymal with signs of increased TGFβ signaling and EMT [30]. Notably, when we interrogated human colon cancer transcriptome data from The Cancer Genome Atlas (TCGA) with respect to enrichment of EMT sub-signatures, we found that genes upregulated in CMS4 compared to CMS2 were significantly enriched for all mesenchymal sub-signatures. Again, downregulated genes displayed no enrichment for any sub-signature (Fig. 6f). This further highlights the similarity between transcriptomes of TGFβ1-treated TKA organoids and human CMS4 tumors, and additionally hints that CMS4 tumors likewise exhibit a pEMT.

### TGFβ1-induced pEMT represses intestinal stem cell signature gene expression and abrogates self-renewal capacity of TKA organoids

EMT processes and in particular pEMT states were repeatedly linked to the acquisition of stem cell properties and tumor-initiation capacity [4–6, 52, 53], although this may depend upon the tumor entities under investigation [7–9]. In CRC, several markers for cancer stem cells were identified [54, 55]. Furthermore, ISCs with a well-defined gene expression signature [56] are considered to be the cells of origin in intestinal carcinogenesis [57]. To test for a potential influence of TGFβ1-mediated pEMT on stemness characteristics of TKA organoids, we therefore analyzed TGFβ1-induced changes in the expression of the ISC signature and of *Cd44*, *Cd133*, *Aldh1a1*, *Epcam*, *Pou5f1 (Oct4)*, *Nanog*, and *Sox2* which represent the murine orthologues of human CRC stem cell markers [54, 55]. Functional enrichment analyses indicated an adverse effect of TGFβ1 on the expression of the ISC signature (Supplementary Fig. 12a). This was confirmed for the ISC markers *Lgr5* and *Ascl2* by qRT-PCR (Supplementary Fig. 12b). While no expression of *Sox2* and *Nanog* could be detected in unstimulated and TGFβ1-stimulated TKA organoids, *Pou5f1*, *Epcam*, *Cd133*, and *Cd44* were upregulated in response to TGFβ1 (Supplementary Fig. 12c, d). *Aldh1a1* expression on the other hand decreased. Thus, TGFβ1 prompted heterogeneous and non-uniform changes in the expression of ISC signature genes and CRC stem cell markers. Therefore, to directly assess the impact of TGFβ1 on stemness features of TKA organoids, we tested their self-renewal capacity under pEMT conditions. For this, TKA organoids were pretreated with TGFβ1 for 72 h, dissociated into single cells, and replated at different seeding densities. Compared to control conditions, TGFβ1 treatment completely abrogated organoid-forming capacity and, hence, self-renewal of TKA organoids (Supplementary Fig. 12e).

The *Itgav* gene codes for an integrin subunit listed among CRC stem cell markers [CD51; [55]], and was recently claimed to induce pEMT in lung cancer synergistically with TGFβ signaling [58]. Notably, *Itgav* is expressed in TKA organoids and is strongly upregulated upon exposure to TGFβ1 (Supplementary Figs. 13 and 14). This led us to investigate how inactivation of *Itgav* affected TKA organoids and their response to TGFβ1. However, a reduction in expression and functional impairment of integrin αV by deleting parts of its ligand-binding domain [59] had no detectable impact on the proliferation of TKA organoids under standard growth conditions and did not impair TGFβ1-induced pEMT and collective invasion (Supplementary Figs. 13 and 14). Of note, the ability of integrin αV subunit-containing integrins to promote TGFβ signaling through activation of ECM-sequestered latent TGFβ1 does not compromise these results because the experiments involved the exogenous administration of the active form of TGFβ1 which does not depend on integrin αV function.

Altogether, the analyses of marker gene expression and functional assessment of clonogenicity and self-renewal capacity indicate that TGFβ1-induced pEMT does not promote stemness of TKA organoids which is consistent with our previous observations in CRC cell lines [8, 9]. Furthermore, among the marker genes analyzed, TGFβ1-mediated downregulation of the ISC gene expression signature provides the best correlate with the observed loss of self-renewal capacity.

### TGFβ1-induced pEMT occurs independently from EMT master regulators

**Fig. 7:**
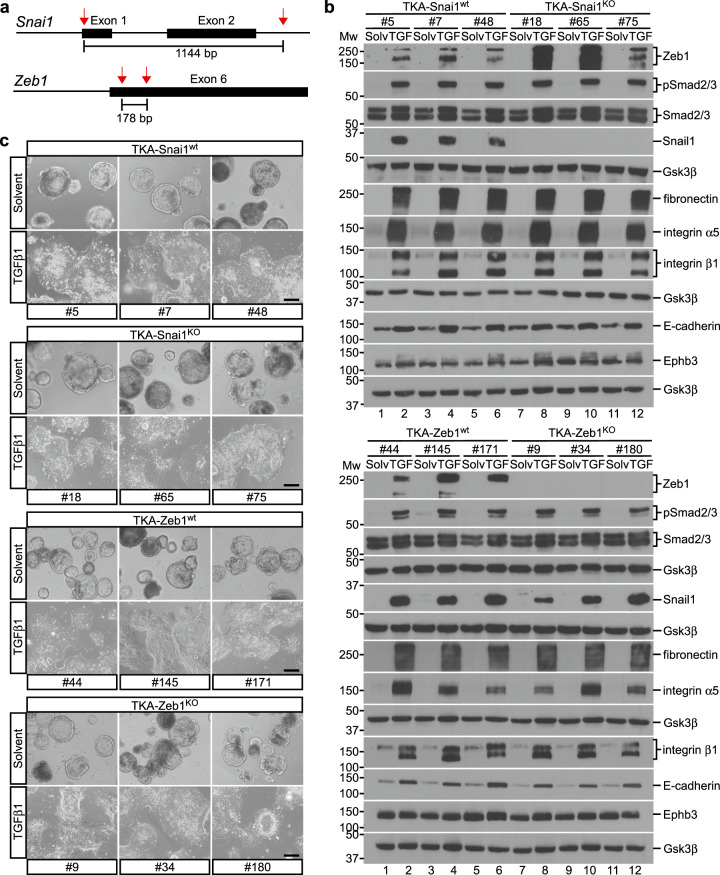
TGFβ1-induced collective invasion occurs independently from the EMT transcription factors Snail1 and Zeb1. **a** Schematics of the *Snai1* and *Zeb1* loci showing sgRNA target positions (red arrows) and relevant exons (black boxes). The size of the expected deletions is given in base pairs (bp). **b** Western blot expression analyses of the proteins indicated in TKA-Snai1^wt^, TKA-Snai1^KO^, TKA-Zeb1^wt^, and TKA-Zeb1^KO^ organoids derived from line 815 seeded in 3 mg/ml Matrigel and treated with solvent (solv) or TGFβ1 (TGF) for 72 h. Gsk3β detection served as loading control (*n* = 3). Molecular weights of size standards are given in kDa. **c** Morphological appearance of TKA-Snai1^wt^ (#5, #7, #48), TKA-Snai1^KO^ (#18, #65, #75), TKA-Zeb1^wt^ (#44, #145, #171), and TKA-Zeb1^KO^ (#9, #34, #180) organoids derived from line 815 cultured in 3 mg/ml Matrigel and treated with solvent or TGFβ1 for 72 h. Scale bars: 200 µm.

Snail, Zeb, and Twist TF family members are thought to fulfill key functions in EMT processes [4, 60]. Among these, *Snai1* and *Zeb1* were the only genes that were consistently upregulated in all TGFβ1-treated TKA organoid lines and reached by far the highest expression levels (Supplementary Fig. 15). Therefore, we examined their roles in TGFβ1-inducible pEMT. Both genes were targeted applying a dual single guide RNA (sgRNA)-mediated deletion strategy, and multiple, clonally derived wildtype (TKA-Snai1^wt^, TKA-Zeb1^wt^) and knockout (TKA-Snai1^KO^, TKA-Zeb1^KO^) organoid lines were obtained (Fig. 7a). Importantly, Snail1 and Zeb1 deficiencies did not impair TGFβ pathway activity as demonstrated by unabated Smad2/3 phosphorylation (Fig. 7b). Despite the complete absence of Snail1 and Zeb1, TGFβ1-regulated expression of EMT-associated genes, as well as TGFβ1-induced morphological conversion and invasiveness were unaffected (Fig. 7b; Supplementary Fig. 15). To complement these loss-of-function experiments, we generated TKA organoids which doxycycline (Dox)-inducibly expressed Snail1 and ZEB1 (Supplementary Fig. 16). While ZEB1 overexpression had no discernible impact on organoid morphology and invasiveness (Supplementary Fig. 16), TKA organoids expressing Snail1 lost their cystic shape, occasionally infiltrated the surrounding Matrigel, and exhibited some invasiveness in Boyden chamber assays, albeit much less pronounced compared to TGFβ1 stimulation (Supplementary Fig. 16c–e). Neither ZEB1 nor Snail1 overexpression reproduced TGFβ1-mediated gene expression changes (Supplementary Fig. 16f). All in all, overexpression of Snail1 and ZEB1 did not mimic the TGFβ1 response of TKA organoids. Thus, classical EMT-TFs are neither sufficient nor required for TGFβ1-mediated pEMT and collective invasion.

## Discussion

To decipher the molecular and cellular basics of tumor invasion and metastasis poses a persistent challenge, and it is not clear to which extent these processes are driven by genetic changes in cancer cells and by extrinsic factors from their surroundings. Here we used small intestinal and colonic organoids in a naïve, wildtype state to inflict oncogenic lesions only in vitro. This allowed us to assess the cell-autonomous impact of oncogenic transformation on epithelial integrity and invasiveness without prior exposure to confounding influence from non-tumor tissue as e. g. in animal models. Thereby, we found that the mutation of *Apc*, *Kras*, *Trp53*, and *Smad4* was insufficient to elicit cell-intrinsic invasive behavior in organoids. This contrasts with results from genetically engineered mouse models and organoid transplantation experiments where similar combinations of mutations led to the formation of invasive (since metastasizing) tumors [16–24]. This difference in invasiveness in vitro and in vivo strongly argues that stimuli from the microenvironment were responsible for triggering cell invasion in the in vivo settings. In this sense, oncogenic transformation constitutes an obligatory prerequisite, but only conditions tumor cells to become responsive to external pro-invasive signals. The implication of cell non-autonomous mechanisms in metastasis is consistent with the genetic similarity between primary and secondary lesions, and the failure to identify dedicated metastasis driver and suppressor genes in CRC [61]. Furthermore, the critical importance of the TME for invasion and metastasis could readily explain inconsistent results concerning the mutational spectrum required for metastasis in autochthonous tumor models and upon heterotopic transplantations [16–24].

To test the idea that microenvironmental signals elicit invasiveness of TKA organoids, we used the TGFβ pathway as a proof-of-principle. TGFβ signaling was previously shown to promote intestinal cancer metastasis, albeit indirectly by acting on non-cancer cells [25, 26]. Although it is commonly pointed out that TGFβ pathway components are frequently mutated in colorectal tumors, and that disruption of TGFβ signaling promotes malignant progression in experimental models of intestinal cancer [18, 20–24] our results show that cancer cells themselves can be relevant targets of TGFβ signaling. In support of this, TGFβ receptors and SMAD genes are intact in more than 60% of human CRCs [27], and cancer cell-intrinsic TGFβ pathway activity is evident in poor prognosis colorectal tumors [28, 30]. Importantly, this also applies to CMS4 cancer cells after exclusion of stromal contamination [29].

Our findings reinforce the importance of the TME for tumor invasion [62, 63]. They further demonstrate that the TME can influence cancer cells in different, yet cooperating ways, first by providing an invasion-permissive ECM and second by supplying pro-invasive growth factors and cytokines. Nonetheless, TKA organoids did not appear to just follow environmental cues, but actively interacted with their surroundings. The capacity to deposit and remodel ECM components in the tumor stroma therefore may not be restricted to cancer-associated fibroblasts and other non-cancer cells [64, 65].

While TGFβ1-induced pEMT and promoted collective invasion, it also caused the loss of self-renewal capacity of TKA organoids. This finding contrasts with the widely held notion that EMT is accompanied by the acquisition of enhanced tumor-initiation potential and stemness properties of cancer cells [4–6, 11]. However, EMT processes may affect stemness features depending upon tumor entity [7–9, 66, 67] and the respective cells-of-origin which in CRC are fully epithelial ISCs or ISC-like cells [57]. In fact, the adverse effect of pEMT on organoid-forming ability is in line with the observed downregulation of ISC signature genes which we had also demonstrated for cEMT in human CRC cell lines [8, 9, 68], and with the reduction in proliferation which is commonly associated with EMT [8, 9, 46]. Our observations are also in agreement with current concepts suggesting that cancer cell plasticity and partial or complete reversal of EMT may be critical for metastatic colonization [4, 5].

TGFβ ligands are paradigmatic inducers of EMT through extensive transcriptional reprogramming which engages EMT-TFs and typically culminates in complete mesenchymal conversion [12, 14, 15, 32]. Therefore, the ability of canonical, Smad4-dependent TGFβ signaling to induce pEMT without progression to a fully mesenchymal state represents a novel finding. Additionally, pEMT in TKA organoids was accompanied by collective invasion which likewise is not commonly associated with TGFβ-induced EMT [4, 10, 14, 32]. Moreover, collective invasion and pEMT of TKA organoids were transcriptionally regulated whereby a mesenchymal gene expression program was implemented on top of a largely maintained epithelial program, including chiefly sustained membrane localization of E-cadherin and β-catenin. The hybrid epithelial/mesenchymal state of TGFβ1-treated TKA organoids therefore differs from previously described variants of pEMT which involved broadly downregulated epithelial gene expression or the abrogation of epithelial cell characteristics by transcription-independent internalization of cell adhesion molecules [11, 14]. Interestingly, the gene regulatory network which was activated by TGFβ1 in TKA organoids, seemingly differs from that operating in pEMT states in squamous cell carcinoma and breast cancer (Supplementary Fig. 17) [6, 11]. Accordingly, we believe that the TGFβ1 response of TKA organoids represents a new and distinct pEMT program.

We suspect that the observed switch from TGFβ1-induced cEMT to pEMT is a consequence of context-dependent differences in the functionality of EMT-TFs. This idea is supported by our overexpression experiments which revealed much restricted EMT-inducing capacities of the two factors. Impaired function of EMT-TFs could be related to peculiarities of intestinal cells concerning posttranslational modifications of Snail1 and Zeb1 and to the expression of transcriptional co-factors [60]. Above that, our results imply that EMT-TFs can be upregulated as passengers without functionally contributing to EMT processes. Conversely, lack of EMT-TF expression may not equal absence of EMT. This has implications for the use of Snail1 and Zeb1 as EMT markers, and for the interpretation of findings that refuted a role of EMT in metastasis based on knockout animals [69].

In agreement with their genetic constitution, TKA organoids exhibited a gene expression pattern resembling CMS2 which is dominated by Wnt pathway activity [30]. Upon treatment with TGFβ1, organoids adopted a transcriptional profile highly similar to the transcriptome of CMS4 cancers which are characterized by TGFβ pathway activation, mesenchymal features, high stromal content, and particularly poor prognosis [30]. Convertibility to CMS4, thus, may not be limited to the sessile serrated adenoma path of colorectal carcinogenesis [31]. Acquisition of CMS4 characteristics may only require a functional TGFβ pathway and could be determined primarily by microenvironmental influences. Notably, re-examination of gene expression patterns unearthed evidence for pEMT in human CMS4 samples, strengthening the significance of our findings for human cancer. Adding further to the pathophysiological relevance of our study, we note that collective invasion, which was triggered by TGFβ1 in TKA organoids, accurately reflects the predominant mode of stromal infiltration in most carcinomas.

Altogether, our study significantly expands the understanding of the mechanistic foundations of pEMT and its context-dependent, distinctive appearances. Thereby, it may point out potential therapeutic strategies to eventually interfere with the metastatic cascade.

## Materials and methods

### Organoid culture

Small intestinal and colonic organoids were established from 9–13 weeks old, male and female C57BL/6 N *Apc*^*580S/580S*^; *Kras*^*LSL-G12D/+*^; *Trp53*^*LSL-R172H/+*^; *tgVillin-CreER*^*T2*^ mice [70–73] as described [74], and labeled with the identifier of founder animals (male: #815, #947, #1041; female: #931, #978). Mice were handled in accordance with legal regulations at the Center for Experimental Models and Transgenic Service of the University of Freiburg Medical Center (project registration number: X-17/07 S). For recombination, organoids were treated with 0.5 µM 4-hydroxy-tamoxifen (4-OHT; #H7904, Sigma Aldrich, Taufkirchen, Germany) for up to 120 h. Recombination was verified by PCR with primers listed in Supplementary Table 3 and genomic DNA isolated with the ReliaPrep™ gDNA Tissue Miniprep System (#A2052, Promega, Fitchburg, Wisconsin, USA) and peqGOLD Tissue DNA Mini Kit (#12-3496-02, VWR, Bruchsal, Germany). Upon inactivation of *Apc*, R-spondin-1 was omitted from the culture media.

### Treatment of organoids with TGFβ1 and small molecule inhibitors

A detailed description of these procedures can be found in the Supplementary Information (SI). Briefly, organoids were mechanically disrupted and seeded 30-48 h before being treated with 5 ng/ml human TGFβ1, 10 µM SB431542, 0.8 µM gefitinib, 30 nM trametinib, or 3 µM buparlisib. Organoid viability was monitored by phase contrast microscopy and MTT staining.

### Assessment of epithelial integrity

To functionally assess epithelial integrity, organoids were seeded as described above and treated with 5 µM forskolin. Forskolin-induced organoid swelling was followed by time-lapse microscopy. Further details of these experiments are provided in the SI.

### Boyden chamber invasion assay, air-liquid interface culture, culture in type I collagen, assessment of organoid-forming capacity

Details of these assays are provided in the SI.

### Genome editing

*Itgav, Smad2, Smad3, Smad4*, *Snai1*, and *Zeb1* were inactivated by frame-shift-inducing exon deletions using two sgRNAs (Supplementary Table 3). Expression cassettes for sgRNAs were generated with the MuLE system (Supplementary Table 4). For *Smad4* editing, floxed organoids were transfected with the sgRNA expression plasmids and pCAG-Cas9-turbo-RFP. *Itgav*, *Smad2*, *Smad3*, *Snai1*, and *Zeb1* were inactivated by lentiviral transduction. Details of the transfection/transduction procedures and subsequent organoid processing are provided in the SI.

### Viral transduction

Lentiviral and retroviral particles were produced by co-transfecting HEK293T cells with viral vectors and packaging plasmids (Supplementary Table 4). Additional information about viral particle production and transduction is given in the SI.

### Picrosirius red staining

Ninety-six hours post solvent or TGFβ1 treatment, organoid cultures in type I collagen were fixed in 10% formalin, paraffin embedded, and sectioned into 40 µm slices. Picrosirius red staining was performed as described [75]. Imaging and quantification procedures are described in the SI.

### Immunofluorescence staining and microscopy

Paraffin sections and whole mounts of organoids were stained with primary and secondary antibodies (Supplementary Table 5) using protocols described in the SI. Images of stained sections were acquired using an Axio Observer.Z1 fluorescence microscope with an ApoTome2 equipment (Zeiss, Oberkochen, Germany). Whole mounts were imaged with a LSM 880 confocal microscope (Zeiss) with an Achroplan IR 40×/0.8 W objective. Additional information about fluorescence microscopy and procedures for live imaging of organoids expressing mTomato and H2B-GFP are presented in the SI.

### RNA and protein expression analyses

RNA isolation, cDNA synthesis, and conduction of qRT-PCRs using primers listed in Supplementary Table 6 are described in the SI. For transcriptome analyses, RNA was collected from TGFβ1-treated organoids after 6, 24, 48, and 72 h, and from solvent-treated controls after 0 and 72 h of cultivation and paired-end sequenced on an Illumina HiSeq4000 at the Genome and Proteome Core Facility of the German Cancer Research Center, Heidelberg, Germany. Details of the processing and analysis of RNA-seq data can be found in the SI. The procedures of protein extraction and Western blotting are provided in the SI.

### CMS classification

The R/Bioconductor package CMScaller was used to determine the CMS of a given sample [76]. For organoid transcriptome data, mouse genes were first mapped to their human orthologs using the getHomoGeneIDs function from the R/Bioconductor package GeneAnswers with the direct mapping method [77].

### Analysis of colon cancer data

Colon cancer RNA-seq data were downloaded from TCGA firehose (https://gdac.broadinstitute.org) and subjected to CMS classification to determine DEGs in CMS4 compared to CMS2 cancers. Details of the procedure are provided in the SI.

### Query of publicly available cancer genome data and ISMARA

Genetic alterations in the TGFβ pathway were determined in CRC samples [61] using cBioPortal [78, 79]. Motif activities were predicted by ISMARA [80]. Motif activity profiles for TFs related to different EMT states were extracted according to ref. [5].

### Statistics and software

Data were analyzed and visualized using GraphPad Prism 5 (GraphPad Software) and RStudio [81]. For time-resolved targeted gene expression studies, statistical analyses were done using a linear model allowing for simultaneous statistical testing for different time points. Normal distribution was assessed with the car package [82]. We applied the lm () function and defined the solvent control as intercept to estimate effect sizes and calculate *p*-values based on t-statistics. Analyses were completed by Bonferroni correction for multiple comparisons. When comparing two populations, statistical significance was assessed using the two-tailed Mann–Whitney *U* test with a confidence interval of 95%. Box plots were generated with ggplot2 [83] and display the median with the lower and upper quartile. Whiskers show 1.5 times the interquartile range. Final figures were assembled using Canvas X 2017 (Canvas GFX, Inc.).

## Supplementary information

Supplementary information Flum et al

Supplementary movie_1_Flum et al

Supplementary table 1_Flum et al

Supplementary table 2_Flum et al

Supplementary table 3_Flum et al

Supplementary table 4_Flum et al

Supplementary table 5_Flum et al

Supplementary table 6_Flum et al

Supplementary table 7_Flum et al

## Data Availability

The RNA-seq data were deposited in the Gene Expression Omnibus with the accession code GSE156553. All other data generated and backing the results and conclusions of this study are available from the corresponding author on reasonable request.

## Code availability

The code used will be made available upon request.

## References

[1] Valastyan S, Weinberg RA (2011). Tumor metastasis: molecular insights and evolving paradigms. Cell.

[2] Friedl P, Locker J, Sahai E, Segall JE (2012). Classifying collective cancer cell invasion. Nat Cell Biol.

[3] Bronsert P, Enderle-Ammour K, Bader M, Timme S, Kuehs M, Csanadi A (2014). Cancer cell invasion and EMT marker expression: a three-dimensional study of the human cancer-host interface. J Pathol.

[4] Dongre A, Weinberg RA (2019). New insights into the mechanisms of epithelial-mesenchymal transition and implications for cancer. Nat Rev Mol Cell Biol.

[5] Pastushenko I, Blanpain C (2019). EMT transition states during tumor progression and metastasis. Trends Cell Biol.

[6] Kröger C, Afeyan A, Mraz J, Eaton EN, Reinhardt F, Khodor YL (2019). Acquisition of a hybrid E/M state is essential for tumorigenicity of basal breast cancer cells. Proc Natl Acad Sci USA.

[7] Celià-Terrassa T, Meca-Cortés O, Mateo F, Martínez de Paz A, Rubio N, Arnal-Estapé A (2012). Epithelial-mesenchymal transition can suppress major attributes of human epithelial tumor-initiating cells. J Clin Investig.

[8] Beyes S, Andrieux G, Schrempp M, Aicher D, Wenzel J, Antón-García P (2019). Genome-wide mapping of DNA-binding sites identifies stemness-related genes as directly repressed targets of SNAIL1 in colorectal cancer cells. Oncogene.

[9] Rönsch K, Jägle S, Rose K, Seidl M, Baumgartner F, Freihen V (2015). SNAIL1 combines competitive displacement of ASCL2 and epigenetic mechanisms to rapidly silence the EPHB3 tumor suppressor in colorectal cancer. Mol Oncol.

[10] Lamouille S, Xu J, Derynck R (2014). Molecular mechanisms of epithelial-mesenchymal transition. Nat Rev Mol Cell Biol.

[11] Pastushenko I, Brisebarre A, Sifrim A, Fioramonti M, Revenco T, Boumahdi S (2018). Identification of the tumour transition states occurring during EMT. Nature.

[12] Zhang J, Tian X-J, Zhang H, Teng Y, Li R, Bai F (2014). TGF-β-induced epithelial-to-mesenchymal transition proceeds through stepwise activation of multiple feedback loops. Sci Signal.

[13] Puram SV, Tirosh I, Parikh AS, Patel AP, Yizhak K, Gillespie S (2017). Single-cell transcriptomic analysis of primary and metastatic tumor ecosystems in head and neck. Cancer Cell.

[14] Aiello NM, Maddipati R, Norgard RJ, Balli D, Li J, Yuan S (2018). EMT subtype influences epithelial plasticity and mode of cell migration. Dev Cell.

[15] McFaline-Figueroa JL, Hill AJ, Qiu X, Jackson D, Shendure J, Trapnell C (2019). A pooled single-cell genetic screen identifies regulatory checkpoints in the continuum of the epithelial-to-mesenchymal transition. Nat Genet.

[16] Boutin AT, Liao W-T, Wang M, Hwang SS, Karpinets TV, Cheung H (2017). Oncogenic Kras drives invasion and maintains metastases in colorectal cancer. Genes Dev.

[17] Roper J, Tammela T, Cetinbas NM, Akkad A, Roghanian A, Rickelt S (2017). In vivo genome editing and organoid transplantation models of colorectal cancer and metastasis. Nat Biotechnol.

[18] O’Rourke KP, Loizou E, Livshits G, Schatoff EM, Baslan T, Manchado E (2017). Transplantation of engineered organoids enables rapid generation of metastatic mouse models of colorectal cancer. Nat Biotechnol.

[19] Fumagalli A, Oost KC, Kester L, Morgner J, Bornes L, Bruens L (2020). Plasticity of Lgr5-negative cancer cells drives metastasis in colorectal cancer. Cell Stem Cell.

[20] Drost J, van Jaarsveld RH, Ponsioen B, Zimberlin C, van Boxtel R, Buijs A (2015). Sequential cancer mutations in cultured human intestinal stem cells. Nature.

[21] Matano M, Date S, Shimokawa M, Takano A, Fujii M, Ohta Y (2015). Modeling colorectal cancer using CRISPR-Cas9-mediated engineering of human intestinal organoids. Nat Med.

[22] Li X, Nadauld L, Ootani A, Corney DC, Pai RK, Gevaert O (2014). Oncogenic transformation of diverse gastrointestinal tissues in primary organoid culture. Nat Med.

[23] Sakai E, Nakayama M, Oshima H, Kouyama Y, Niida A, Fujii S (2018). Combined mutation of Apc, Kras, and Tgfbr2 effectively drives metastasis of intestinal cancer. Cancer Res.

[24] Fumagalli A, Drost J, Suijkerbuijk SJE, van Boxtel R, de Ligt J, Offerhaus GJ (2017). Genetic dissection of colorectal cancer progression by orthotopic transplantation of engineered cancer organoids. Proc Natl Acad Sci USA.

[25] Calon A, Espinet E, Palomo-Ponce S, Tauriello DVF, Iglesias M, Céspedes MV (2012). Dependency of colorectal cancer on a TGF-β-driven program in stromal cells for metastasis initiation. Cancer Cell.

[26] Tauriello DVF, Palomo-Ponce S, Stork D, Berenguer-Llergo A, Badia-Ramentol J, Iglesias M (2018). TGFβ drives immune evasion in genetically reconstituted colon cancer metastasis. Nature.

[27] Cancer Genome Atlas Network. Comprehensive molecular characterization of human colon and rectal cancer. Nature. 2012;487:330. https://www.nature.com/articles/nature11252.pdf. 10.1038/nature11252 PMC3401966 22810696

[28] Isella C, Brundu F, Bellomo SE, Galimi F, Zanella E, Porporato R (2017). Selective analysis of cancer-cell intrinsic transcriptional traits defines novel clinically relevant subtypes of colorectal cancer. Nat Commun.

[29] Linnekamp JF, van Hooff SR, Prasetyanti PR, Kandimalla R, Buikhuisen JY, Fessler E (2018). Consensus molecular subtypes of colorectal cancer are recapitulated in in vitro and in vivo models. Cell Death Differ.

[30] Guinney J, Dienstmann R, Wang X, de Reyniès A, Schlicker A, Soneson C (2015). The consensus molecular subtypes of colorectal cancer. Nat Med.

[31] Fessler E, Drost J, van Hooff SR, Linnekamp JF, Wang X, Jansen M (2016). TGFβ signaling directs serrated adenomas to the mesenchymal colorectal cancer subtype. EMBO Mol Med.

[32] Giampieri S, Manning C, Hooper S, Jones L, Hill CS, Sahai E (2009). Localized and reversible TGFbeta signalling switches breast cancer cells from cohesive to single cell motility. Nat Cell Biol.

[33] Dekkers JF, Wiegerinck CL, de Jonge HR, Bronsveld I, Janssens HM, de Winter-de Groot KM (2013). A functional CFTR assay using primary cystic fibrosis intestinal organoids. Nat Med.

[34] Nissen NI, Karsdal M, Willumsen N (2019). Collagens and cancer-associated fibroblasts in the reactive stroma and its relation to cancer biology. J Exp Clin Cancer Res.

[35] Wiener Z, Band AM, Kallio P, Högström J, Hyvönen V, Kaijalainen S (2014). Oncogenic mutations in intestinal adenomas regulate Bim-mediated apoptosis induced by TGF-β. Proc Natl Acad Sci USA.

[36] Singh P, Carraher C, Schwarzbauer JE (2010). Assembly of fibronectin extracellular matrix. Annu Rev Cell Dev Biol.

[37] Danen EHJ, Sonneveld P, Brakebusch C, Fassler R, Sonnenberg A (2002). The fibronectin-binding integrins alpha5beta1 and alphavbeta3 differentially modulate RhoA-GTP loading, organization of cell matrix adhesions, and fibronectin fibrillogenesis. J Cell Biol.

[38] Bays JL, DeMali KA (2017). Vinculin in cell-cell and cell-matrix adhesions. Cell Mol Life Sci.

[39] Maschler S, Wirl G, Spring H, Bredow DV, Sordat I, Beug H (2005). Tumor cell invasiveness correlates with changes in integrin expression and localization. Oncogene.

[40] Mise N, Savai R, Yu H, Schwarz J, Kaminski N, Eickelberg O (2012). Zyxin is a transforming growth factor-β (TGF-β)/Smad3 target gene that regulates lung cancer cell motility via integrin α5β1. J Biol Chem.

[41] Kuonen F, Surbeck I, Sarin KY, Dontenwill M, Rüegg C, Gilliet M (2018). TGFβ, fibronectin and integrin α5β1 promote invasion in basal cell carcinoma. J Invest Dermatol.

[42] Morén A, Imamura T, Miyazono K, Heldin C-H, Moustakas A (2005). Degradation of the tumor suppressor Smad4 by WW and HECT domain ubiquitin ligases. J Biol Chem.

[43] Siegel PM, Shu W, Cardiff RD, Muller WJ, Massagué J (2003). Transforming growth factor beta signaling impairs Neu-induced mammary tumorigenesis while promoting pulmonary metastasis. Proc Natl Acad Sci USA.

[44] Derynck R, Budi EH (2019). Specificity, versatility, and control of TGF-β family signaling. Sci Signal.

[45] Brown KA, Pietenpol JA, Moses HL (2007). A tale of two proteins: differential roles and regulation of Smad2 and Smad3 in TGF-beta signaling. J Cell Biochem.

[46] Vega S, Morales AV, Ocaña OH, Valdés F, Fabregat I, Nieto MA (2004). Snail blocks the cell cycle and confers resistance to cell death. Genes Dev.

[47] Jechlinger M, Grunert S, Tamir IH, Janda E, Lüdemann S, Waerner T (2003). Expression profiling of epithelial plasticity in tumor progression. Oncogene.

[48] Gotzmann J, Fischer ANM, Zojer M, Mikula M, Proell V, Huber H (2006). A crucial function of PDGF in TGF-beta-mediated cancer progression of hepatocytes. Oncogene.

[49] Taube JH, Herschkowitz JI, Komurov K, Zhou AY, Gupta S, Yang J (2010). Core epithelial-to-mesenchymal transition interactome gene-expression signature is associated with claudin-low and metaplastic breast cancer subtypes. Proc Natl Acad Sci USA.

[50] Gröger CJ, Grubinger M, Waldhör T, Vierlinger K, Mikulits W (2012). Meta-analysis of gene expression signatures defining the epithelial to mesenchymal transition during cancer progression. PLoS ONE.

[51] Mak MP, Tong P, Diao L, Cardnell RJ, Gibbons DL, William WN (2016). A patient-derived, pan-cancer EMT signature identifies global molecular alterations and immune target enrichment following epithelial-to-mesenchymal transition. Clin Cancer Res.

[52] Carstens JL, Yang S, Correa de Sampaio P, Zheng X, Barua S, McAndrews KM (2021). Stabilized epithelial phenotype of cancer cells in primary tumors leads to increased colonization of liver metastasis in pancreatic cancer. Cell Rep.

[53] Simeonov KP, Byrns CN, Clark ML, Norgard RJ, Martin B, Stanger BZ, et al. Single-cell lineage tracing of metastatic cancer reveals selection of hybrid EMT states. Cancer Cell. 2021;39:1150-1162.e9. 10.1016/j.ccell.2021.05.005 PMC8782207 34115987

[54] Izumi D, Ishimoto T, Sakamoto Y, Miyamoto Y, Baba H (2015). Molecular insights into colorectal cancer stem cell regulation by environmental factors. J Cancer Metastasis Treat.

[55] Zhou Y, Xia L, Wang H, Oyang L, Su M, Liu Q (2018). Cancer stem cells in progression of colorectal cancer. Oncotarget.

[56] Munoz J, Stange DE, Schepers AG, van de Wetering M, Koo B-K, Itzkovitz S (2012). The Lgr5 intestinal stem cell signature: Robust expression of proposed quiescent ‘+4’ cell markers. EMBO J.

[57] Barker N, Ridgway RA, van Es JH, van de Wetering M, Begthel H, van den Born M (2009). Crypt stem cells as the cells-of-origin of intestinal cancer. Nature.

[58] Kariya Y, Oyama M, Suzuki T, Kariya Y (2021). αvβ3 Integrin induces partial EMT independent of TGF-β signaling. Commun Biol.

[59] Xiong J-P, Stehle T, Zhang R, Joachimiak A, Frech M, Goodman SL (2002). Crystal structure of the extracellular segment of integrin alpha Vbeta3 in complex with an Arg-Gly-Asp ligand. Science.

[60] Stemmler MP, Eccles RL, Brabletz S, Brabletz T (2019). Non-redundant functions of EMT transcription factors. Nat Cell Biol.

[61] Yaeger R, Chatila WK, Lipsyc MD, Hechtman JF, Cercek A, Sanchez-Vega F (2018). Clinical sequencing defines the genomic landscape of metastatic colorectal cancer. Cancer Cell.

[62] Joyce JA, Pollard JW (2009). Microenvironmental regulation of metastasis. Nat Rev Cancer.

[63] Tauriello DVF, Calon A, Lonardo E, Batlle E (2017). Determinants of metastatic competency in colorectal cancer. Mol Oncol.

[64] Lu P, Weaver VM, Werb Z (2012). The extracellular matrix: a dynamic niche in cancer progression. J Cell Biol.

[65] Pape J, Magdeldin T, Stamati K, Nyga A, Loizidou M, Emberton M, et al. Cancer-associated fibroblasts mediate cancer progression and remodel the tumouroid stroma. Br J Cancer. 2020;123:1178–90. 10.1038/s41416-020-0973-9 PMC7524802 32641866

[66] Tsai JH, Donaher JL, Murphy DA, Chau S, Yang J (2012). Spatiotemporal regulation of epithelial-mesenchymal transition is essential for squamous cell carcinoma metastasis. Cancer Cell.

[67] Ocaña OH, Córcoles R, Fabra A, Moreno-Bueno G, Acloque H, Vega S (2012). Metastatic colonization requires the repression of the epithelial-mesenchymal transition inducer Prrx1. Cancer Cell.

[68] Schnappauf O, Beyes S, Dertmann A, Freihen V, Frey P, Jägle S (2016). Enhancer decommissioning by Snail1-induced competitive displacement of TCF7L2 and down-regulation of transcriptional activators results in EPHB2 silencing. Biochim Biophys Acta.

[69] Zheng X, Carstens JL, Kim J, Scheible M, Kaye J, Sugimoto H (2015). Epithelial-to-mesenchymal transition is dispensable for metastasis but induces chemoresistance in pancreatic cancer. Nature.

[70] el Marjou F, Janssen K-P, Chang BH-J, Li M, Hindie V, Chan L (2004). Tissue-specific and inducible Cre-mediated recombination in the gut epithelium. Genesis.

[71] Shibata H, Toyama K, Shioya H, Ito M, Hirota M, Hasegawa S (1997). Rapid colorectal adenoma formation initiated by conditional targeting of the Apc gene. Science.

[72] Tuveson DA, Shaw AT, Willis NA, Silver DP, Jackson EL, Chang S (2004). Endogenous oncogenic K-rasG12D stimulates proliferation and widespread neoplastic and developmental defects. Cancer Cell.

[73] Olive KP, Tuveson DA, Ruhe ZC, Yin B, Willis NA, Bronson RT (2004). Mutant p53 gain of function in two mouse models of Li-Fraumeni syndrome. Cell.

[74] Wenzel J, Rose K, Haghighi EB, Lamprecht C, Rauen G, Freihen V (2020). Loss of the nuclear Wnt pathway effector TCF7L2 promotes migration and invasion of human colorectal cancer cells. Oncogene.

[75] Nyström A, Thriene K, Mittapalli V, Kern JS, Kiritsi D, Dengjel J (2015). Losartan ameliorates dystrophic epidermolysis bullosa and uncovers new disease mechanisms. EMBO Mol Med.

[76] Eide PW, Bruun J, Lothe RA, Sveen A (2017). CMScaller: an R package for consensus molecular subtyping of colorectal cancer pre-clinical models. Sci Rep.

[77] Feng G, Du P, Krett NL, Tessel M, Rosen S, Kibbe WA, et al. A collection of bioconductor methods to visualize gene-list annotations. BMC Res Notes. 2010;3:10. https://pubmed.ncbi.nlm.nih.gov/20180973/. 10.1186/1756-0500-3-10 PMC2829581 20180973

[78] Cerami E, Gao J, Dogrusoz U, Gross BE, Sumer SO, Aksoy BA (2012). The cBio cancer genomics portal: an open platform for exploring multidimensional cancer genomics data. Cancer Discov.

[79] Gao J, Aksoy BA, Dogrusoz U, Dresdner G, Gross B, Sumer SO (2013). Integrative analysis of complex cancer genomics and clinical profiles using the cBioPortal. Sci Signal.

[80] Balwierz PJ, Pachkov M, Arnold P, Gruber AJ, Zavolan M, van Nimwegen E (2014). ISMARA: automated modeling of genomic signals as a democracy of regulatory motifs. Genome Res.

[81] RStudio Team. RStudio: Integrated Development Environment for R [Internet]. Boston, MA; 2015. Available from: http://www.rstudio.com/

[82] Fox J, Weisberg S. An R companion to applied regression. Third edition. Thousand Oaks, California: SAGE; 2019.

[83] Wickham H ggplot2: Elegant graphics for data analysis. Cham: Springer; 2016.

