# Supplementary material for: Canonical TGFβ signaling induces collective invasion in colorectal carcinogenesis through a Snail1- and Zeb1-independent partial EMT
Source: Oncogene. 2022 Jan 24;41(10):1492–506. doi: 10.1038/s41388-022-02190-4 (PMC8897192; doi:10.1038/s41388-022-02190-4)
Supplement: Supplementary file 1 — Supplementary information Flum et al [file 41388_2022_2190_MOESM1_ESM.pdf]

Supplementary Information for

Canonical TGFβ signaling induces collective invasion in colorectal carcinogenesis through a Snail1- and Zeb1-independent partial EMT

Marion Flum, Severin Dicks, Yu-Hsiang Teng, Monika Schrempp, Alexander Nyström, Melanie Boerries, and Andreas Hecht

This PDF file includes:

- Supplementary Figures 1 to 16
- Legend for Supplementary Movie 1
- Supplementary Methods
- Supplementary Information References

Supplementary Figures

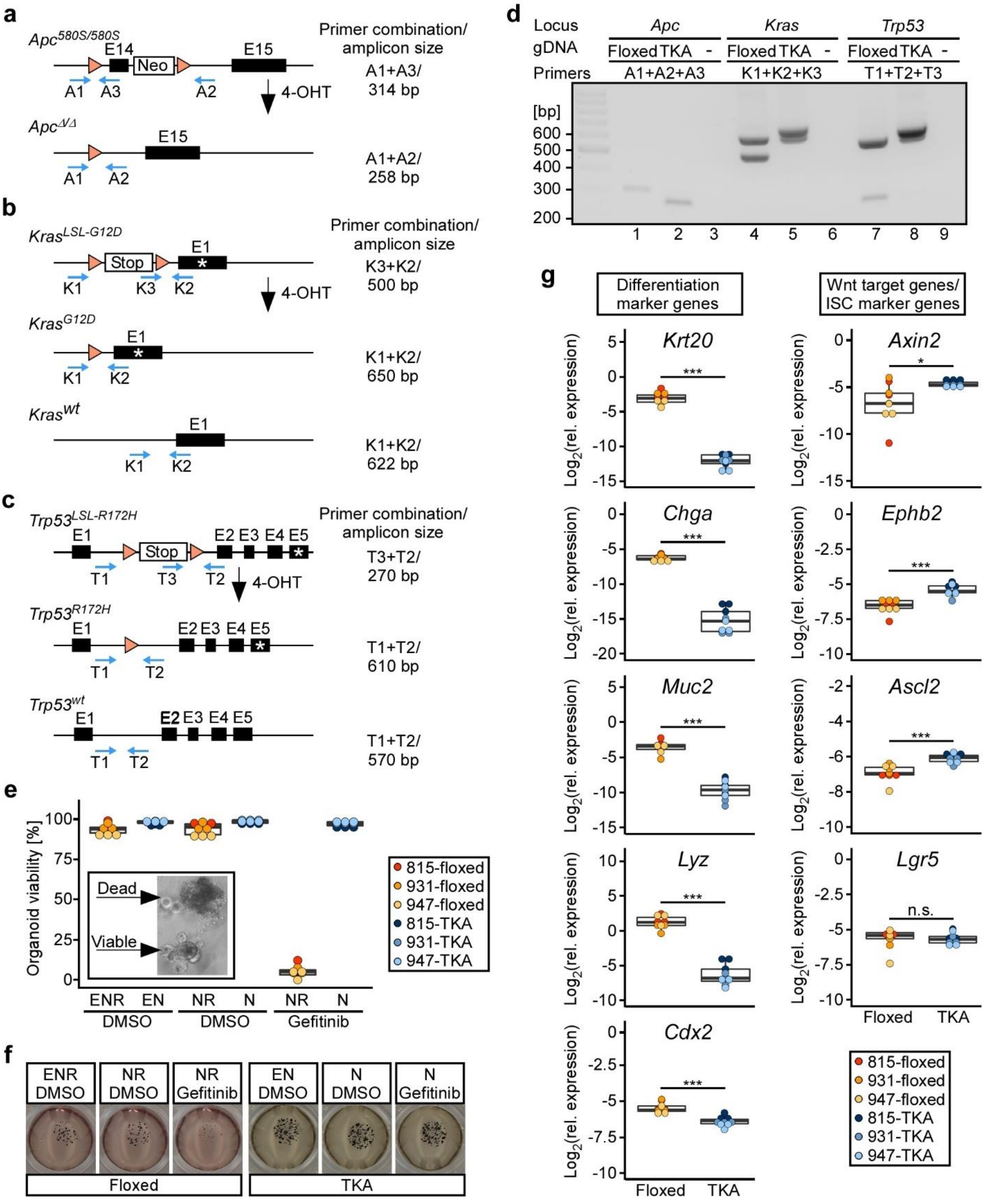

Supplementary figure 1: *In vitro* characterization of oncogenically transformed organoids. a-c, Schematic views of the *Apc*, *Kras*, and *Trp53* loci in small intestinal and colonic organoids derived from genetically engineered mice additionally carrying a *Villin-CreERT2* transgene. Black bars: exons (E) with locus-specific counting; orange triangles: LoxP elements; Neo: Neomycin resistance gene; Stop: stop cassette preventing expression of floxed alleles; blue arrows: locus-specific PCR primers; asterisks: position of point mutations. Primer

combinations used for genotyping and the expected amplicon lengths in base pairs (bp) for wildtype (wt), floxed, and recombined loci are listed to the right of the corresponding gene models. **a**, Genetically engineered mice carry two floxed alleles of the *Apc* gene. Treatment with 4-hydroxy-tamoxifen (4-OHT) leads to deletion of exon 14 and the adjacent Neo cassette ($Apc^{\Delta/\Delta}$). **b** and **c**, Genetically engineered mice are heterozygous at the *Kras* and *Trp53* loci with one allele being wildtype (wt) and the other being modified by insertion of floxed stop cassettes (LSL). Treatment with 4-OHT removes the LSL cassettes and results in expression of $Kras^{G12D}$ and $p53^{R172H}$. **d**, Gel electrophoresis separating DNA fragments generated by multiplex-PCRs with locus-specific primer combinations as indicated and genomic DNA (gDNA) isolated from floxed organoids and TKA organoids with recombined $Apc^{\Delta/\Delta}$, $Kras^{G12D}$ and $Trp53^{R172H}$ loci. Negative control samples received H_20 (-) as replacement for gDNA. **e**, Dependence of floxed and TKA organoids on EGFR signaling was determined by adding gefitinib to the organoid culture media supplemented with the indicated combinations of EGF (E), Noggin (N), and R-spondin-1 (R). Treatment with DMSO served as solvent control. After 72 h, organoid viability was judged by microscopy. Examples for viable and dead organoids are shown in the box. **f**, Representative images of MTT staining of floxed and TKA organoids (line 815) treated as described in (**e**). **g**, Gene expression analyses of marker genes for differentiated intestinal cells, intestinal stem cells (ISC), and Wnt target genes by qRT-PCR. Expression was normalized to *Gapdh*. ***$p<0.001$, *$p<0.05$; Mann-Whitney *U* test. Exact *p*-values are provided in Supplementary table 7. For (**e-g**) three independent biological replicates were performed for each of three different floxed and TKA organoid lines (815: n=3, 931: n=3, 947: n=3) seeded in 7 mg/ml Matrigel. Dots represent results of individual experiments while dot color identifies the organoid lines.

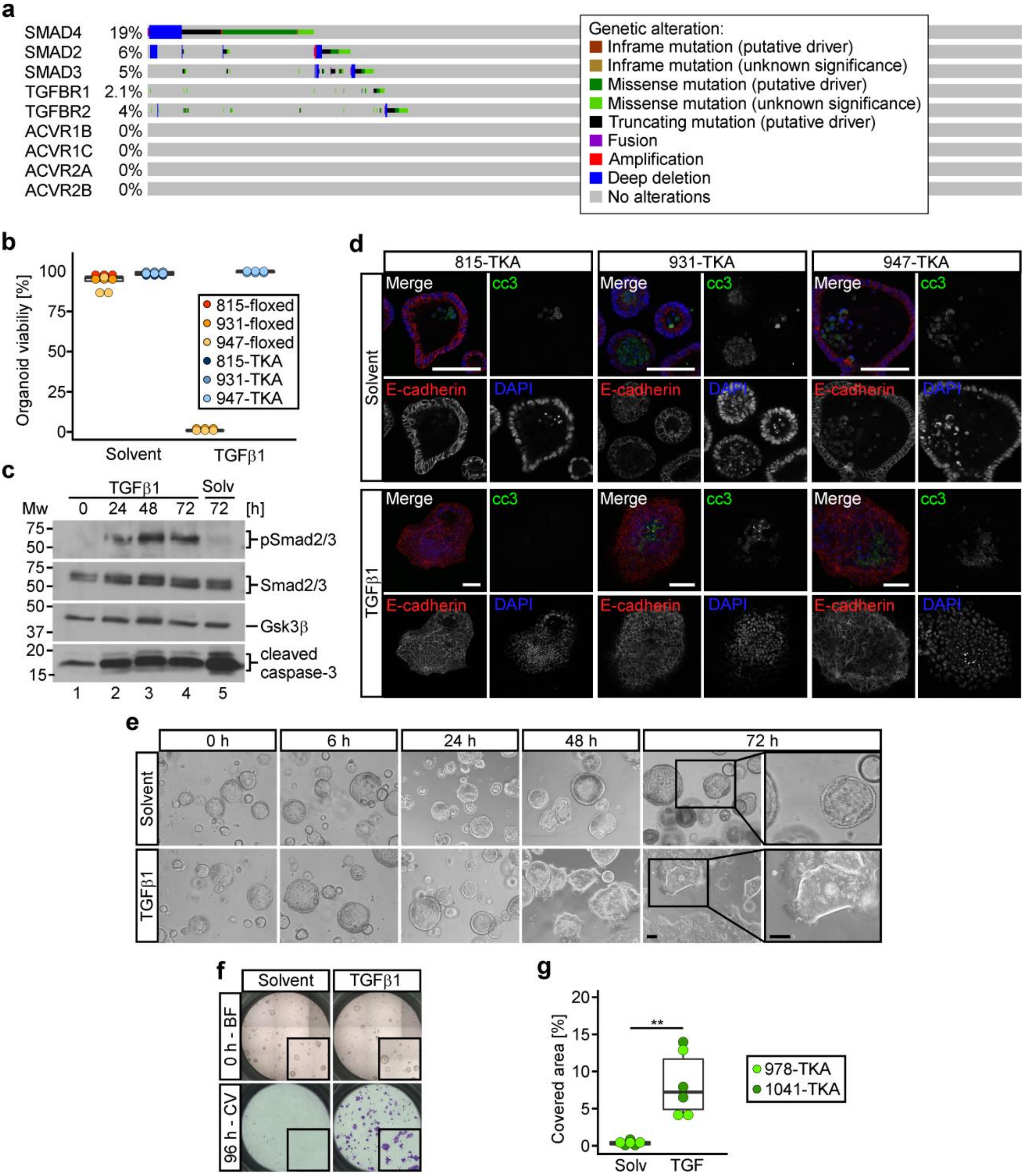

Supplementary figure 2: Apoptosis resistance of oncogenically transformed small intestinal organoids and TGFβ1-induced collective invasion of colonic TKA organoids. **a**, Nature and frequency of genetic alterations in components of the TGFβ signaling pathway in 1 134 CRC samples analyzed by cBioPortal. The majority (63.9%) of the examined CRC samples have no genetic alterations in the TGFβ signaling pathway. **b**, Floxed and TKA small intestinal organoids were seeded in 7 mg/ml Matrigel and treated with solvent or TGFβ1 for 72 h and organoid viability was judged by microscopy. Dots represent results of individual experiments while dot color identifies the organoid lines. Independent experiments were performed with three different floxed/TKA organoid lines, 815: n=3, 931: n=3, 947: n=3. **c**, Western blot analysis of cleaved caspase-3 in small intestinal TKA organoids (line 815) seeded

in 3 mg/ml Matrigel and treated with solvent or TGFβ1 for the indicated periods of time. Phosphorylation of Smad2/3 was analyzed as indication for active TGFβ signaling. Gsk3β detection served as loading control. Molecular weights of size standards are given in kDa. **d**, Whole mount immunofluorescence staining of small intestinal TKA organoids seeded in 3 mg/ml Matrigel and treated with solvent or TGFβ1 for 72 h. Organoids were stained for cleaved caspase-3 (cc3) and E-cadherin. Nuclei were stained by DAPI. For (**c**) and (**d**), three independent biological replicates were performed with three different organoid lines (815: n=1; 931: n=1; 947: n=1). Scale bars: 100 µm. **e**, Morphology of colonic TKA organoids (line 978) cultured in 3 mg/ml Matrigel and treated with solvent or TGFβ1 for the indicated periods of time. Boxed areas are shown at higher magnification on the right. Scale bars: 100 µm. **f**, Boyden chamber invasion assays with colonic TKA organoids (line 1041) seeded in 3 mg/ml Matrigel. Top: bright field (BF) images taken at 0 h of solvent and TGFβ1 treatment. Inserts show magnified views of the upper chambers. Bottom: crystal violet (CV) staining of invaded cells after 96 h of treatment. Inserts show magnified views of the bottom faces of the Boyden chambers. **g**, Quantification of invasion experiments as shown in (**f**) performed with colonic TKA organoid lines 978 (n=3) and 1041 (n=3). Each dot represents the result of a single invasion assay while dot color identifies the organoid lines. Solv: solvent; TGF: TGFβ1. **p=0.0022; Mann-Whitney U test.

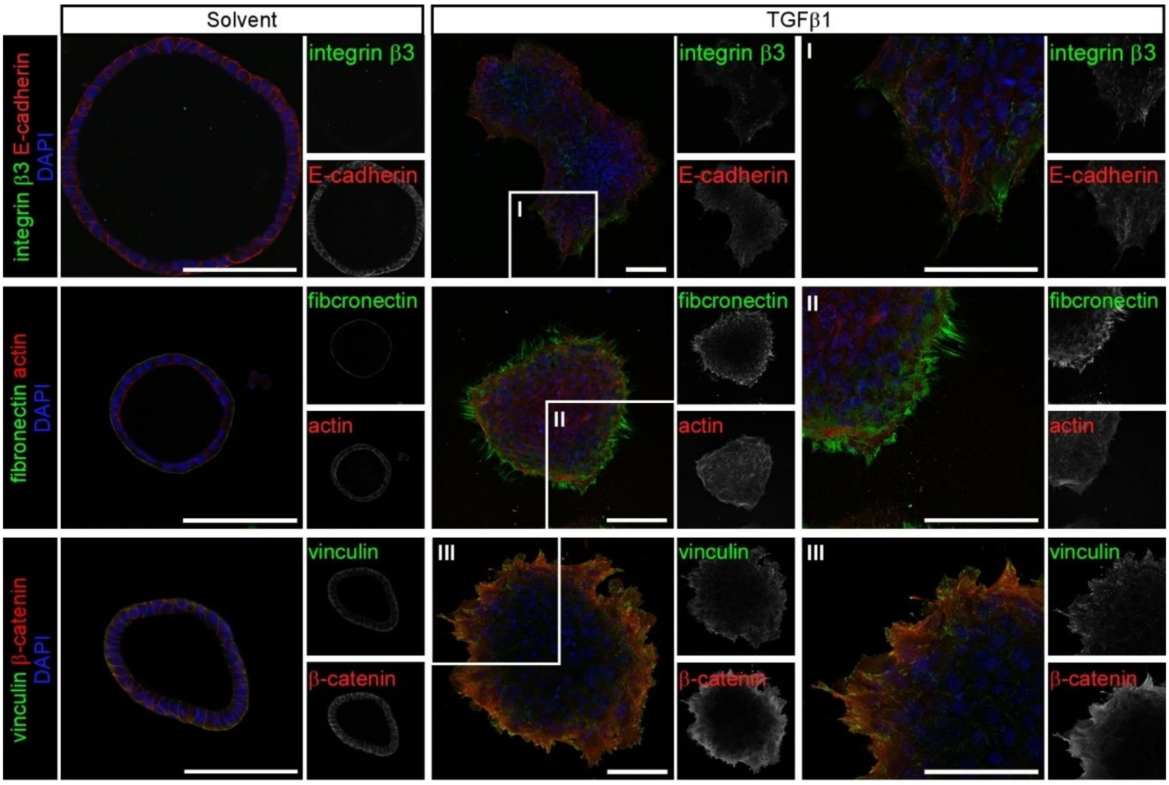

Supplementary figure 3: Formation of fibronectin spikes and focal adhesions at the invasive front of TGFβ1-stimulated TKA organoids. Whole mount immunofluorescence staining and confocal microscopy of TKA organoids (line 931) cultured in 3 mg/ml Matrigel and treated with solvent or TGFβ1 for 72 h. Organoids were stained with the indicated antibody combinations against integrin β3, E-cadherin, fibronectin, vinculin, and β-catenin. Actin was visualized by phalloidin staining. Nuclei were labeled with DAPI. Boxed areas I, II, and III are shown at higher magnification on the right. Pictures are representative for results obtained with three different TKA organoid lines (815: n=1, 931: n=1, 947: n=1). Scale bars: 100 µm.

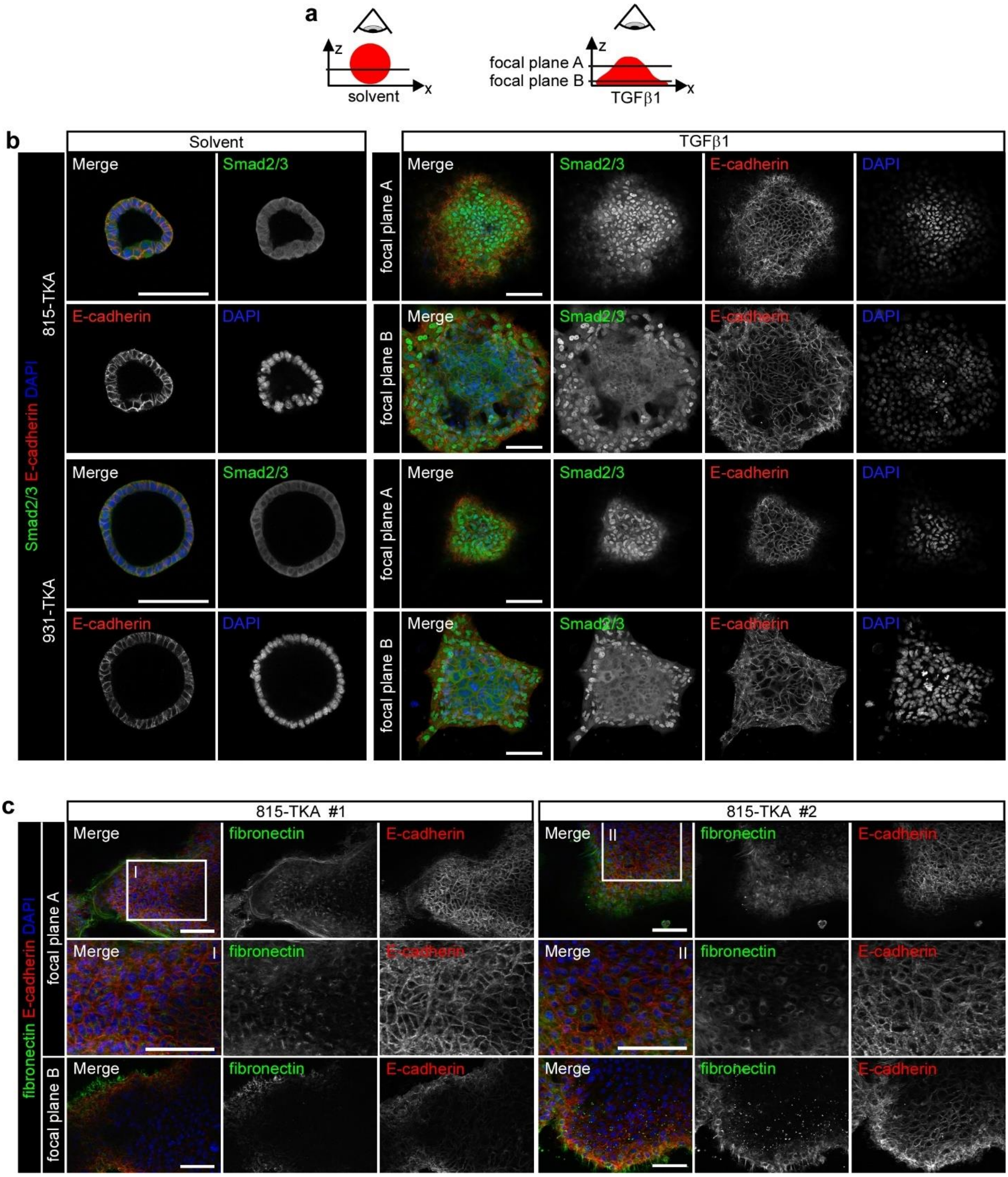

Supplementary figure 4: Activation of TGFβ signaling and mesenchymal marker gene expression in the dome structure of TGFβ1-stimulated TKA organoids. a, Direction of view and position of focal planes for images shown in (**b**) and (**c**). **b**, Whole mount immunofluorescence staining and confocal microscopy of TKA organoid lines 815 and 931 seeded in 3 mg/ml Matrigel and treated with solvent or TGFβ1 for 72 h. Organoids were stained for Smad2/3 and E-cadherin. Nuclei were visualized with DAPI. TGFβ1-stimulated organoids were imaged at focal planes within the dome structure (plane A) and at their base close to the bottom of the cell culture plates (plane B). **c**, Whole mount immunofluorescence staining and fluorescence microscopy of TKA organoids (line 815) treated as in (**b**). Organoids were stained for fibronectin and E-cadherin. Nuclei were labeled with DAPI. Boxed areas I and II in images

of focal plane A are displayed at higher magnification in the panels underneath. Shown are two representative examples (line 815-TKA organoids #1 and #2) of three independent biological replicates, n=3. Images were acquired using an Axio Observer.Z1 fluorescence microscope with an ApoTome2 equipment (Zeiss). Scale bars: 100 µm.

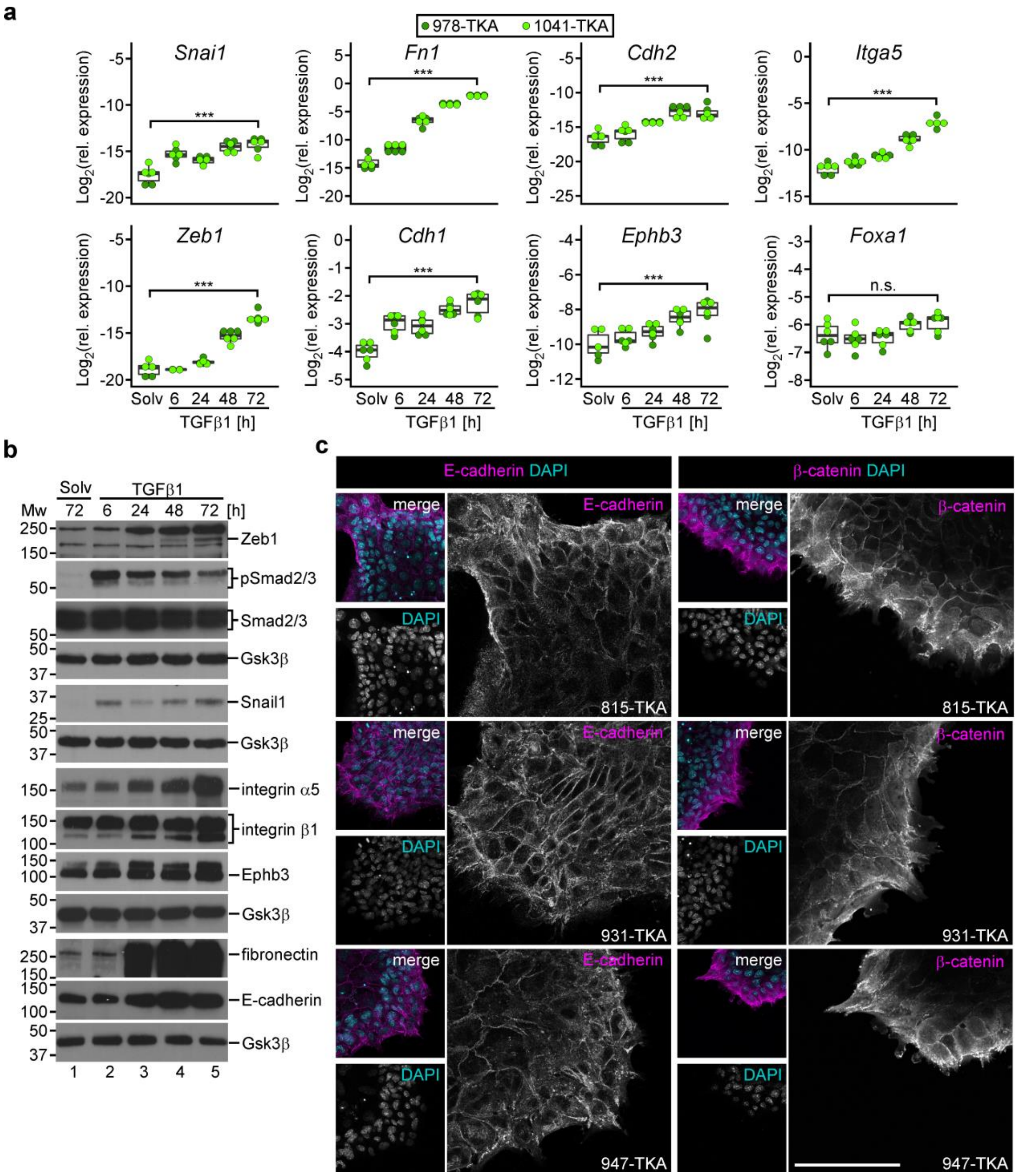

Supplementary figure 5: TGFβ1 induces a partial EMT in oncogenically transformed small intestinal and colonic organoids. a, Gene expression analysis of colonic TKA organoids seeded in 3 mg/ml Matrigel and treated with solvent (solv) or TGFβ1 for the indicated periods of time. Gene-specific transcripts of EMT-TFs and EMT-associated genes were quantified by qRT-PCR and normalized to transcript levels of *Eef1a1*. Each dot represents the result of a single measurement while dot color identifies the organoid lines. Three independent biological replicates were performed for two organoid lines (978: n=3; 1041: n=3). ****p*<0.001, n.s.: not significant; statistical significance was analyzed using a linear model combined with Bonferroni correction for multiple comparisons. Exact *p*-values are provided in Supplementary table 7. **b**, Western blot analyses of phosphorylated Smad2/3 (pSmad2/3), total Smad2/3, EMT-TFs, and EMT-associated genes in colonic TKA organoids (line 978) treated as in (**a**).

Molecular weights of size standards are given in kDa. Gsk3β detection served as loading control (n=3). **c**, Additional examples for whole mount immunofluorescence stainings and confocal microscopy of TKA organoid lines 815, 931, and 947 treated with TGFβ1 for 72 h. Organoids were stained for E-cadherin or β-catenin. Nuclei were labelled using DAPI. Scale bar: 100 µm.

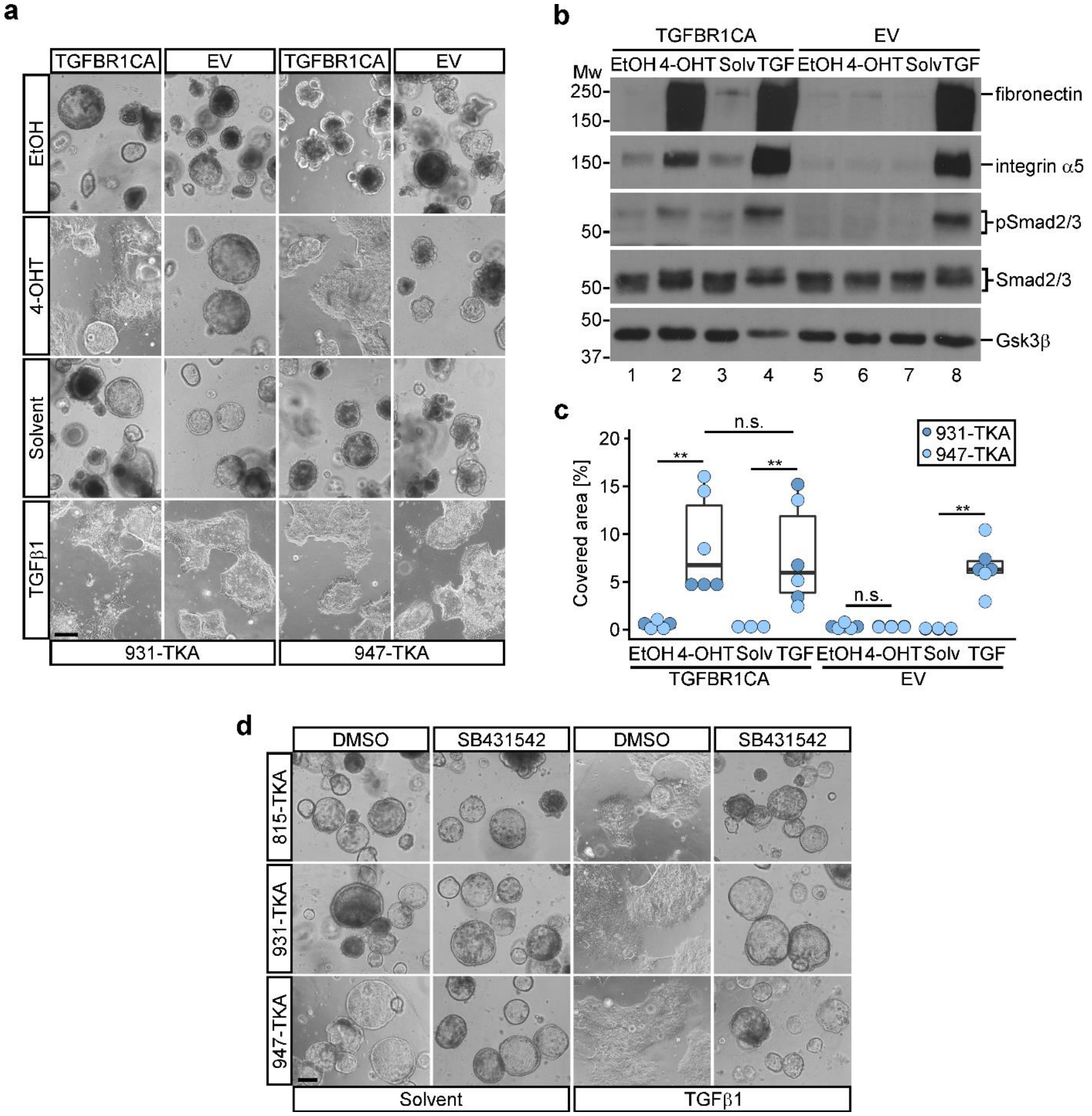

Supplementary figure 6: The TGFβ1 response in TKA organoids is mediated by genuine TGFβ receptor activity. a-c, TKA organoids, transduced with an empty vector (EV) or a vector for inducible expression of a constitutively active form of TGFBR1 (TGFBR1CA), were seeded in 3 mg/ml Matrigel and treated with ethanol (EtOH) or 4-OHT for 96 h to trigger TGFBR1CA production. Alternatively, organoids received solvent (solv) or TGFβ1 (TGF) for 72 h. **a**, Whole mount phase contrast microscopy of transduced TKA organoids. Two independent biological replicates were performed with two different organoid lines (931: n=2, 947: n=2) Scale bar: 200 µm. **b**, Western blot analyses of phosphorylated Smad2/3 (pSmad2/3), total Smad2/3, fibronectin, and integrin α5 in transduced TKA organoid line 947 treated as in (**a**). Gsk3β detection served as loading control. Molecular weights of size standards are given in kDa. Results shown are representative for three independent biological replicates performed with organoid lines 931 (n=3) and 947 (n=3). **c**, Quantification of Boyden chamber invasion assays performed with transduced organoids as described in (**a**). Three independent biological replicates were performed with each of two different TKA organoid lines (931: n=3, 947: n=3). Dots represent results of individual experiments. Dot color identifies the organoid lines.

$p<0.01$, n.s.: not significant; Mann-Whitney *U* test. Exact *p*-values are provided in Supplementary table 7. **d, Whole mount phase contrast microscopy of TKA organoids seeded in 3 mg/ml Matrigel and treated with solvent or TGFβ1 in presence of DMSO or SB431542 for 72 h. Images shown are representative for three independent biological replicates performed with three different organoid lines (815: n=3, 931: n=3, 947: n=3). Scale bar: 200 µm.

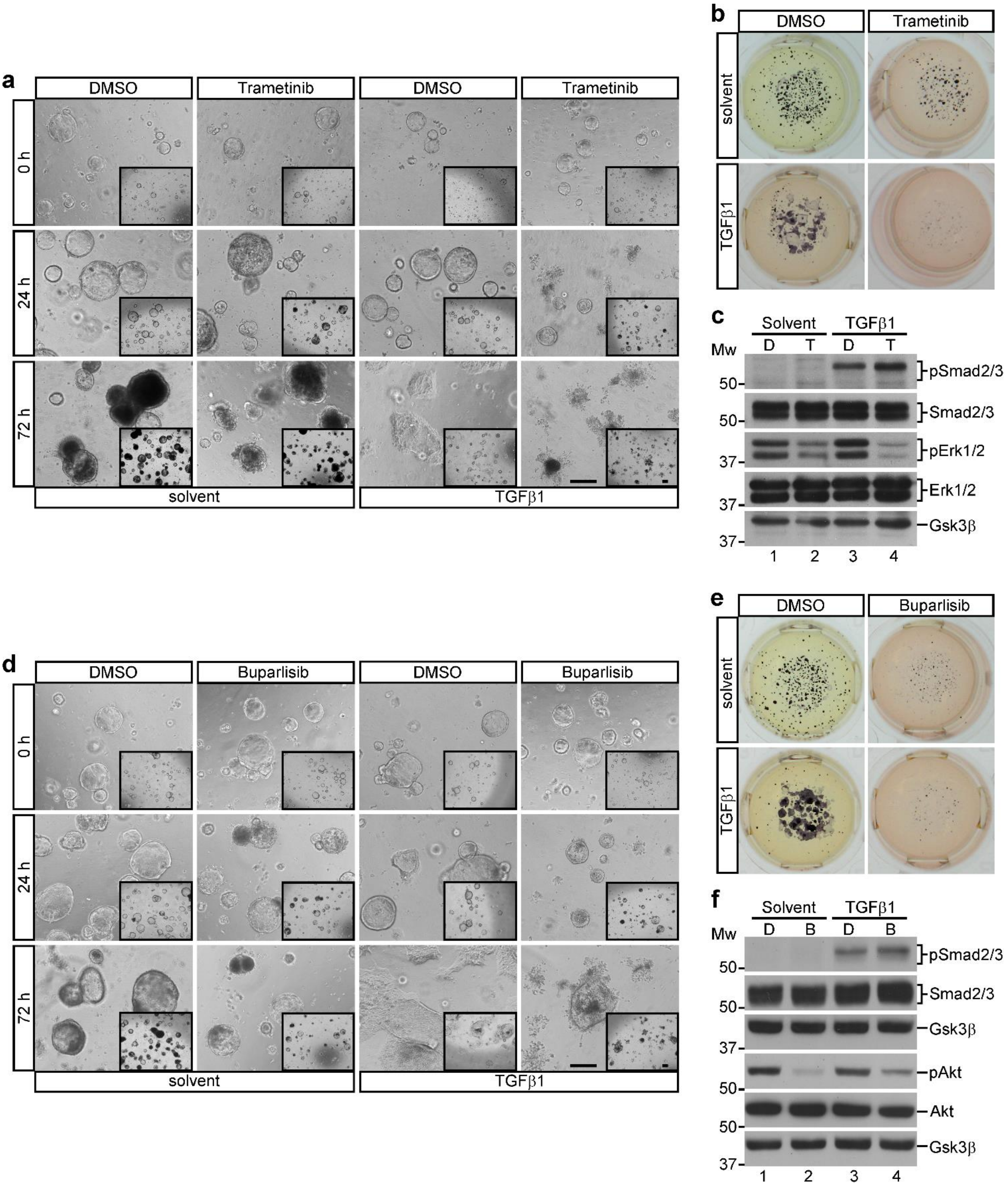

Supplementary figure 7: Inhibition of MEK1/2 and PI3 kinases alleviates resistance against TGFβ1-induced cell death and impairs viability of TKA organoids, respectively. **a**, Whole mount phase contrast microscopy of small intestinal TKA organoids (line 947) treated with the indicated combinations of solvent, TGFβ1, DMSO, and the MEK inhibitor trametinib for 72 h. Images were acquired at 0 h, 24 h, and 72 h of stimulation. Scale bars: 200 µm. **b**, Representative images of MTT stainings of TKA organoids (line 931) treated for 72 h as described in (**a**). **c**, Western blot analyses of phosphorylated Smad2/3 (pSmad2/3), total Smad2/3, phosphorylated Erk1/2 (pErk1/2), and total Erk1/2 in TKA organoids (line 947) treated with the indicated combinations of solvent, TGFβ1, DMSO (D), and Trametinib (T) for 22 h. Molecular weights of size standards are given in kDa. Gsk3β detection served as loading control. **d**, Whole mount phase contrast microscopy of small intestinal TKA organoids (line

815) treated with the indicated combinations of solvent, TGFβ1, DMSO, and the PI3K inhibitor buparlisib for 72 h. Images were acquired at 0 h, 24 h, and 72 h of stimulation. Scale bars: 200 µm. **e**, Representative images of MTT stainings of TKA organoids (line 931) treated for 72 h as described in (**d**). **f**, Western blot analyses of phosphorylated Smad2/3 (pSmad2/3), total Smad2/3, phosphorylated Akt (pAkt), and total Akt in TKA organoids (line 931) treated with the indicated combinations of solvent, TGFβ1, DMSO (D), and Buparlisib (B) for 22 h. Molecular weights of size standards are given in kDa. Gsk3β detection served as loading control. **a**-**e**: TKA organoid lines were seeded in 3 mg/ml Matrigel. Morphological examinations (**a**, **d**) and MTT stainings (**b**, **e**) were performed once for each line (815: n=1, 931: n=1, 947: n=1). For Western blot analyses one or two independent experiments were carried out with each TKA organoid line [(**c**) 815: n=1, 931: n=1, 947: n=1; (**f)** 815: n=2, 931: n=2, 947: n=2].

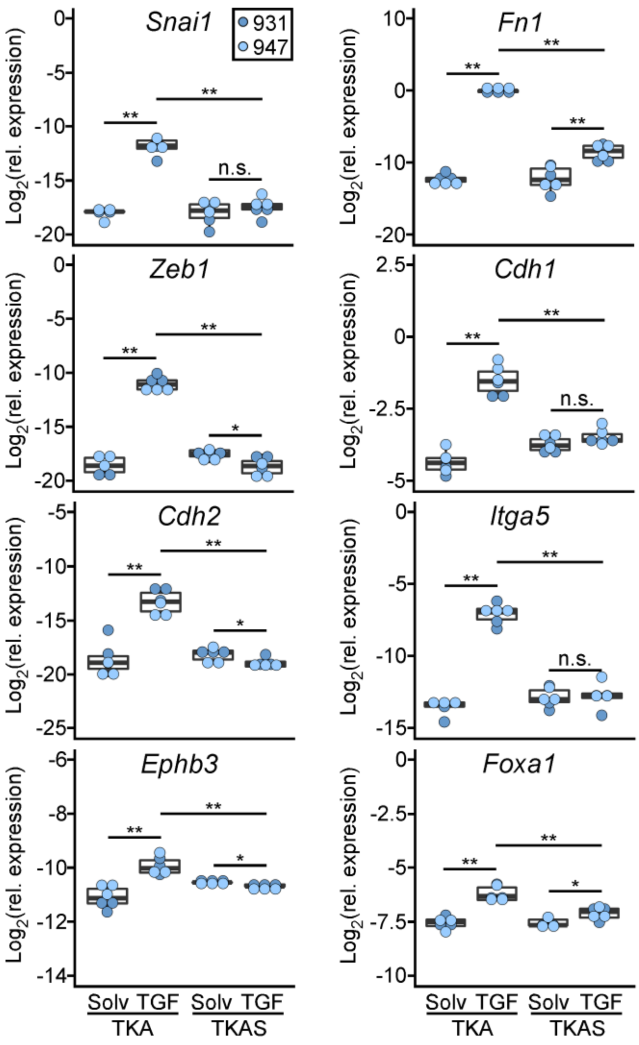

Supplementary figure 8: The TGFβ1 response in TKA organoids depends on Smad4. Gene expression analyses of *Snai1, Zeb1, Fn1*, *Cdh2*, *Itga5*, *Cdh1*, *Ephb3,* and *Foxa1* in TKA and TKAS organoids seeded in 3 mg/ml Matrigel and stimulated with solvent (solv) or TGFβ1 (TGF) for 72 h. Gene-specific transcripts were quantified by qRT-PCR and normalized to transcript levels of *Eef1a1*. Three independent biological replicates were performed with TKA and TKAS organoids derived from lines 931 (n=3) and 947 (n=3). Dots represent results of individual experiments while dot color identifies the organoid lines. **$p<0.01$, *$p<0.05$, n.s.: not significant; Mann-Whitney *U* test. Exact *p*-values are provided in Supplementary table 7.

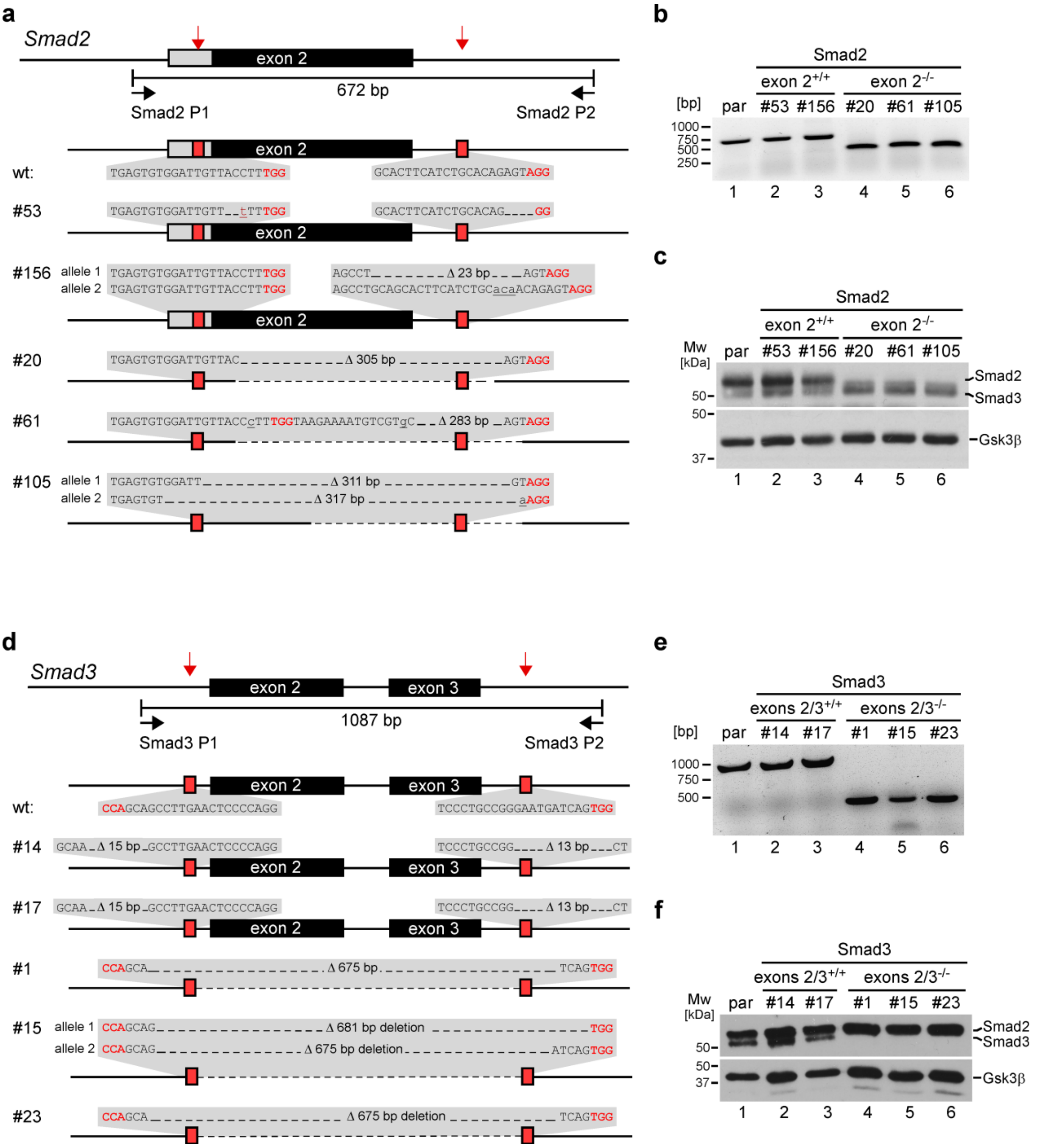

Supplementary figure 9: Inactivation of the *Smad2* and *Smad3* genes in TKA organoids. Cells from the TKA organoid line 931 were transduced with expression vectors for Cas9 and sgRNAs targeting the *Smad2* and *Smad3* loci. Transduced cells were selected and used to isolate single-cell derived organoid lines. **a**, **d**, Upper parts: schemes of the genomic regions around *Smad2* exon 2 and *Smad3* exons 2/3. *Smad2* exon 2 is shown as gray/black box (gray: untranslated region; black: translated region). *Smad3* exons 2/3 are depicted as black boxes. The locations of the sgRNA target sites (red arrows) and of PCR primers Smad2 P1/P2 and Smad3 P1/P2 (black arrows) are shown. The sizes of the amplicons are given in base pairs (bp). Lower parts: Sequence alterations around *Smad2* exon 2 and *Smad3* exons 2/3 as determined by amplification of genomic DNA regions with locus-specific primer pairs and Sanger sequencing of the PCR products. The wildtype (wt) sequence of the sgRNA target sites (red boxes) and the sequence changes in the clonal organoid lines are highlighted in gray. Deletions are indicated with dashed lines and the number of base pairs missing is shown.

Lower case and underlining mark substitutions and insertions. Bold red letters: PAM sequences. **b**, **e**, PCR amplification of genomic sequences with locus-specific primer pairs to confirm the presence of large-scale deletions in *Smad2* and *Smad3* in the respective organoid lines. As control, PCR products derived from the parental (par) TKA organoid line 931 were analyzed in parallel. Presence or absence of *Smad2* exon 2 and *Smad3* exons 2/3 in the genomes of the organoid lines under investigation is denoted. The lengths of size standards are given in base pairs (bp). **c**, **f**, Western blotting was used to examine expression of Smad2 and Smad3 in the organoid lines indicated. Presence or absence of *Smad2* exon 2 and *Smad3* exons 2/3 in the genomes of the organoid lines under investigation is denoted. As control, protein lysates from the parental (par) TKA organoid line 931 were analyzed in parallel. Detection of Gsk3β served as loading control. Molecular weights of size standards are given in kDa.

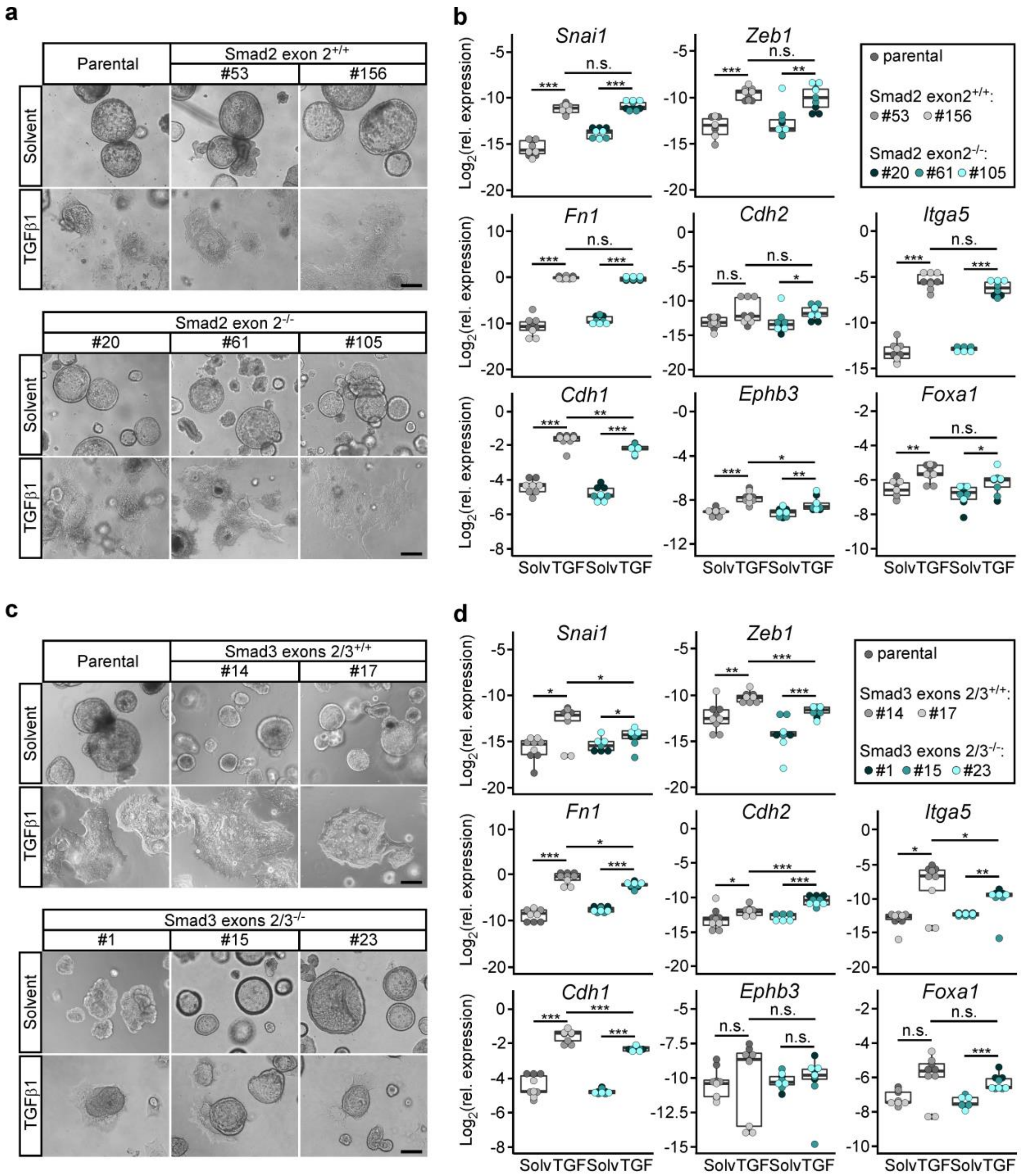

Supplementary figure 10: Differential requirements for Smad2 and Smad3 in TGFβ1-induced collective invasion and partial EMT of TKA organoids. a, **c**, Whole mount phase contrast microscopy of clonal derivatives of TKA organoid line 931 treated with solvent or TGFβ1 for 72 h (n=3). Presence or absence of *Smad2* exon 2 and *Smad3* exons 2/3 in the genomes of the organoid lines under investigation is denoted. Organoids were seeded in 3 mg/ml Matrigel. Scale bars: 200 µm. **b**, **d**, Gene expression analysis of EMT-TFs and EMT-associated genes in clonal derivatives of TKA organoid line 931 seeded and stimulated as described in (**a**, **b**). Presence or absence of *Smad2* exon 2 and *Smad3* exons 2/3 in the genomes of the organoid lines under investigation is denoted. Gene-specific transcripts were quantified by qRT-PCR and normalized to transcript levels of *Eef1a1* (n=3). Each dot represents the result of a single measurement while dot color identifies the organoid clone.

*** $p<0.001$, ** $p<0.01$, * $p<0.05$, n.s.: not significant; statistical significance was analyzed using the Mann-Whitney *U* test. Exact *p*-values are provided in Supplementary table 7.

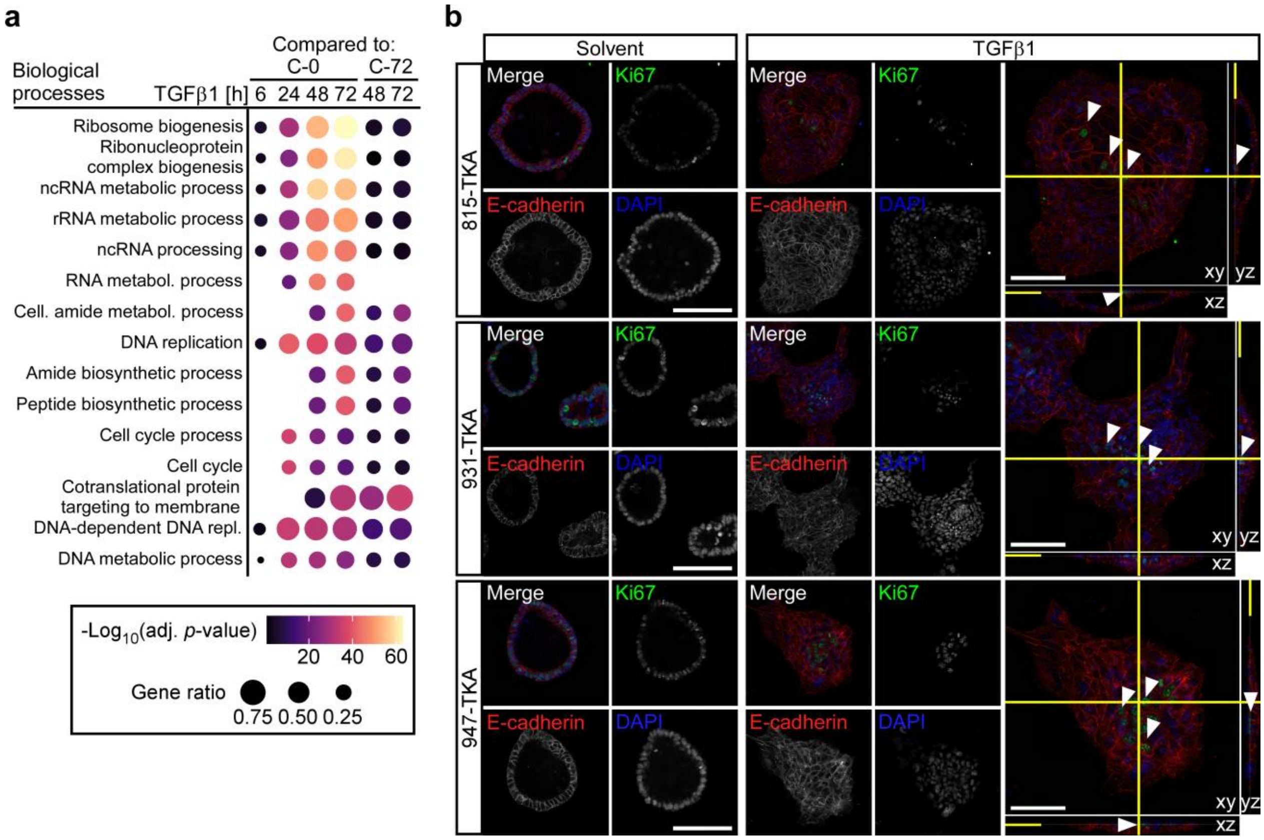

Supplementary figure 11: TGFβ1-induced gene expression changes in TKA organoids indicate repression of proliferation. a, Functional enrichment analysis of genes downregulated upon TGFβ1 treatment. Downregulated genes were determined by comparing transcriptomes of organoids treated with TGFβ1 for the indicated periods of time to those of organoids harvested immediately at the onset of the experiment (0 h of cultivation; C-0) or cultivated for 72 h in solvent (C-72) using an adjusted (adj.) *p*-value<0.01 and $\log_2$(FC)<-1 as thresholds. The top fifteen GO terms from the category "Biological processes" significantly enriched among downregulated genes are listed. Dot size reflects the ratio of downregulated genes compared to all genes in each GO term, while the color encodes the $-\log_{10}$(adj. *p*-value) of the enrichment. **b**, Whole mount immunofluorescence staining and confocal microscopy of TKA organoids seeded in 3 mg/ml Matrigel and treated with solvent or TGFβ1 for 72 h. Organoids were stained for Ki67 and E-cadherin. Nuclei were visualized with DAPI. Arrowheads mark a few remaining Ki67-positive cells in the center of TGFβ1-stimulated organoids. The right panel displays orthogonal views of cross-sections of TGFβ1-stimulated organoids. Yellow lines indicate the positions of the cross-sections along the x-, y-, and z-axes. Images show representative results of two independent biological replicates obtained with the different organoid lines indicated (n=2). Scale bars: 100 µm.

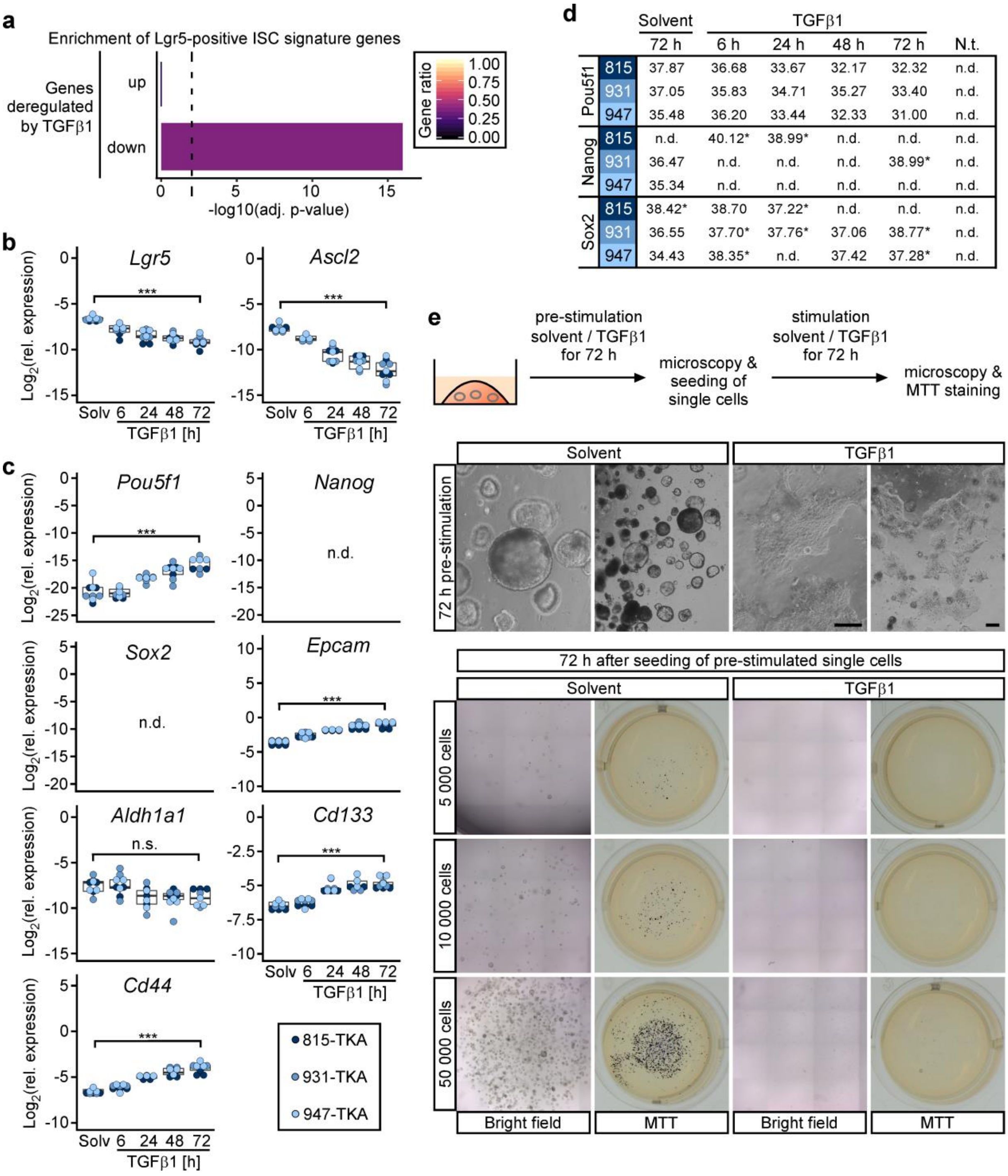

		Solvent 72 h	TGFβ1 6 h	TGFβ1 24 h	TGFβ1 48 h	TGFβ1 72 h	N.t.
Pou5f1	815	37.87	36.68	33.67	32.17	32.32	n.d.
	931	37.05	35.83	34.71	35.27	33.40	n.d.
	947	35.48	36.20	33.44	32.33	31.00	n.d.
Nanog	815	n.d.	40.12*	38.99*	n.d.	n.d.	n.d.
	931	36.47	n.d.	n.d.	n.d.	38.99*	n.d.
	947	35.34	n.d.	n.d.	n.d.	n.d.	n.d.
Sox2	815	38.42*	38.70	37.22*	n.d.	n.d.	n.d.
	931	36.55	37.70*	37.76*	37.06	38.77*	n.d.
	947	34.43	38.35*	n.d.	37.42	37.28*	n.d.

Supplementary figure 12: TGFβ1 treatment results in downregulation of intestinal stem cell signature gene expression and abrogates self-renewal capacity of TKA organoids. **a**, Functional enrichment analysis of signature genes from Lgr5-positive intestinal stem cells (ISC) among genes up- and downregulated in TKA organoids. Differentially expressed genes were determined by comparing transcriptomes of organoids treated with TGFβ1 for 72 h to those of organoids cultivated for 72 h in solvent using an adjusted (adj.) *p*-value<0.01 and an absolute value of $\log_2$(FC) > 1 as thresholds. The color of the bars encodes the ratio of deregulated genes compared to all genes of the ISC signature. **b** and **c**, Time-resolved gene expression analyses of small intestinal TKA organoids treated with solvent or TGFβ1 for the indicated time periods. RNA levels of the ISC signature genes *Lgr5* and *Ascl2* (**b**) and genes reported to be associated with stemness of cancer cells (**c**) were quantified by qRT-PCR.

Transcript levels of *Eef1a1* were used for normalization. Dots represent results of individual experiments while dot color identifies the organoid lines. Independent biological replicates were performed with three different organoid lines (815: n = 3; 931: n = 3; 947: n = 3). ****p*<0.001, **p*<0.05, n.s.: not significant; statistical significance was analyzed using a linear model combined with Bonferroni correction for multiple comparisons. Exact *p*-values are provided in Supplementary table 7. n.d.: no detection. **d**, Expression analysis of *Pou5f1*, *Nanog*, and *Sox2* by qRT-PCR in TKA organoids treated with solvent or TGFβ1 for the indicated periods of time. Presented are mean Ct-values calculated from technical duplicates of one representative experiment. N.t.: no template control, n.d.: no detection, *: value from only one of the two technical replicates is shown because the other had no detectable fluorescence signal after 40 cycles of amplification. **e**, Top: Schematic view of the experimental setup. Two days after seeding of mechanically disrupted small intestinal TKA organoids, treatment with solvent or TGFβ1 was started. After 72 h, phase contrast microscopy was conducted before pre-stimulated TKA organoids were dissociated and the indicated numbers of single cells were seeded in 50 µl droplets of 3 mg/ml Matrigel per well of a 24-well plate. Treatment with solvent or TGFβ1 was continued for another 72 h before organoids were imaged by bright field microscopy and stained by incubation with the MTT reagent. Middle: Whole mount phase contrast microscopy of TKA organoids (line 947) treated with solvent or TGFβ1 for 72 h. Scale bars: 200 µm. Bottom: Representative bright field images taken at 72 h after seeding of pre-stimulated single cells and MTT staining thereof (line 931). Three independent biological replicates were performed with three different TKA organoid lines (815: n=1, 931: n=1, 947: n=1) seeded in 3 mg/ml Matrigel.

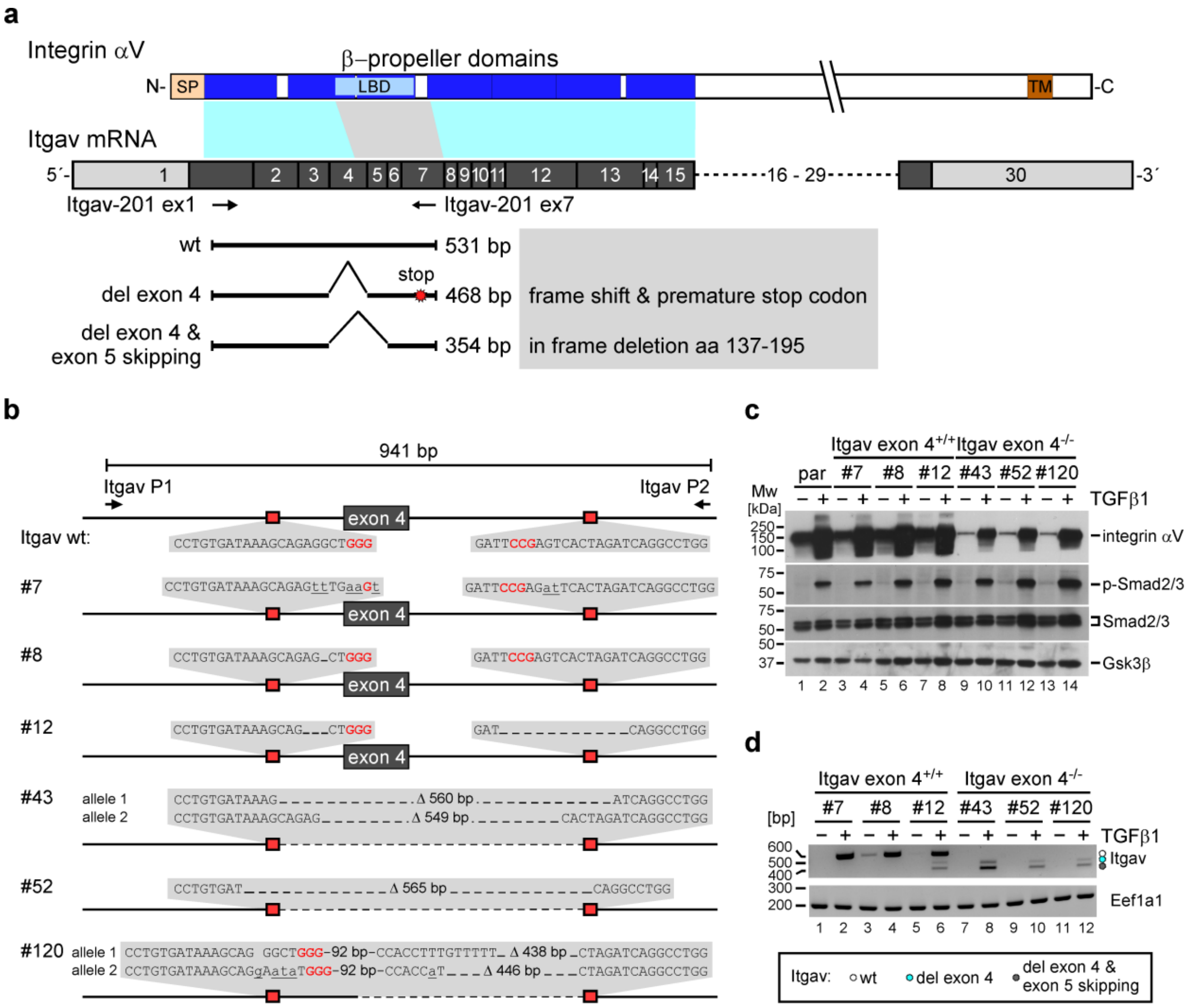

Supplementary figure 13: Generation and characterization of clonal TKA organoids expressing non-functional integrin αV. a, Schematic representation of integrin αV protein structure and the corresponding *Itgav* transcript (Itgav-201; ENSMUST00000028499.11). Blue boxes depict the seven domains which form the β-propeller and harbor the ligand binding domain (LBD; light blue) of integrin αV (1). They are aligned with the exons by which they are encoded. SP: signal peptide; TM: transmembrane domain. Also shown are the locations of primers in exons 1 and 7 which were used for amplification of *Itgav* cDNA fragments. The length and exon composition of PCR amplicons obtained from wildtype (wt) and exon 4-deleted organoids is given as well. As indicated in the gray box, deletion of exon 4 (del exon 4) only results in a reading frame shift and premature stop codon (red asterisk) in exon 7. Exon 4 deletion and concomitant skipping of exon 5 leads to an in frame deletion of amino acids (aa) 137-195 which comprise several critical residues of the LBD (1). **b**, Cells from the TKA organoid line 815 were transduced with expression vectors for Cas9 and *Itgav*-specific sgRNAs. Transduced cells were selected and used to isolate single-cell derived organoid lines. Sequence alterations around *Itgav* exon 4 were determined by amplification of genomic DNA regions with primers Itgav P1 and Itgav P2, and Sanger sequencing of the PCR products. The wt sequence of the *Itgav* sgRNA target sites (red boxes) and the *Itgav* sequence changes in the clonal organoid lines are highlighted in gray. Deletions are indicated with dashed lines. Lower case and underlining mark substitutions and insertions. Bold red letters: PAM sequences. **c**, Western blotting was used to examine expression of integrin αV, phosphorylated Smad2/3 (p-Smad2/3), and total Smad2/3 in organoid lines treated with

solvent or TGFβ1 for 72 h as indicated. Presence or absence of *Itgav* exon 4 in the genomes of the organoid lines under investigation is denoted. As control, protein lysates from the parental (par) TKA organoid line 815 were analyzed in parallel. Detection of Gsk3β served as loading control. Molecular weights of size standards are given in kDa. **d**, Analyses of *Itgav* transcript structure in clonal TKA organoid lines treated with solvent or TGFβ1 for 72 h as indicated. RNA was isolated from the organoids and reverse transcribed. The resulting cDNA was used to amplify *Itgav* sequences with the primers shown in (**a**). The exon composition of the differently sized PCR amplicons marked with colored asterisks was confirmed by Sanger sequencing. Successful amplification of *Eef1a1* sequences demonstrates presence of intact cDNA in all samples. Presence or absence of *Itgav* exon 4 in the genomes of the organoid lines under investigation is denoted. The lengths of size standards are given in base pairs (bp).

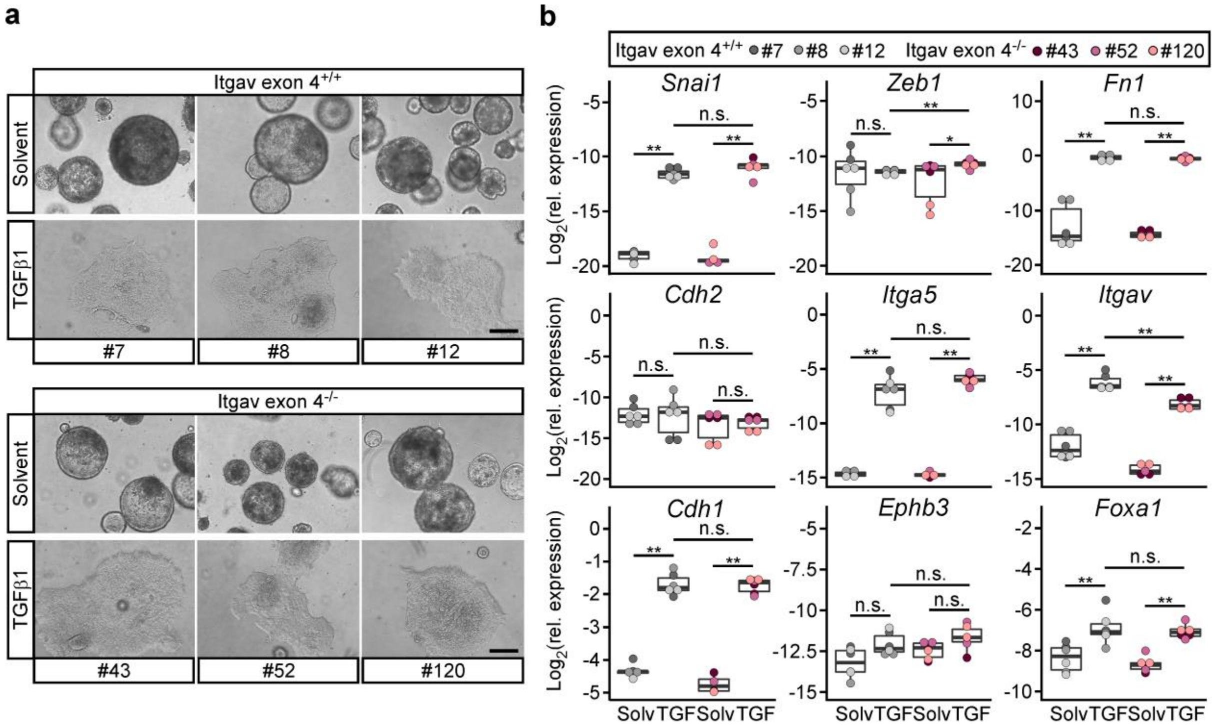

Supplementary figure 14: Integrin αV functionality is not required for TGFβ1-induced collective invasion and partial EMT of TKA organoids. a, Whole mount phase contrast microscopy of clonal derivatives of TKA organoid line 815 treated with solvent or TGFβ1 for 72 h (n=2). Presence or absence of *Itgav* exon 4 in the genomes of the organoid lines under investigation is denoted. Organoids were seeded in 3 mg/ml Matrigel. Scale bars: 200 µm. **b,** Gene expression analysis of EMT-TFs and EMT-associated genes in clonal derivatives of TKA organoid line 815 seeded and stimulated as described in (**a**). Presence or absence of *Itgav* exon 4 in the genomes of the organoid lines under investigation is denoted. Gene-specific transcripts were quantified by qRT-PCR and normalized to transcript levels of *Eef1a1* (n=2). Each dot represents the result of a single measurement while dot color identifies the organoid clone. ***p*<0.01, **p*<0.05, n.s.: not significant; statistical significance was analyzed using the Mann-Whitney *U* test. Exact *p*-values are provided in Supplementary table 7.

a

		Solvent	TGFβ1				
		72 h	6 h	24 h	48 h	72 h	N.t.
Snai1	815	33.86	33.71	31.65	28.56	27.66	n.d.
	931	33.27	32.95	31.15	28.43	27.59	38.71*
	947	33.28	33.47	32.64	29.15	28.41	n.d.
Snai2	815	34.09	35.37	35.57	32.88	32.00	38.61
	931	34.74	35.17	35.05	32.87	31.76	38.42
	947	34.05	35.18	34.74	33.40	31.76	38.93*
Zeb1	815	34.52	34.48	33.31	29.94	28.62	n.d.
	931	33.31	34.67	32.33	28.97	28.15	n.d.
	947	33.85	35.40	33.33	30.17	29.38	n.d.
Zeb2	815	39.71*	39.59*	39.9*	39.66*	34.33	37.48*
	931	38.41*	39.33*	39.88*	39.70*	37.49	39.03
	947	39.87*	38.74*	n.d.	39.94*	38.72	39.475
Twist1	815	33.85	35.40	34.82	35.73	34.57	n.d.
	931	33.61	33.57	33.87	35.73	34.60	n.d.
	947	33.77	33.88	34.28	35.42	34.18	n.d.
Twist2	815	36.88	37.25	37.89	36.77	38.04	n.d.
	931	36.53	36.21	36.63*	35.93	37.51*	n.d.
	947	37.36*	36.33*	36.05*	37.97	38.33	39.98*

b

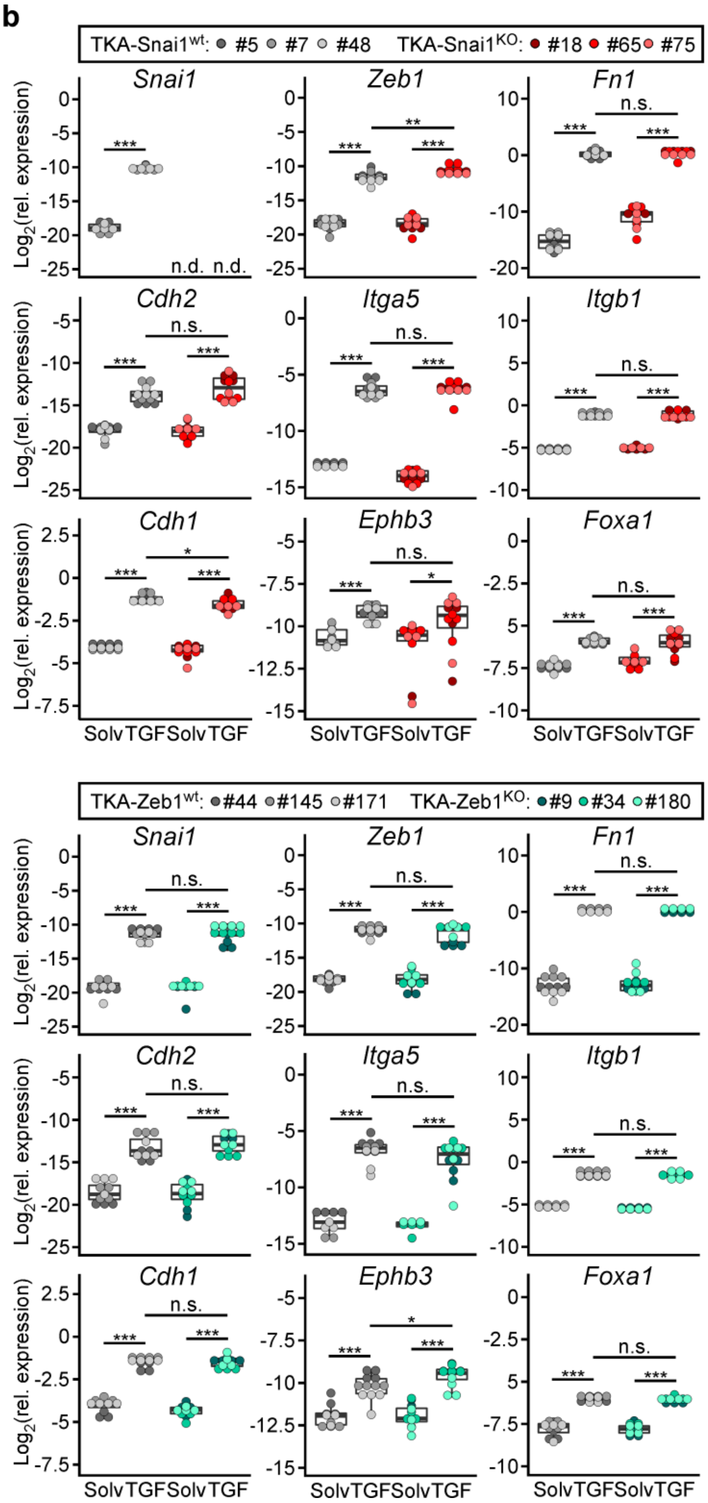

c

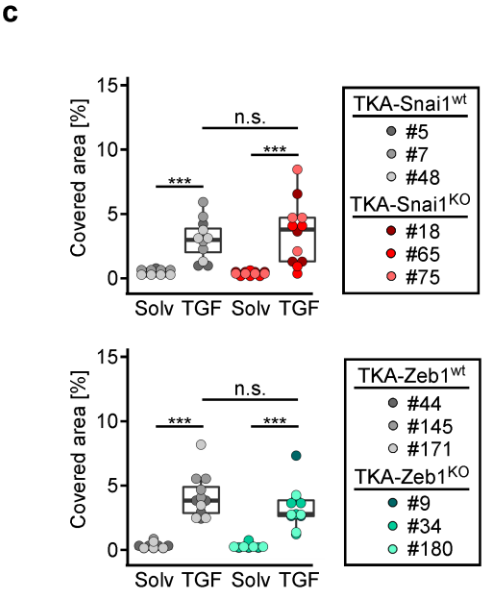

Supplementary figure 15: Snail1 and Zeb1 are not required for the TGFβ1-induced partial EMT of oncogenically transformed organoids. a, Expression analysis of EMT-TFs by qRT-PCR in TKA organoids treated with solvent or TGFβ1 for the indicated periods of time. Presented are mean Ct-values calculated from technical duplicates of one representative experiment. N.t.: no template control, n.d.: no detection, *: value from only one of the two technical replicates is shown because the other had no detectable fluorescence signal after 40 cycles of amplification. **b**, Gene expression analysis of EMT-TFs and EMT-associated genes in TKA-Snai1^{wt}, TKA-Snai1^{KO}, TKA-Zeb1^{wt}, and TKA-Zeb1^{KO} organoid lines stimulated with solvent (solv) or TGFβ1 (TGF) for 72 h. Gene-specific transcripts were quantified by qRT-PCR

and normalized to transcript levels of *Eef1a1* (n=4). Each dot represents the result of a single measurement while dot color identifies the organoid lines; n.d.: not detectable. ***$p<0.001$**,** **$p<0.01$, *$p<0.05$, n.s.: not significant; statistical significance was analyzed using the Mann-Whitney *U* test. Exact *p*-values are provided in Supplementary table 7. **c**, Invasiveness of TKA-Snai1^{wt}, TKA-Snai1^{KO}, TKA-Zeb1^{wt}, and TKA-Zeb1^{KO} organoid lines treated with solvent or TGFβ1 for 96 h was quantified using Boyden chamber assays. Each dot represents the result of a single invasion assay while dot color identifies the organoid lines (n=4). ***$p<0.0001$, n.s., not significant: $p=0.7508$ (TGFβ1-treated TKA-Snai1^{wt} versus TKA-Snai1^{KO}) and $p=0.1939$ (TGFβ1-treated TKA-Zeb1^{wt} versus TKA-Zeb1^{KO}); statistical significance was analyzed using the Mann-Whitney *U* test.

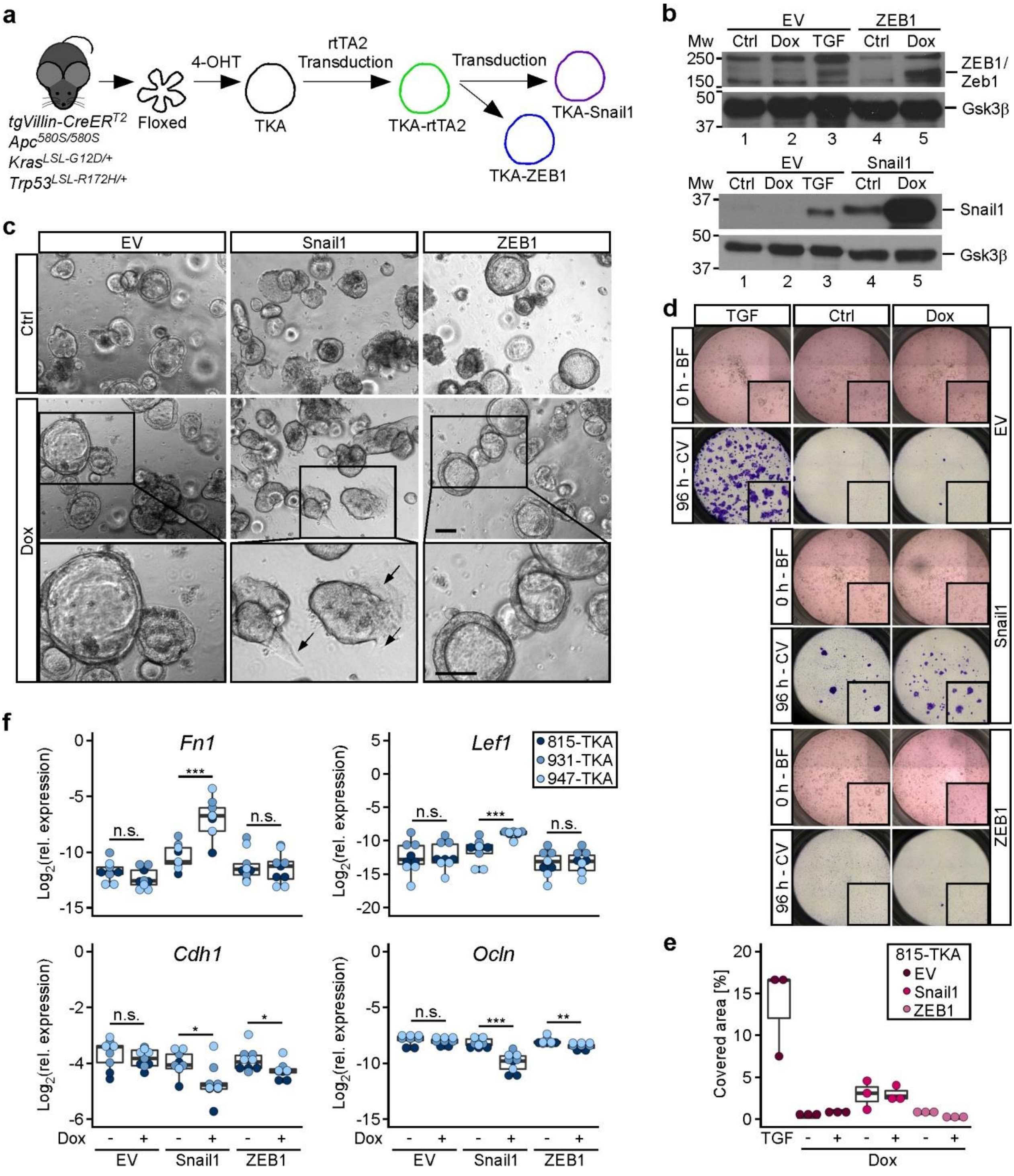

Supplementary figure 16: Overexpression of Snail1 and ZEB1 does not mimic the TGFβ1-induced phenotype of oncogenically transformed organoids. a, Strategy for the generation of TKA organoids with doxycycline (Dox)-inducible overexpression of murine Snail1 and human ZEB1. TKA organoids were transduced with a retroviral vector expressing the reverse tetracycline-dependent trans-activator (rtTA2), followed by a second transduction with an empty expression vector and retroviral constructs for Dox-inducible overexpression of Snail1 and ZEB1. **b**, Western blot analyses to examine Snail1 and ZEB1 transgene expression in TKA organoids (line 815) left untreated (ctrl) or stimulated with Dox for 72 h. Gsk3β detection served as loading control. Results are representative for two independent biological replicates performed with derivatives of three different TKA organoid lines (815: n=2; 931: n=2; 947: n=2).

Molecular weights of size standards are given in kDa. **c**, Phase contrast microscopic pictures of transduced TKA organoids (line 815) left untreated (ctrl) or stimulated with Dox for 72 h. Boxed areas are shown at higher magnification below. Arrows highlight sites of invasive behavior. Scale bars: 100 µm. **d**, Boyden chamber invasion assays with TKA organoids (line 815) transduced with empty vector (EV) and Dox-inducible expression vectors for Snail1 and ZEB1. Bright field (BF) images taken at the onset of the experiment (0 h) of untreated organoids (ctrl) and organoids receiving Dox. Empty vector-transduced organoids were treated with TGFβ1 for comparison. Inserts show magnified views of the upper chambers. Invaded cells were visualized by crystal violet (CV) staining after 96 h of treatment. Inserts show magnified views of the bottom faces of the Boyden chambers. **e**, Quantification of invasion experiments as shown in (**d**) performed with TKA organoids derived from line 815 and stimulated with TGFβ1 or cultured in the absence (-) or presence (+) of Dox for 96 h (n=3). Dots represent results of individual experiments. Dot color identifies the organoid lines. Statistical analysis was not appropriate due to low sample size. **f**, Gene expression analysis of *Fn1*, *Lef1*, *Cdh1*, and *Ocln* in TKA organoids transduced with empty vector (EV) and Dox-inducible expression vectors for Snail1 and ZEB1. Organoids were cultured in the absence (-) or presence (+) of Dox for 72 h. Gene-specific transcripts were quantified by qRT-PCR and normalized to transcript levels of *Eef1a1*. Dots represent results of individual experiments while dot color identifies the organoid lines. Three independent biological replicates each were performed with derivatives of three different TKA organoid lines (815: n=3; 931: n=3; 947: n=3). ***p<0.001, **p<0.01, *p<0.05, n.s.: not significant; Mann-Whitney *U* test. Exact p-values are provided in Supplementary table 7.

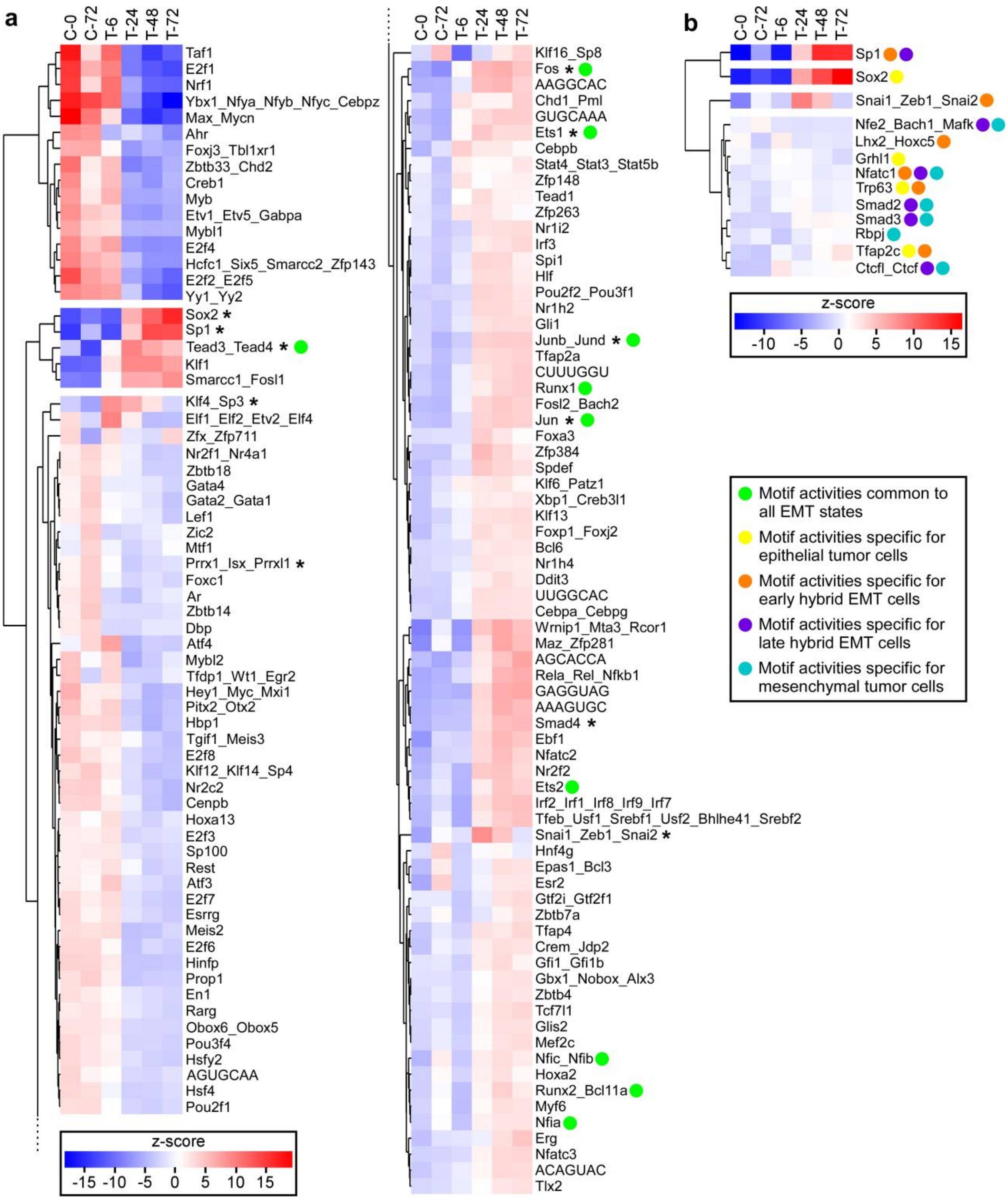

Supplementary figure 17: Dynamics of motif activity in the course of TGFβ1-induced pEMT of TKA organoids. ISMARA (Integrated System for Motif Activity Response Analysis) (2) was used to identify potential regulatory DNA sequence motifs and TFs, whose activity might drive pEMT of TGFβ1-treated TKA organoids. ISMARA was carried out with the RNA-seq data for TKA organoids (lines 931 and 947) harvested immediately at the onset of the experiment (0 h of cultivation; C-0), cultivated for 72 h in solvent (C-72), and treated with TGFβ1 for the indicated periods of time. **a**, The activity (presented as z-score) of motifs with an overall z-value>2 (a measure for the contribution of each motif to the observed transcriptional changes) were plotted in a heatmap. Some factors with increased motif activity (marked by an asterisk) were previously associated with EMT, for example, Sox proteins, Sp1,

Krüppel-like factors, Tead3/4, Snail1, Snail2, Zeb1, Fos and Jun family members, Smad4, and the Ets TF family (3, 4). The activities of TF motifs which have been described to operate across the entire spectrum of EMT stages (4) are highlighted by a green dot. **b**, The activity (presented as z-score) of TF motifs reported to be functional in distinct EMT states in squamous cell carcinoma and breast cancer (4). Colored dots indicate the TF motifs of the different EMT states, including epithelial (yellow), early hybrid EMT (orange), late hybrid EMT (purple), and mesenchymal (turquoise) tumor cell states. Low changes in activity for the majority of TF motifs argue that alternative sets of TFs drive pEMT in TKA organoids.

Legend for Supplementary movie 1 (separate file):

TGFβ1-induced collective invasion of oncogenically transformed small intestinal organoids. Live imaging of small intestinal TKA organoids (line 815) treated with solvent or TGFβ1 for 72 h. Images were acquired at 2 h intervals for a total of 72 h.

Supplementary Methods

Treatment of organoids with TGFβ1 and small molecule inhibitors. For experiments involving TGFβ signaling, organoids were mechanically disrupted and seeded 30-40 h before treatments were started. To activate and inhibit TGFβ signaling, 5 ng/ml human TGFβ1 (#100-21, Peprotech, Rocky Hill, New Jersey, USA) and 10 µM SB431542 (#S1067, Selleckchem, Houston, Texas, USA) were administered, respectively. To inhibit PI3K and MEK1/2, organoids were treated with 3 µM Buparlisib (#S2247, Selleckchem, Houston, Texas, USA) and 30 nM Trametinib (#S2673, Selleckchem), respectively. To assess independence from EGFR signaling, organoids were mechanically disrupted and seeded in Matrigel two days before treatment with 0.8 µM Gefitinib (#S1025, Selleckchem) for 72 h. Organoid viability was then determined by phase contrast microscopy and subsequent incubation with 500 µg/ml MTT (3-(4,5-dimethylthiazol-2-yl)-2,5-diphenyltetrazolium bromide) at 37°C for 1 h. Culture plates were imaged with a CanoScan 9950F scanner (Canon, Tokyo, Japan). Culture media supplemented with growth factors, inhibitors and other reagents were refreshed every 48 h.

Assessment of epithelial integrity. To test for epithelial integrity, organoids were mechanically disrupted and seeded in Matrigel two days before treatment with 5 µM forskolin (#S2449, Selleckchem). Swelling was monitored for 8 h at 20 min intervals using the JuLI™ Stage Real-Time Cell History Recorder (NanoEntek, Seoul, Korea) and a 10x objective.

Assessment of organoid-forming capacity. TKA organoids treated with solvent or TGFβ1 for 72 h were dissociated into single cells by incubation with Accutase (#SCR005, Merck; Darmstadt, Germany) at 37°C for 10 min. A total number of 5 000, 10 000, and 50 000 cells was seeded per well of 24-well plates in 50 µl Matrigel (3 mg/ml). Culture medium was supplemented by Y-27632 (#S1049, Selleckchem) and treatment with solvent or TGFβ1 was continued for another 72 h before organoids were imaged by bright field microscopy and stained by MTT reagent.

Boyden chamber invasion assay. Organoids were mechanically disrupted and seeded into inserts for 24-well plates (#353097, Corning Life Sciences, Corning, New York, USA) with Matrigel 30-40 h prior to treatments as described in the figure legends. Invaded cells on the bottom surface of the membranes were fixed with 4% paraformaldehyde at room temperature (RT) for 10 min and stained with 0.1% (w/v) crystal violet in water at RT for 10 min. For quantification, membranes were imaged using the BZ-9000 fluorescence microscope (Keyence, Osaka, Japan) and the proportion of the membranes covered with invasive cells was determined using ImageJ.

Air-liquid interface (ALI) culture. For ALI cultures (5), organoids were incubated in Cell Recovery Solution (#354253, Corning) on ice for 1 h, washed in PBS, mechanically disrupted, and seeded in type I collagen into 24-well transwell filter inserts. After gelation, culture medium was added to the outer well. Medium was exchanged every three days. After ten days, cultures were fixed in 4% paraformaldehyde at RT overnight, paraffin-embedded, sectioned into 5 µm slices, stained with hematoxylin and eosin, and imaged using the BZ-9000 fluorescence microscope (Keyence).

Culture in type I collagen. Type I collagen (Collagen I HC, rat tail, #354249, Corning) was diluted with cold PBS to a concentration of 3 mg/ml. 1/20 (v/v) Medium 199 (#M0650, Sigma Aldrich, Taufkirchen, Germany) was added, the pH was adjusted with NaOH, and the matrix was incubated on ice for 1 h. Organoids were prepared as described for ALI cultures, resuspended in the collagen matrix, and seeded on pre-warmed plates. After gelation, culture medium was added. Two days later, treatment with solvent or TGFβ1 was started.

Genome editing. *Itgav, Smad2, Smad3, Smad4*, *Snai1*, and *Zeb1* were inactivated by frame-shift-inducing exon deletions. For this, suitable exons were targeted by two single guide RNAs (sgRNAs; target sites listed in Supplementary table 3) selected using CCTop (6). Expression cassettes for sgRNAs were generated using vectors from the MuLE system (Supplementary table 4) (7). For *Smad4* deletion, 400 ng of each sgRNA expression plasmid and 700 ng pCAG-Cas9-turbo-RFP vector were transfected into floxed organoids as described (8), except that single cell suspensions were generated with Accutase and transfection was done with Lipofectamine® LTX (#15338100, Thermo Fisher Scientific). Three days after transfection, *Smad4*-deficient organoids were selected for by adding 100 ng/ml murine BMP-4 (#315-27, Peprotech) to the culture media while omitting Noggin. *Smad4*-deficient organoids were treated with 4-OHT to generate quadruple mutant TKAS organoids. To inactivate *Snai1*, floxed organoids were transduced with pLenti-Cas9-T2A-BlastR and selected with blasticidin. Cas9-expressing organoids were transduced with pLenti-Dest-Snai1-sgRNA1+2-eGFP-F2A-NeoR-loxP and selected with G418 for one week. Thereafter, 0.5 µM 4-OHT was administered for 5 days to induce excision of loxP-flanked DNA sequences including the sgRNA-expressing provirus. Organoids were dissociated and sparsely seeded to facilitate clonal outgrowth. Single cell-derived organoids were manually picked, expanded, and screened by PCR with primers listed in Supplementary table 3. *Itgav*, *Smad2*, *Smad3*, and *Zeb1* were inactivated similarly, except that TKA organoids were simultaneously transduced with pLenti-Cas9-T2A-BlastR and gene-specific sgRNA expression vectors listed in Supplementary table 4. To select for organoids simultaneously transduced with viral constructs expressing Cas9 and sgRNA, they

were concomitantly treated with blasticidin and G418. *Snai1*^{WT}*, Snai1*^{KO}*, Zeb1*^{WT}*, Zeb1*^{KO}, *Itgav* exon 4$^{+/+}$ and *Itgav* exon 4$^{-/-}$ TKA organoids were additionally exposed to 4-OHT for 3 days to excise the sgRNA-expressing proviral genome.

Viral transduction. Lentiviral and retroviral particles were produced by co-transfecting HEK293T cells (not authenticated but tested negative for mycoplasma contamination) with viral vectors listed in Supplementary table 4 and packaging plasmids at a mass ratio of 1:0.75:0.3 using FuGENE®6 (#E2691, Promega, Fitchburg, Wisconsin, USA). Virus-containing medium was filtrated (0.45 µm) 48 h post-transfection and centrifuged at 8 000*g* and 4°C overnight. Viral pellets were resuspended in culture medium supplemented with 8 µg/ml Polybrene (#TR-1003-G, Merck, Burlington, Massachusetts, USA) and 10 µM Y-27632. For infection, organoids were dissociated into single cells by incubation with Accutase at 37°C for 10 min, mixed with the viral suspensions, plated on a Matrigel bed, and incubated at 37°C for 8-9 h. After washing with PBS, attached cells were overlaid with Matrigel and provided with culture medium containing Y-27632. Floxed organoids additionally received 1 µM valproic acid (Sigma Aldrich, P4543) and 1 µM CT99021 (#S2924, Selleckchem). Selection with antibiotics was started three days (floxed organoids) and two days (TKA organoids) after transduction, using 5 ng/ml blasticidin (#R210-01, Thermo Fisher Scientific, Waltham, Massachusetts, USA), 2 µg/ml puromycin (#P7255, Sigma Aldrich), 500 µg/ml geneticin (G418; #10131027, Thermo Fisher Scientific) (floxed organoids), and 700 µg/ml G418 (TKA organoids). For Dox-inducible gene expression, organoids were transduced with pMSCV-rtTA2-PGK-eGFP-F2A-NeoR, followed by a second infection with pRetroX-tight-Snail1-HA-PuroR, pRetroX-tight-ZEB1-HA-PuroR, or pRetroX-tight-MCS-PuroR (9, 10). Expression of Snail1 and ZEB1 was induced with 1 µg/ml Dox (Sigma Aldrich, D9891). For 4-OHT-inducible gene expression, TKA organoids were transduced with pMSCV-loxP-BlastR-loxP-TGFBR1(T204D)-F2A-eGFP (coding for TGFBR1CA), pMSCV-loxP-BlastR-loxP-TGFBR2(Δcyt)-F2A-eGFP (coding for TGFBR1DN), and pMSCV-loxP-BlastR-loxP-eGFP. Expression of TGFβ receptor mutants was induced by treating organoids with 0.5 µM 4-OHT. In case of TGFBR2DN, this was done three days prior to the start of invasion and gene expression experiments. For fluorescent labeling and live cell imaging, TKA organoids were transduced with pLenti-SV40-mTomato-P2A-H2B-GFP-PuroR. All constructs used in the study and their parental vectors are listed in Supplementary table 4.

Picrosirius red staining and microscopy. After 96 h of solvent or TGFβ1 treatment, organoid cultures in type I collagen were fixed in 10% formalin at RT overnight, paraffin embedded, and sectioned into 40 µm slices. Picrosirius red staining was performed as described (11). Sections were imaged using an Axioplan2 fluorescence microscope (Zeiss, Oberkochen, Germany) equipped with a polarizer, an analyzer, and an Axiocam camera. All pictures taken with

polarized or non-polarized light were acquired with the same exposure times. To quantify the formation of parallel and larger collagen bundles, images were acquired under polarizing light with a 540 nm filter. After acquisition, picrosirius red-derived signals above a defined threshold were measured within a 65 µm wide area immediately surrounding an organoid under investigation, while excluding the organoid itself.

Immunofluorescence staining of paraffin sections and whole mounts. For sectioning, organoids were fixed in 4% paraformaldehyde overnight at 3-4 days after seeding, paraffin-embedded, and sectioned into 5 µm slices. Sections were stained as described (12). For whole mount imaging, organoids were seeded into chambered coverslips (#80826, Ibidi, Gräfelfing, Germany), treated with solvent or TGFβ1 for 72 h, and fixed *in situ* with 4% paraformaldehyde at 4°C for 45 min. After permeabilization with 0.5% (v/v) Triton X-100 in PBS at 4°C for 10 min and quenching with 0.1 M glycine in PBS at RT for 15 min, samples were incubated with blocking buffer (PBS with 10% FCS, 0.2% Triton X-100, 0.05% Tween20, 0.1% [w/v] BSA) at RT for 1 h. Primary antibodies were diluted in blocking buffer and incubated at 4°C overnight. After washing with blocking buffer, organoids were incubated with 0.3 µM DAPI and secondary antibodies diluted in blocking buffer. For actin staining, 5 u ml^{-1} phalloidin CF555 (#00040, Biotium, Fremont, California, USA) were added together with the secondary antibodies. After washing with blocking buffer, organoids were mounted with 0.1% (w/v) n-propyl gallate (#P3130, Sigma Aldrich) in PBS. Primary and secondary antibodies are listed in Supplementary table 5.

Fluorescence microscopy. Images of immunofluorescence stainings of paraffin sections were acquired using an Axio Observer.Z1 fluorescence microscope with an ApoTome2 equipment (Zeiss). Whole mount immunofluorescence stainings were imaged with a LSM 880 confocal microscope (Zeiss) with an Achroplan IR 40x/0.8 W objective and laser wavelengths of 405, 488, and 561 nm, unless stated differently. From image stacks (1.04 µm step size) orthogonal views of cross sections were generated using ZEN 2.3 and ImageJ. For live imaging, TKA organoids expressing mTomato and H2B-GFP were seeded into chambered culture plates (Ibidi, 80416). During confocal microscopy (settings as above), organoids were kept at 37°C and 5% CO_2 in a Tokai Hit Incubator. Orthogonal views of cross sections and 3D reconstructions from live imaging data were generated using Huygens Professional for deconvolution and Imaris.

RNA isolation, qRT-PCR, and RNA-seq. RNA was isolated and cDNA was synthesized with the peqGOLD MicroSpin Total RNA Kit (#12-6831, VWR International GmbH, Bruchsal, Germany) and the qScript™ Flex cDNA Kit (#95049, Quantabio, Beverly, Massachusetts,

USA), respectively. For qRT-PCR, the PerfeCTa® SYBR® Green SuperMix (#95049, Quantabio) was employed (primers listed in Supplementary table 6) with amounts of cDNA equivalent to 10 ng and 20 ng RNA when PCRs were conducted in a CFX384 and CFX96 Touch Real-Time PCR Detection System, respectively, (Bio-Rad Laboratories, Hercules, California, USA). Following normalization to *Gapdh* or *Eef1a1* transcripts, gene expression data were logarithmically transformed to yield normally distributed values, and are presented as $\log_2(2^{-\Delta CT})$. For global transcriptome analysis, RNA was collected from TGFβ1-treated organoids after 6, 24, 48, and 72 h, and from solvent-treated controls after 0 and 72 h of cultivation and paired-end sequenced on an Illumina HiSeq4000 at the Genome and Proteome Core Facility of the German Cancer Research Center, Heidelberg, Germany. FASTQ files were trimmed for sequencing adapters and low-quality reads with Trimmomatic (13). Reads were aligned to the Ensembl genome GRCM38 and reads per gene were quantified using STAR (14). For statistical analyses the R/Bioconductor package edgeR was used (15). We matched Ensembl IDs with EntrezIDs. If multiple Ensembl IDs matched to more than one Entrez ID, the one with the largest inter-quartile-range across samples was kept. Genes with less than one count per million in at least 3 samples were filtered out. Data were normalized with TMM (trimmed mean of M-values). Tagwise dispersion was calculated using edgeR. EdgeR was also used to compare data of the time series of TGFβ1 treatment to the 0 h and 72 h solvent controls. Differentially expressed genes (DEG) were defined by an adj. *p*-value <0.01 and a $|\log_2(FC)|>1$. Fisher's exact test for Gene Ontology biological processes (GO:BP) (16), ConsensusPathDB (17), a selected group of EMT signatures (18–22) and the Lgr5 intestinal stem cell signature (23) was applied for functional enrichment analysis of DEGs. Terms were considered to be significantly regulated at an adj. *p*-value <0.05.

Protein expression analysis. Organoids were incubated in Cell Recovery Solution and nuclear extracts were prepared by resuspending organoids in nuclear extraction buffer A (10 mM HEPES/KOH pH 7.9, 0.1 mM EDTA, 10 mM KCl, 1x Complete® [1697498, Roche, Mannheim, Germany], 1 mM DTT, 1x phosphatase inhibitor cocktails 2 and 3 [Sigma Aldrich, P5726/P0044]). After incubation on ice for 15 min, 0.5% (v/v) NP-40 was added, organoid suspensions were shortly vortexed, and nuclei were pelleted by centrifugation at 4°C and 16 100*g* for 2 min. The cytosolic supernatant was collected, and the nuclear pellet was washed once with nuclear extraction buffer A. Thereafter, nuclei were resuspended in 20 mM HEPES/KOH pH 7.9, 400 mM NaCl, 1 mM EDTA, 1x Complete®, 1 mM DTT, 1x phosphatase inhibitor cocktails 2 and 3, and incubated at 4°C for 30 min with constant shaking. Nuclear extracts were cleared by centrifugation at 4°C and 16 100*g* for 10 min. Protein concentrations were determined with the BioRad DC™ Protein Assay (#500-0113, BioRad, Feldkirchen, Germany). Depending on the protein yield in a given experiment, 25-40 µg of protein was

separated by SDS-PAGE and transferred to nitrocellulose for protein detection as described (24). Antibodies are listed in Supplementary table 5.

Analysis of colon cancer data. Colon cancer RNA-seq data were downloaded from TCGA firehose (https://gdac.broadinstitute.org). Following CMS classification, only CMS2 and CMS4 samples were further processed. For these, we matched Ensembl IDs with Entrez IDs and, if multiple Ensembl IDs were matched to more than one Entrez ID, the one with the largest inter-quartile-range across samples was kept. Genes with less than one count per million in at least 3 samples were filtered out. TMM normalization, tagwise dispersion, and statistical analysis using edgeR were performed similarly to described above. For comparison of CMS4 to CMS2 samples, DEGs were defined by an adj. p-value <0.01 and $|\log_2(FC)|>1$ as calculated with edgeR. DEGs were subjected to functional enrichment analysis using Fisher's exact test for the selected group of previously defined EMT signatures. A signature was considered to be significantly regulated if the adj. p-value <0.05.

SI References:

1. Xiong J-P, Stehle T, Zhang R, Joachimiak A, Frech M, Goodman SL et al. Crystal structure of the extracellular segment of integrin alpha Vbeta3 in complex with an Arg-Gly-Asp ligand. Science 2002; 296:151–5.
2. Balwierz PJ, Pachkov M, Arnold P, Gruber AJ, Zavolan M, van Nimwegen E. ISMARA: automated modeling of genomic signals as a democracy of regulatory motifs. Genome Res 2014; 24:869–84.
3. Dongre A, Weinberg RA. New insights into the mechanisms of epithelial-mesenchymal transition and implications for cancer. Nat Rev Mol Cell Biol 2019; 20:69–84.
4. Pastushenko I, Blanpain C. EMT Transition States during Tumor Progression and Metastasis. Trends Cell Biol 2019; 29:212–26.
5. Li X, Nadauld L, Ootani A, Corney DC, Pai RK, Gevaert O et al. Oncogenic transformation of diverse gastrointestinal tissues in primary organoid culture. Nat Med 2014; 20:769–77.
6. Stemmer M, Thumberger T, Del Sol Keyer M, Wittbrodt J, Mateo JL. CCTop: An Intuitive, Flexible and Reliable CRISPR/Cas9 Target Prediction Tool. PLoS ONE 2015; 10:e0124633.
7. Albers J, Danzer C, Rechsteiner M, Lehmann H, Brandt LP, Hejhal T et al. A versatile modular vector system for rapid combinatorial mammalian genetics. J Clin Invest 2015; 125:1603–19.
8. Drost J, van Jaarsveld RH, Ponsioen B, Zimberlin C, van Boxtel R, Buijs A et al. Sequential cancer mutations in cultured human intestinal stem cells. Nature 2015; 521:43–7.
9. Rönsch K, Jägle S, Rose K, Seidl M, Baumgartner F, Freihen V et al. SNAIL1 combines competitive displacement of ASCL2 and epigenetic mechanisms to rapidly silence the EPHB3 tumor suppressor in colorectal cancer. Mol Oncol 2015; 9:335–54.
10. Schnappauf O, Beyes S, Dertmann A, Freihen V, Frey P, Jägle S et al. Enhancer decommissioning by Snail1-induced competitive displacement of TCF7L2 and down-regulation of transcriptional activators results in EPHB2 silencing. Biochim Biophys Acta 2016; 1859:1353–67.
11. Nyström A, Thriene K, Mittapalli V, Kern JS, Kiritsi D, Dengjel J et al. Losartan ameliorates dystrophic epidermolysis bullosa and uncovers new disease mechanisms. EMBO Mol Med 2015; 7:1211–28.
12. Freihen V, Rönsch K, Mastroianni J, Frey P, Rose K, Boerries M et al. SNAIL1 employs β-Catenin-LEF1 complexes to control colorectal cancer cell invasion and proliferation. Int J Cancer 2020; 146:2229–42.
13. Bolger AM, Lohse M, Usadel B. Trimmomatic: a flexible trimmer for Illumina sequence data. Bioinformatics 2014; 30:2114–20.
14. Dobin A, Davis CA, Schlesinger F, Drenkow J, Zaleski C, Jha S et al. STAR: ultrafast universal RNA-seq aligner. Bioinformatics 2013; 29:15–21.
15. Robinson MD, McCarthy DJ, Smyth GK. edgeR: a Bioconductor package for differential expression analysis of digital gene expression data. Bioinformatics 2010; 26:139–40.
16. Subramanian A, Tamayo P, Mootha VK, Mukherjee S, Ebert BL, Gillette MA et al. Gene set enrichment analysis: a knowledge-based approach for interpreting genome-wide expression profiles. Proc Natl Acad Sci U S A 2005; 102:15545–50.
17. Kamburov A, Wierling C, Lehrach H, Herwig R. ConsensusPathDB--a database for integrating human functional interaction networks. Nucleic Acids Res 2009; 37:D623-8. Available from: URL: https://pubmed.ncbi.nlm.nih.gov/18940869/.
18. Gotzmann J, Fischer ANM, Zojer M, Mikula M, Proell V, Huber H et al. A crucial function of PDGF in TGF-beta-mediated cancer progression of hepatocytes. Oncogene 2006; 25:3170–85.

19. Gröger CJ, Grubinger M, Waldhör T, Vierlinger K, Mikulits W. Meta-analysis of gene expression signatures defining the epithelial to mesenchymal transition during cancer progression. PLoS ONE 2012; 7:e51136.
20. Jechlinger M, Grunert S, Tamir IH, Janda E, Lüdemann S, Waerner T et al. Expression profiling of epithelial plasticity in tumor progression. Oncogene 2003; 22:7155–69.
21. Mak MP, Tong P, Diao L, Cardnell RJ, Gibbons DL, William WN et al. A Patient-Derived, Pan-Cancer EMT Signature Identifies Global Molecular Alterations and Immune Target Enrichment Following Epithelial-to-Mesenchymal Transition. Clin Cancer Res 2016; 22:609–20.
22. Taube JH, Herschkowitz JI, Komurov K, Zhou AY, Gupta S, Yang J et al. Core epithelial-to-mesenchymal transition interactome gene-expression signature is associated with claudin-low and metaplastic breast cancer subtypes. Proc Natl Acad Sci U S A 2010; 107:15449–54.
23. Munoz J, Stange DE, Schepers AG, van de Wetering M, Koo B-K, Itzkovitz S et al. The Lgr5 intestinal stem cell signature: Robust expression of proposed quiescent '+4' cell markers. EMBO J 2012; 31:3079–91.
24. Weise A, Bruser K, Elfert S, Wallmen B, Wittel Y, Wöhrle S et al. Alternative splicing of Tcf7l2 transcripts generates protein variants with differential promoter-binding and transcriptional activation properties at Wnt/beta-catenin targets. Nucleic Acids Res 2010; 38:1964–81.
