# Supplementary material for: Canonical TGFβ signaling induces collective invasion in colorectal carcinogenesis through a Snail1- and Zeb1-independent partial EMT
Source: Oncogene. 2022 Jan 24;41(10):1492–506. doi: 10.1038/s41388-022-02190-4 (PMC8897192; doi:10.1038/s41388-022-02190-4)
Supplement: Supplementary file 5 — Supplementary table 3_Flum et al [file 41388_2022_2190_MOESM5_ESM.docx]

**Supplementary table 3: DNA sequences of sgRNA targets and oligonucleotides employed in genome editing experiments (5’-3’)**

| **sgRNA target sequences excluding PAM** | |
| --- | --- |
| Itgav-sgRNA1 | CCTGTGATAAAGCAGAGGCT |
| Itgav-sgRNA2 | CCAGGCCTGATCTAGTGACT |
| Smad2-sgRNA1 | TGAGTGTGGATTGTTACCTT |
| Smad2-sgRNA2 | GCACTTCATCTGCACAGAGT |
| Smad3-sgRNA1 | CCTGGGGAGTTCAAGGCTGC |
| Smad3-sgRNA2 | TCCCTGCCGGGAATGATCAG |
| Smad4-sgRNA1 | ACTAATACCTTGACACTCTA |
| Smad4-sgRNA2 | GTTTTCAGTGGCTATTGATT |
| Snai1-sgRNA1 | GGTAGTCAACTCCGCTCGCG |
| Snai1-sgRNA2 | GTGTGGGTTGAGCCCGGATA |
| Zeb1-sgRNA1 | GATCTAGGCCTGCCATTCAC |
| Zeb1-sgRNA2 | TTATGAGTTCAAACCCATAG |
| **PCR primers for genotyping** | |
| Itgav P1 | CAAGAGCTCTGCTGACTGCT |
| Itgav P2 | CAGACACGCAGGCTTTTCAC |
| Smad2 P1 | GTTGTGTGCTGTGGTTTGCA |
| Smad2 P2 | gatggaccaaggcgaaagga |
| Smad3 P1 | tctcttccagcctaggggac |
| Smad3 P2 | ctgcctcaactcccactctg |
| Smad4 P1 | CCCTTCTCCCCACCCTGATA |
| Smad4 P2 | TGCCTATGTGCAACCTCAGG |
| Snai1 P1 | AGACAGTTCCAGGAACCCCT |
| Snai1 P2 | GCTGAAGCCTTCCCTCACTT |
| Zeb1 P1 | AAGCCATACGAATGCCCGAA |
| Zeb1 P2 | TCGGCGATCTTTGAGAGCTC |
| Apc-A1 | GTTCTGTATCATGGAAAGATAGGTGGTC |
| Apc-A2 | GAGTACGGGGTCTCTGTCTCAGTGAA |
| Apc-A3 | CACTCAAAACGCTTTTGAGGGTTG |
| Kras-K1 | GTCTTTCCCCAGCACAGTGC |
| Kras-K2 | CTCTTGCCTACGCCACCAGCTC |
| Kras-K3 | AGCTAGCCACCATGGCTTGAGTAAGTCTGCA |
| Trp53-T1 | AGCCTTAGACATAACACACGAACT |
| Trp53-T2 | CTTGGAGACATAGCCACACTG |
| Trp53-T3 | GCCACCATGGCTTGAGTAA |
| **PCR primer for amplification of Itgav exons 1-7 from cDNA** | |
| Itgav-201 ex1 | CCCGAGGGAAGTTACTTCGG |
| Itgav-201 ex7 | TTCTGCCACTTGGTCCGAAA |
