# Supplementary material for: Canonical TGFβ signaling induces collective invasion in colorectal carcinogenesis through a Snail1- and Zeb1-independent partial EMT
Source: Oncogene. 2022 Jan 24;41(10):1492–506. doi: 10.1038/s41388-022-02190-4 (PMC8897192; doi:10.1038/s41388-022-02190-4)
Supplement: Supplementary file 7 — Supplementary table 5_Flum et al [file 41388_2022_2190_MOESM7_ESM.docx]

**Supplementary table 5: List of antibodies used for immunofluorescence staining and Western blotting**

| **Primary antibodies for immunofluorescence staining** | | |
| --- | --- | --- |
| **Antigen** | **Type, origin, dilution** | **Catalogue number, clone number*, supplier** |
| β-Catenin | polyclonal, rabbit, 1:50 | #9581, Cell Signaling Technology, Danvers, Massachusetts, USA |
| CD61 (integrin β3) | monoclonal, rabbit, 1:100 | #MA5-32077, clone SJ19-09, Thermo Fisher Scientific, Waltham, Massachusetts, USA |
| Claudin-7 | polyclonal, rabbit, 1:100 | #34-9100, Thermo Fisher Scientific |
| Cleaved caspase-3 (Asp175) | polyclonal, rabbit, 1:400 | #9661, Cell Signaling Technology |
| E-cadherin | monoclonal, mouse, 1:200 | #610182, clone 36/E-Cadherin, BD Biosciences, San Jose, California, USA |
| Fibronectin | polyclonal, rabbit, 1:400 | #ab2413, Abcam, Cambridge, UK |
| Ki67 | monoclonal, rabbit, 1:400 | #9129, clone D3B5, Cell Signaling Technology |
| Laminin | polyclonal, rabbit, 1:25 | #L9393, Sigma Aldrich |
| PKC ζ (atypical PKC isoform) | monoclonal, mouse, 1:100 | #sc-17781, clone H-1, Santa Cruz |
| Smad2/3 | monoclonal, rabbit, 1:800 | #8685, clone D7G7, Cell Signaling Technology |
| Vinculin | monoclonal, mouse, 1:400 | #V9131, clone hVIN-1, Sigma Aldrich |
| **Secondary antibodies for immunofluorescence staining** | | |
| **Antigen** | **Origin, fluorophore, dilution** | **Catalogue number, supplier** |
| Mouse IgG | donkey, Alexa Fluor555-conjugated, 1:500 | #A-31570, Thermo Fisher Scientific |
| Rabbit IgG | goat, Alexa Fluor488-conjugated, 1:200 | #A-11008, Thermo Fisher Scientific |
| **Primary antibodies for Western blotting** | | |
| **Antigen** | **Type, origin, dilution** | **Supplier, catalogue number, clone number*** |
| α-Tubulin | monoclonal, mouse, 1:10000 | #T9026, clone DM1A, Sigma Aldrich |
| Phospho-AKT  (Ser473) | polyclonal, rabbit, 1:1000 | #9271, Cell Signaling Technology |
| AKT | polyclonal, rabbit, 1:1000 | #9272, Cell Signaling Technology |
| Cleaved caspase-3 (Asp175) | polyclonal, rabbit, 1:1000 | #9661, Cell Signaling Technology |
| E-cadherin | monoclonal, mouse, 1:1000 | #610404, BD Biosciences |
| Ephb3 | monoclonal, mouse, 1:5000 | #H00002049-M01, clone 3F12, Abnova, Taipei, Taiwan |
| Phospho-ERK1/2  (Thr202/Tyr204) | monoclonal, mouse, 1:1000 | #9106, clone E10, Cell Signaling Technology |
| ERK1/2 | polyclonal, rabbit, 1:1000 | #9102, Cell Signaling Technology |
| Fibronectin | polyclonal, rabbit, 1:1000 | #ab2413, Abcam |
| Gsk3β | monoclonal, mouse, 1:1000 | #610201, clone 7/GSK-3β, BD Biosciences |
| Integrin α5 | polyclonal, rabbit, 1:1000 | #4705, Cell Signaling Technology |
| CD51 (integrin αV) | monoclonal, rabbit, 1:1000 | #60896, clone D2N5H, Cell Signaling Technology |
| Integrin β1 | monoclonal, rabbit, 1:1000 | #34971, clone D6S1W, Cell Signaling Technology |
| Phospho-Smad2 (Ser465/467)/Smad3 (Ser423/425) | monoclonal, rabbit, 1:1000 | #8828, clone D27F4, Cell Signaling Technology |
| Smad2/3 | monoclonal, rabbit, 1:5000 | #8685, clone D7G7, Cell Signaling Technology |
| Smad4 | monoclonal, mouse, 1:2000 | #sc-7966, clone D7G7, Santa Cruz, Santa Cruz, California, USA |
| Snail1 | monoclonal, rabbit, 1:1000 | #3879, clone C15D3, Cell Signaling Technology |
| Zeb1 | polyclonal, rabbit, 1:1000 | #HPA027524, Sigma Aldrich |
| Zeb1^#^ | monoclonal, rabbit, 1:1000 | XP®, #70512, clone E2G6Y, Cell Signaling Technology |

*: if available

^#^: This antibody was applied only in experiments involving TKA organoids subjected to CRISPR/Cas9-mediated editing of the *Zeb1* gene
