# Supplementary material for: Canonical TGFβ signaling induces collective invasion in colorectal carcinogenesis through a Snail1- and Zeb1-independent partial EMT
Source: Oncogene. 2022 Jan 24;41(10):1492–506. doi: 10.1038/s41388-022-02190-4 (PMC8897192; doi:10.1038/s41388-022-02190-4)
Supplement: Supplementary file 8 — Supplementary table 6_Flum et al [file 41388_2022_2190_MOESM8_ESM.docx]

**Supplementary table 6: List of primer sequences used for qRT-PCR**

| **Gene** | **forward (5´ - 3´)** | **reverse (5´ - 3´)** |
| --- | --- | --- |
| Aldh1a1 | GGTGGTGTGGGTTAACTGCT | GCGACTGTCTTGAGCTCAGT |
| Ascl2 | GGCTGTTAACACCCGCTACT | CTTTCCTCCGACGAGTAGGC |
| Axin2 | TCCCCACCTTGAATGAAGAA | TGGTGGCTGGTGCAAAGA |
| Cd44 | GCACTGTGACTCATGGATCC | TTCTGGAATCTGAGGTCTCC |
| Cd133 | TGTGGATGTCATCAAAGACG | GGGCAGCTCCTGGTTTAAGT |
| Cdh1 | CTTTAAGCCCAGCACTCAGG | CCTGCTTCCTGAGAAAATGC |
| Cdh2 | GGGAATCAGACGGCTAGACG | TCAGCAGCTTTAAGGCCCTC |
| Cdx2 | GAGTCCTGTGACCTCCTTGC | GGATTCTCGCAGCGTCCATA |
| Chga | GCCCGAAGTGACTTTGAGGA | CTACTCGAGCAGCAGTCTGG |
| Eef1a1 | GACAGCAAAAACGACCCACC | GGGCCATCTTCCAGCTTCTT |
| Epcam | ATCGCTGTCATTGTGGTGGT | CTCAGCACGGCTAGGCATTA |
| Ephb2 | CCAGCTTTAACACGGTGGAT | CCAGCTAGAGTGACCCCAAC |
| Ephb3 | GGTTTGCATCCTTTGACCTG | CTCGTTGGAGCTGAGTGTCA |
| Fn1 | TGACGCTGGCTTTAAGCTCA | TCATCCGCTGGCCATTTTCT |
| Foxa1 | AACCTCATGTCCTCCTCCGA | GCACGGGTCTGGAATACACA |
| Gapdh | ACCACAGTCCATGCCATCACT | GTCCACCACCCTGTTGCTGTA |
| Itga5 | ATTTCCGAGTCTGGGCCAAG | GATCCACAACGGGACACCAT |
| Itgav | AGCGCAATCCTGTACGTGAA | CTGAATGCCCCAGGTGATGT |
| Itgb1 | GCGTGGTTGCTGGAATTGTT | AGGATTTTCACCCGTGTCCC |
| Krt20 | TAAAGACCCGGCTTGAGCAG | TTCAGAGGACACGACCTTGC |
| Lef1 | CAAGCGCCGACTTCCAAAAA | GAAGATGCTGGAGGATCGCA |
| Lgr5 | GGAAGACCTGAAGGCCCTTC | TCTGAACACGGTCAAAGCCA |
| Lyz | AGAGGGTGGTGAGAGATCCC | GGGAAAGCGAGGAAGTGTGA |
| Muc2 | GGATCACAGGTGCTCTTGCT | GCACAGACAGCTCTCGATGT |
| Nanog | CGGTGGCAGAAAAACCAGTG | AAGGCTTCCAGATGCGTTCA |
| Ocln | CCTCCACCCCCATCTGACTA | GGAATCTCCTGGGCCACTTC |
| Pou5f1 | GAGAAGTGGGTGGAGGAAGC | CTCCACCTCACACGGTTCTC |
| Snai1 | CTTGTGTCTGCACGACCTGT | CTTCACATCCGAGTGGGTTT |
| Snai2 | CCTTTCTCTTGCCCTCACTG | ACAGCAGCCAGACTCCTCAT |
| Sox2 | CATGGGCTCTGTGGTCAAGT | CGGGGAGGTACATGCTGATC |
| Twist1 | CCCCACTTTTTGACGAAGAA | CAGTTTGATCCCAGCGTTTT |
| Twist2 | ACAACCTTGTGGCTCCTCAT | ACCCAGGAGGAAAATCCAAC |
| Zeb1 | GGGGCATCTCACACTTTTGT | AACGGCTGTGAACCAAAAAC |
| Zeb2 | TGGCCTATACCTACCCAACG | TTCGAGACAGACAGGAATCG |
